# How to remove or control confounds in predictive models, with applications to brain biomarkers

**DOI:** 10.1093/gigascience/giac014

**Published:** 2022-03-12

**Authors:** Darya Chyzhyk, Gaël Varoquaux, Michael Milham, Bertrand Thirion

**Affiliations:** Parietal project-team, INRIA Saclay-île de France, France; CEA/Neurospin bât 145, 91191 Gif-Sur-Yvette, France; Center for the Developing Brain, Child Mind Institute, New York, NY 10022, USA; Parietal project-team, INRIA Saclay-île de France, France; CEA/Neurospin bât 145, 91191 Gif-Sur-Yvette, France; Center for the Developing Brain, Child Mind Institute, New York, NY 10022, USA; Center for Biomedical Imaging and Neuromodulation, Nathan S. Kline Institute for Psychiatric Research, Orangeburg, NY 10962, USA; Parietal project-team, INRIA Saclay-île de France, France; CEA/Neurospin bât 145, 91191 Gif-Sur-Yvette, France

**Keywords:** confound, subsampling, phenotype, predictive models, biomarkers, statistical testing, deconfounding

## Abstract

**Background:**

With increasing data sizes and more easily available computational methods, neurosciences rely more and more on predictive modeling with machine learning, e.g., to extract disease biomarkers. Yet, a successful prediction may capture a confounding effect correlated with the outcome instead of brain features specific to the outcome of interest. For instance, because patients tend to move more in the scanner than controls, imaging biomarkers of a disease condition may mostly reflect head motion, leading to inefficient use of resources and wrong interpretation of the biomarkers.

**Results:**

Here we study how to adapt statistical methods that control for confounds to predictive modeling settings. We review how to train predictors that are not driven by such spurious effects. We also show how to measure the unbiased predictive accuracy of these biomarkers, based on a confounded dataset. For this purpose, cross-validation must be modified to account for the nuisance effect. To guide understanding and practical recommendations, we apply various strategies to assess predictive models in the presence of confounds on simulated data and population brain imaging settings. Theoretical and empirical studies show that deconfounding should not be applied to the train and test data jointly: modeling the effect of confounds, on the training data only, should instead be decoupled from removing confounds.

**Conclusions:**

Cross-validation that isolates nuisance effects gives an additional piece of information: confound-free prediction accuracy.

## Introduction

Predictive models, using machine learning, are becoming a standard tool for scientific inference. In cognitive neuroscience, they can be used for “decoding,” to make conclusions on mental processes given observed brain activity [1–3]. With the rise of large-scale brain-imaging cohorts, they can extract imaging biomarkers that predict across subjects phenotypes such as neuropsychiatric conditions [4–6] or individual traits [7,8].

A crucial aspect of these biomarkers is their ability to predict the outcome of interest, i.e., to generalize to new data [9]. However, these predictions can be driven by confounding effects. Such effects affect both the brain-imaging data and the prediction target but are considered irrelevant. For instance, brain imaging reflects age quite accurately and actually carries information about age-related diseases [8,10,11], yet [12] showed that participants’ in-scanner motion varies with age and it creates systematic differences in recorded brain imaging signals. Given this confounding effect, MRI biomarkers of brain aging may be nothing more than expensive measurements of head motion. Other examples may be more subtle: age matters for diagnosing Alzheimer disease, yet an important question is whether brain imaging yields an accurate diagnosis of Alzheimer disease beyond the mere effect of age.

More generally, the data at hand often capture effects not of direct interest to the investigation. In many situations, some confounds such as head motion cannot be fully avoided. To make matters worse, large cohorts developed in population imaging to answer epidemiological questions (e.g., UK Biobank [13]) are observational data: there is no controlled intervention or balanced case-control group; rather, individuals are recruited from diverse populations with various sampling or selection biases. To conclude on the practical use of biomarkers, it is important to ensure that their predictions are not fully driven by such unwanted effects. This requires measuring model predictive accuracy after controlling for nuisance variables. Confounding effects can also make it hard to interpret brain-behavior relationships revealed by predictive models [14] because confounds can mediate the observed association or be a latent common cause of observations [15].

In experimental settings, e.g., as in a small cohort, confounding can be suppressed by balancing the acquisition for confounds, or using randomized controlled trials. However, constraints in the data acquisition, e.g., recruitment of a large cohort, often imply that confounds are present in the data, and appropriate analysis is needed to avoid reaching erroneous conclusions. The statistical literature on controlling confounding variables is well developed for classic statistical analysis, such as statistical testing in a linear model at the heart of the standard mass-univariate brain mapping [16,17]. However, these procedures need to be adapted to high-dimensional predictive-modeling settings, where the focus is to achieve high-prediction accuracy based on imaging data. Indeed, predictive models do not rely on the same parametric assumptions, namely, linearity of effects and Gaussian noise. Often, a predictive analysis does not build on a generative model of the signal but on optimizing discrimination [18]. In addition, predictive models draw their purpose and validity from out-of-sample prediction rather than in-sample statistical testing [19]. The question tackled here is thus whether one can assess the predictive accuracy of brain measurements free of unwanted confounds. It is *not* to identify treatment effect size nor to perform other types of causal inference.

In this article, we study statistical tools to control for confounding effects in predictive models. We consider that practitioners should primarily avoid or reduce the impact of confounds on their model, but this is not always feasible or may be hard to check; hence, we choose to put the emphasis on the unbiased evaluation of models in the presence of confounds. A preliminary version of the work discussed here was presented at the PRNI conference [20]. While the core method is the same, it presents limited insights on the theoretical underpinnings and practical value of the method proposed. Experiments on simulated data are absent and experiments on neuroimaging data are limited to just 1 dataset. In particular, statistical significance is not established thoroughly, and only 1 alternative approach is considered. In short the conference publication provides limited insights on the method, while the present work provides a complete description and points to the code for reuse.

We first review how the classic deconfounding procedures can be used in predictive-modeling settings, i.e., together with cross-validation. We then expose a complementary approach that is not based on removing confounding effects but rather testing whether a given predictive model—e.g., a biomarker—predicts well when these confounds are not present. For this we introduce the “confound-isolating cross-validation” method, which consists in sampling test sets in which the effect of interest is independent from the confounding effect. The benefits of this approach are that it is non-parametric and that it directly tests the quantity of interest in a predictive analysis. We then run an extensive empirical study on 3 population-imaging biomarker extraction problems and a tabular dataset, as well as simulations. We draw practical recommendations to test predictive models in the presence of confounding effects.

## Methods: controlling for confounds in predictive models

### Formalizing the problem of prediction with a confound

#### Assessing predictive models

**Figure 1:**
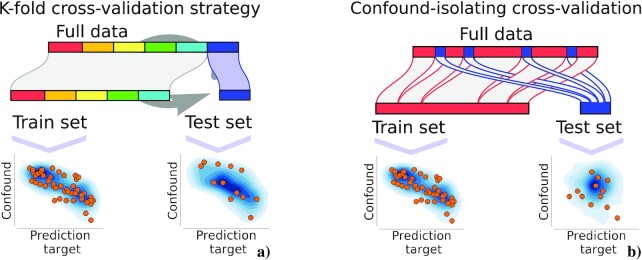
: Classic and confound-isolating cross-validation. (a) *k*-fold cross-validation is the common procedure to evaluate predictive models. It consists in splitting the data into *k* equal groups. Then *k* − 1 folds are used to fit a model and 1 fold is used to validate the model. This process is repeated *k* times so that each sample is taken once in the test set. (b) In “confound-isolating cross-validation” sampling we divide the data into train and test sets but in a different way. First, using subsampling, we create a test set on which **y** and **z** are independent. The train test is constructed from the rest of the samples that are not included in the test set. In this way, the method creates a test set that contains unrelated target and confound.

Predictive models are assessed by their prediction accuracy [19]. For this, cross-validation is the standard tool, typically *k*-fold cross-validation [21]. It consists in partitioning (potentially randomly) the original dataset into *k* equal size subsets or folds (each denoted by a color in Fig. 1). One of these *k* sets is held out for testing, and the remaining (*k* − 1) folds are used for training the model. This process is repeated *k* times, where each time a different group of observations compose the test set. Prediction accuracy is measured on the test set, then averaged across folds.

#### Confounding variables in a prediction task

To formalize prediction in the presence of a confound, we consider a dataset of *n* observations—e.g., participants or time points—comprising *p*-dimensional brain signals ${\bf X} \in \mathbb {R}^{n \times p}$, an effect of interest (in classification settings, **y** does not take continuous values in $\mathbb{R}^n$, yet we use the most general notation to cover both classification and regression settings) ${\bf y} \in \mathbb {R}^n$ (the biomarker target), and a confounding effect ${\bf z} \in \mathbb {R}^n$.

An imaging biomarker then predicts **y** from **X**. If **X** and **z** on the one hand, **y** and **z** on the other hand, are not independent, the prediction of the target **y** might be affected or most accurately done by the confounding effect, **z**. Such prediction may be misleading or useless. It can be misleading because it can be interpreted as a link between brain structures and **y** (e.g., fluid intelligence) while such a link only reflects the effect of **z** (e.g., age). It can be useless because brain imaging is likely much more costly to acquire than the phenotypic variable **z**; hence it should be used only if it brings more diagnostic information. Moreover, this can be detrimental to accuracy: if a future dataset shows an altered relation between the confound and the features, prediction accuracy may be compromised.

A crucial problem for the validity of the biomarker is to measure whether it can predict **y** from **X** and not solely from **z**. Prediction accuracy is ideally measured on an independent validation set, but most often, no large independent validation set is available and a cross-validation procedure, which iteratively separates train and test sets [21], is used. Little et al. [22] discuss what cross-validation captures in the presence of a confounding variable. Although there can be many possible confounds in brain imaging (see section Defining confounds calls for modeling choices), we focus below on simple settings, assuming that the main confounding factor has been isolated in 1 variable.

There are 2 points of view to controlling confounds in predictive models. One is to try and remove the effect of the confounding variables from the data, by regressing them out (deconfounding) or resampling the data to cancel spurious correlations (rebalancing). The other is to test that the model’s prediction captures more than the confound. Removing the confounding signal can test whether predictions are fully driven by the confound **z** rather than the brain signal **X**. However, it does not provide a good tool to measure the predictive power in the presence of confounds: the accuracy is likely biased, as illustrated in the simulations.

Another point of view on confounding effects in predictive modeling consists in trying to learn a predictor from a biased population (one that has the confounding effect) that differs from the population of interest (one without the confounding effect). The problem can then be tackled as a “domain adaptation” problem [23,24]. However, Rao et al. [24] have shown that compensating for the confound does not improve prediction if the test population is not markedly different from the training population. Note that train and test samples are often drawn from the same population, either because only 1 cohort is available or because a proper stratification scheme is used. Our question is different: we are interested in assessing whether learning a biomarker on a confounded dataset leads to predictions that are fully driven by the confound.

### Deconfounding

#### Deconfounding in standard analysis

In inferential statistics—as opposed to predictive modeling—proper modeling of confounds is important to control the interpretation of model parameters, ensuring that they are not driven by the confounding effects. Classical statistic analysis in brain imaging is based on the general linear model (GLM) [16, 25], in which confounding effects are controlled by additional regressors to capture the corresponding variance. Such an approach shows limitations in predictive-modeling settings. First, it is based on maximum-likelihood estimates of linear models, while in general, predictive models are not explicitly based on a likelihood and are often not linear. Second, it is designed to control in-sample properties, while predictive models are designed for out-of-sample prediction. The 2-step approach based on applying a classical GLM to remove the confounding effect, then a predictive model, may lead to pessimistic results, e.g., below-chance prediction [8,26].

In the context of the GLM, an alternative implementation relies on removing the effect of variables that are correlated [25]. Note that in all this work we assume that the confounder is associated with **X** and **y** without creating 3-way interactions between **X, y**, and **z**. Given a sample ${\bf X}\in \mathbb {R}^{n\times p}$ of *n* observations (participants) with *p* brain imaging features (e.g., connectivity matrices), **X**_*i*_ = (**X**_*i*1_, **X**_*i*2_,..., **X**_*ip*_) and confounds ${\bf z}\in \mathbb {R}^{n}$, the model is:

(1) \begin{eqnarray*}
\mathbf {X = z^{T}w + e} , \end{eqnarray*}

where **w** is a vector of weights (1 per voxel, ${\bf w}\in \mathbb {R}^{p}$). $\hat{{\bf w}}$ represents the estimated coefficients, which are obtained typically through least-squares regression:

(2) \begin{eqnarray*}
\mathbf {\hat{w} = (z^{T}z)^{-1} z^{T} X }. \end{eqnarray*}

Given these equations, a linear model can be used prior to the predictive model to remove the effect of the confounds **z** on the brain signals **X**. It must be adapted to out-of-sample testing. One solution is to apply deconfounding jointly on the train and the test set, but it breaks the statistical validity of cross-validation because it couples the train and the test set [21]. Hence it can give biased results.

#### Out-of-sample deconfounding

To adapt the above “deconfounding” approach to the 2 phases of training and testing a predictive model, a useful view is to consider the deconfounding model as a predictive encoding model, predicting a fraction of the signal **X** from **z**. Deconfounding is then performed by removing the part of the signal captured by **z** from **X**:

(3) \begin{eqnarray*}
\mathbf {\hat{X}_{clean} = X - z\hat{w} } , \end{eqnarray*}

where $\hat{{\bf w}}$ are the coefficients of the linear deconfounding model (Eq. 1), estimated on the train data with Equation 2 and then applied to the test [26]. The full out-of-sample deconfounding procedure is listed in Algorithm 1.

**Figure:**
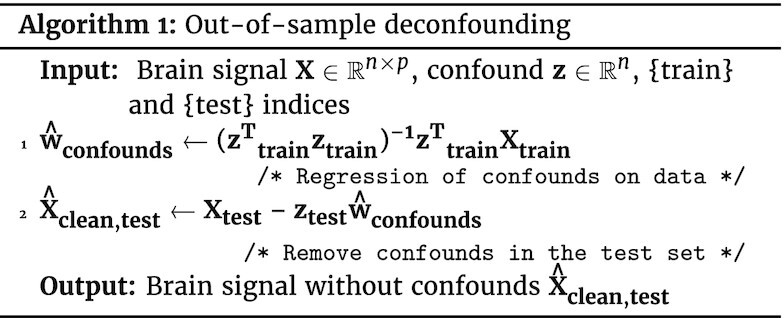


A drawback of such deconfounding is that it is strongly parametric; i.e., it relies on the model of confounds used. Equation 2 stands for the classic linear model, assuming linearity between the confounding variable **z** and the brain signal **X**. The linear model only takes into account second-order statistics (covariance or correlations) and ignores more complex dependencies.

#### Model-agnostic out-of-sample deconfounding

A common solution to go beyond linear effects of confounds is to use a polynomial expansion of the confounds **z** in the linear deconfounding model. Another option is to use a more powerful predictive model in the confound removal. A predictive model—including a mere linear model—regressing **X** on **z** can be seen as estimating a function *f* so that $f({\bf z}) = \mathbb {E}[{\bf X}|{\bf z}]$. There are many possibilities such as random forests or Gaussian processes. The procedure used for out-of-sample deconfounding can then be adapted as in Algorithm 2. While this approach is powerful, it risks also removing part of the signal of interest. Indeed, using a more powerful predictive model, e.g., a higher-order polynomial, leads to explaining in **X** more data as a function of **z**; however excessively powerful models “overfit”, which means that they explain variance in **X** by chance. In such a situation, the deconfounding procedure may remove signal of interest, unrelated to the confound.

**Figure:**
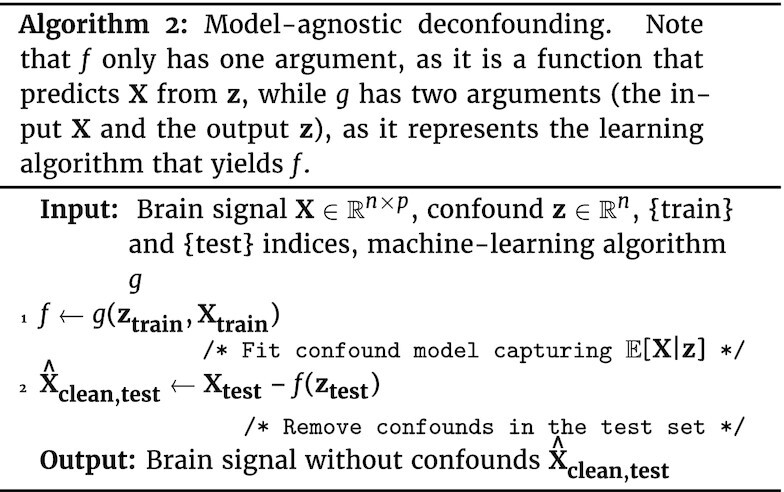


### Comparing predictive power of confounds

A simple evaluation of the effect of **z** on the prediction of **y** is to use predictive models predicting **y** from **z** (“prediction from confound”) and compare the predictive accuracy to that obtained with biomarkers based on brain signals. This argument is used by Abraham et al. [6] to control for the effect of movement on autism diagnostics.

### Creating a test set to isolate the confounding effect

Rather than deconfounding, the investigator can ensure that the predictive model is useful by measuring its accuracy on a dataset where the confounding effect is absent. In a cross-validation setting, such a situation can be created by using as a test set a well-chosen subset of the data that isolates the confounding effect (see Fig. 1 for a graphical illustration of the approach). Formally, it requires choosing a subset $\mathcal {S}$ of the data such that ${\bf y}_\mathcal {S}$ and ${\bf z}_\mathcal {S}$ are independent (the feasibility of this subset creation is discussed below).

The remainder of the data are used as a training set to learn to predict **y** from **X**. If the prediction generalizes to the test set $\mathcal {S}$, the learned relationship between **X** and **y** is not entirely mediated by **z**. In particular, the prediction accuracy then measures the gain in prediction brought by **X**.

#### Categorical confound

The confounding effect can be “categorical,” e.g., the site effect when learning predictive biomarkers on multi-site acquisitions as in [6]. In such settings, to test that the model can indeed predict independently from site effects, a simple solution is to resort to a cross-validation that avoids having samples from the same site in both the train and the test sets. This may imply resampling the data to cancel out associations between site and target related to data imbalance. Similarly, in multi-participant prediction with repeated measurements from the same participant, participant-wise cross-validation can rule out that prediction is driven by participant identification [22,27]. More generally, for a categorical confound **z**, having distinct values for **z** in the train and the test set ensures that the prediction cannot be driven by **z**. We note that this procedure is different from the stratification strategy used in classical statistics, but it clearly avoids any bias due to imperfectly corrected association between **z** and the other variables. In the case of site-related confounds, prediction accuracy will obviously decrease. This can be addressed with techniques such as invariant risk minimization [28], but we do not further consider this approach here.

#### Continuous confound

When **z** is a continuous variable, such as age, it is more challenging to generate test sets on which ${\bf y}_\mathcal {S}$ and ${\bf z}_\mathcal {S}$ are independent. We describe here an algorithm to generate such sampling, “confound-isolating cross-validation” subsampling. It is based on iterative sampling to match a desired distribution: the goal is to have a test set with independence between **y** and **z**, i.e., $p({\bf y},{\bf z}) = p({\bf y})\, p({\bf z})$, where *p*((**y, z**)) is the joint probability function of **y** and **z**, and *p*(**y**) and *p*(**z**) are the marginal probability distribution.

A related quantity is mutual information, which characterizes the level of dependency between the 2 variables: $\eta ({\bf y},{\bf z}) = \mathbb {E}\left[\log \left({p(({\bf y},{\bf z}))} / {p({\bf y}) p({\bf z})}\right)\right]$. In practice we estimate the probability density functions with a kernel-density estimator (KDE) using Gaussian kernels. We iteratively create the test $\mathcal {S}$ set by removing participants; at each iteration, we consider the problem as a distribution matching problem, matching $p({\bf y}_\mathcal {S},{\bf z}_\mathcal {S})$ and $p({\bf y}_\mathcal {S})\, p({\bf z}_\mathcal {S})$. For this, we use importance sampling: we randomly draw 4 participants to discard with a probability $p({\bf y}_\mathcal {S},{\bf z}_\mathcal {S})/[p({\bf y}_\mathcal {S})\, p({\bf z}_\mathcal {S})]$ using the inverse sampling method (sec 2.2 of [29]). Algorithm 3 gives the details. The choice of 4 samples is tailored to the sample size considered here: it makes the algorithm faster than using 1 sample yet is low enough not to compromise mutual information minimization. A Python implementation is available on GitHub [30] and on PyPI repository [31] and can be installed with pip install confound-prediction.

**Figure:**
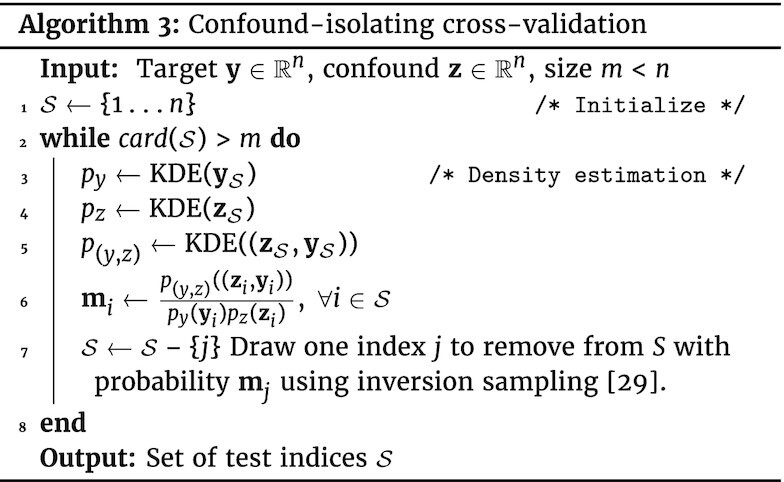


Note that if **z** and **y** are too strongly related, the aforementioned subsampling procedure does not have enough degrees of freedom and may always chose the same subset: the test set would be deterministically defined by the sampling procedures, in which case there would effectively be only 1 fold of cross-validation. In practice, it is important to check that such a situation does not occur when analyzing a given dataset. One way is to compute the average fraction of common samples between 2 test sets created with different seeds. Because this value ranges from 0 to 1, where 1 means that all test sets contain the same samples and 0 that test sets have no sample in common, it is important to check that it is low enough.

## Empirical Study Methodology

We herein describe the experimental materials underlying our empirical study of confound-controlling approaches in predictive models.

### Simulation studies

To understand the behavior of the different accuracy scores, we present experiments on simulated data. We simulate a dataset ${\bf X_0} \sim \mathcal {N}(0, 1)$ with confound ${\bf z_0} \sim \mathcal {N}(0, 1)$ to predict continuous variable ${\bf y} \sim \mathcal {N}(0, 1)$. We evaluate 2 sample sizes: *n* = 100 and *n* = 1,000. We use *p* = 100 features in **X_0_**. We study 3 scenarios:

- No direct link between target and brain, where the brain signal does not provide any direct information to predict **y** but is observed with a confound linked to **y**:
  \begin{eqnarray*}
&\mathrm{observed\,\,confound}\ {\bf z} = {\bf y} + {\bf z_0},\\ &\mathrm{observed\,\,signal}\ {\bf X} = {\bf X_0} + {\bf z}. \end{eqnarray*}
- Direct link between target and brain, where the brain signal does indeed provide information to predict **y** and has an additional confound linked to **y**:
  \begin{eqnarray*}
& \mbox{observed confound}\ {\bf z} = {\bf y} + {\bf z_0},\\ &\mbox{observed signal}\ {\bf X} = {\bf X_0} + {\bf y} + {\bf z}. \end{eqnarray*}
- Weak confound and direct link between target and brain:  
  \begin{eqnarray*}
& \mbox{observed confound}\ {\bf z} = 0.5\, {\bf y} + {\bf z_0},\\ & \mbox{observed signal}\ {\bf X} = {\bf X_0} + {\bf y} + {\bf z_0}. \end{eqnarray*}

Note that one could consider instead a canonical scheme in which **z** would cause **x** and **y**. Because our work is not on causal inference per se, we aim at a statistical procedure that does not require a prescribed causal relationship between the variables, which is often unknown.

### Two classic confounded predictions in population imaging

#### Motion confounding brain-age prediction

**Figure 2:**
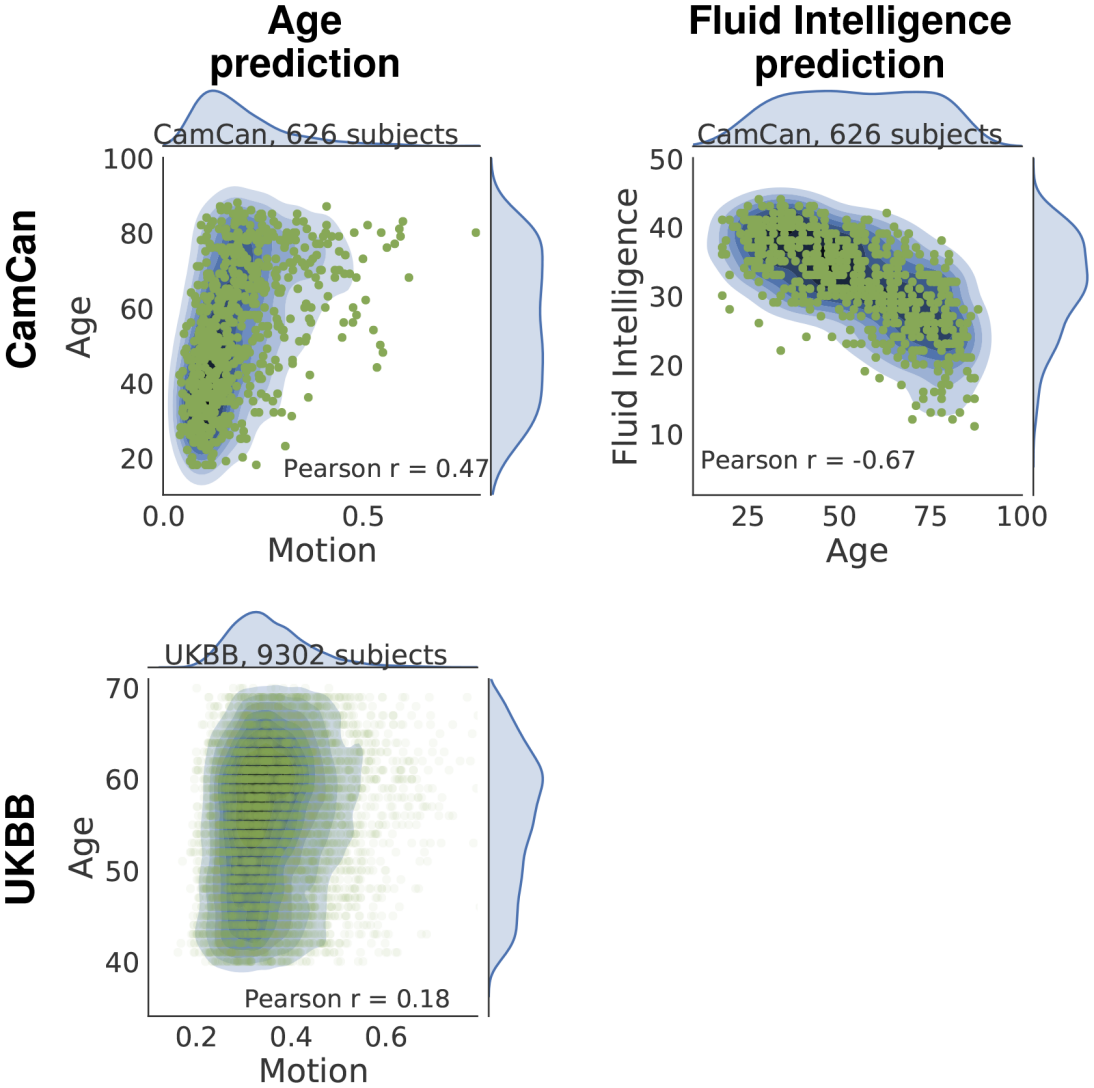
: Joint distribution of target and confound. The first column presents the scatter plot of age and motion variable for CamCan (top) and UKBB (bottom). The second column shows the case of fluid intelligence prediction with age as confound for CamCan. In all cases, the target is clearly associated with the confound; the corresponding *P*-values are <10^−5^.

Because brain aging is a risk factor of many diseases, the prediction of brain age from MRI is a promising biomarker [11]. In childhood also, markers of functional brain development can help to reveal neurodevelopmental disorders [32,33]. Many recent studies report age prediction, e.g., from resting-state functional connectivity [7,32,34], from structural imaging [35], or combining multiple imaging modalities [8,10]. However, older people and children move more in the scanner than young adults (see Fig.  2 [12,36–38]). Thus, age-related changes observed in brain images may be confounded by head motion [39] and image quality [40].

Indeed, in-scanner motion creates complex MRI artifacts that are difficult to remove [39]. In addition, they severely affect measurements of functional connectivity [41].

Here the confounding effect is that of head motion during the few hundreds of scans of individual acquisitions. To build a variable summarizing head motion for each participant, we use the movement time series computed during preprocessing. As suggested in [41], we create the confound **z** from the root mean squared displacements (position differences across consecutive time points) for each participant $z = \left\{ \frac{1}{I-1}\sum _{i=2}^{I} \left[ (t_x^i - t_x^{i-1})^2 + (t_y^i - t_y^{i-1})^2 + (t_z^i - t_z^{i-1})^2 \right] \right\} ^{ 1/2}$, where *t_x_* is left/right, *t_y_* anterior/posterior, and *t_z_* superior/inferior translation and *i* ∈ [[*I*]] is the time index. The prediction target **y** is the age in years.

#### Age confounding fluid intelligence measures

Various studies have predicted individual cognitive abilities from brain functional connectivity [42,43]. In particular, [43] used machine learning to predict fluid intelligence from rest fMRI. Fluid intelligence quantifies the ability to solve novel problems independently from accumulated knowledge, as opposed to crystallized intelligence, which involves experience and previous knowledge [44]. It is well known that cognitive abilities change with age [45–48], in particular that fluid Intelligence progressively declines in middle age [49], while crystallized intelligence continues to grow with age. Indeed, in a cohort with a large age span, the data display a strong relation between fluid intelligence and age (Fig. 2). When extracting biomarkers of fluid intelligence, the danger is therefore to simply measure age. We study how to control the effect of age when predicting a fluid intelligence score from rest-fMRI functional connectivity.

### Population-imaging rest-fMRI datasets

#### Datasets

We ran experiments on 626 participants from the CamCan dataset and 9,302 participants from UKBB. All participants are healthy with no neurological disorders.

- Cambridge Center for Ageing and Neuroscience (CamCan) data [50] study age-related changes in cognition and brain anatomy and function. Characteristics of interest of this dataset are (i) a population lifespan of 18-88 years and (ii) a large pool (626 participants) of multi-modal MRI data and neurocognitive phenotypes.
- The UK Biobank (UKBB) project [51] is a prospective epidemiological study to elucidate the development of diseases of the UK population over the years. The data used here contain 9,302 participants from the first release of the UKBB ongoing cohort study with available resting-state fMRI scans and extensive health and lifestyle information [52,53].

Table 1 presents detailed information about the number of participants and the scale of the scores for each dataset.

**Table 1.** Characteristics of the datasets

Dataset information	CamCan	UKBB
No. of participants	626	9,302
Age range, y	18−88	40−70
Fluid intelligence scale^1^	Cattell	UKBB-designed

1 Scores for fluid intelligence differ on the 2 datasets: CamCan uses the Cattell test (11−44 scores), and UKBB a specifically designed touch-screen questionnaire (1−13 scores).

We give detailed information on preprocessing steps for each dataset in Appendix 8 of Nichols et al., following COBIDAS recommendations [54].

#### Prediction from functional connectivity

To build predictive models from resting-state fMRI, we follow the recommendations of Dadi et al. [55]. We use the BASC functional atlas [56] with 64 regions, based on which we extract fMRI time series from the CamCAN dataset. Next, we normalize, detrend, and bandpass-filter the signal to between 0.01 and 0.1 Hz. We represent connectivity matrices with tangent parameterization [57]. Finally, we use a ridge regression with nested cross-validation to learn predictive biomarkers from the functional-connectivity matrices. We use Nilearn [58] for the whole predictive pipeline.

### Tabular (non-imaging) data

The considerations on confounds in predictive models are not specific to imaging data. We also study a confounded prediction without brain signals: on the UKBB data, we consider predicting an individual’s income from sociodemographic characteristics and mental health assessments. We investigate education as a potential confound: it may be reflected both in mental health and in income. There are 8,556 individuals with no missing values on the outcome and confound. We use random forests for prediction because it is a popular learner that is well suited to the distribution of these tabular data, which are often non-Gaussian and consist of categorical variables.

### Experimental paradigm: cross-validation measures

We use cross-validation to assess prediction accuracy. We consider 5 predictive frameworks: (1) without deconfounding, (2) deconfounding test and train sets jointly, (3) out-of-sampling deconfounding, (4) confound-isolating cross-validation, and (5) prediction from confounds only. The code for these various strategies to control for confounds can be found on GitHub [30] and on PyPI repository [31] and can be installed with pip install confound-prediction.

We use 10 folds, with random splits of 20% of the data in the test set. For confound-isolating cross-validation, different seeds in the random number generator lead to different folds. We assess the null distribution of predictions with permutations (20,000 folds on permuted labels **y**).

## Results of the Empirical Study

### Simulated data

**Figure 3:**
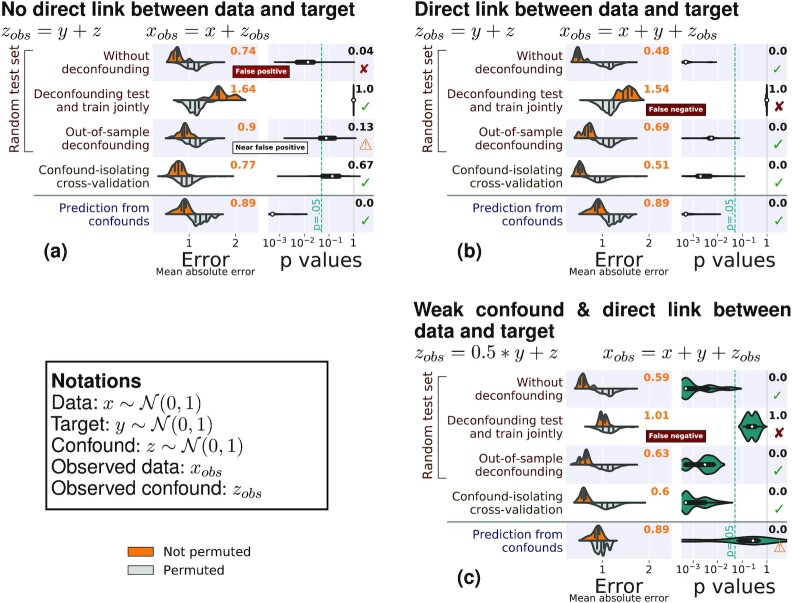
: Comparisons on simulated data. The left column of each subfigure reports the prediction performance as the mean absolute error for the 5 approaches considered: Prediction from the data without deconfounding, prediction after deconfounding test and train jointly, prediction with out-of-sample deconfounding, prediction with confound-isolating cross-validation, and prediction from confounds. The left column displays the distribution across validation folds for the actual data (top, orange) and for permuted data distribution (bottom, gray). The right column displays the distribution of *P*-values across folds, obtained by permutation, and the text yields the aggregated *P*-value across folds, testing whether prediction accuracy is better than chance. Test subsets always represent one-fifth of the whole dataset. There are 3 simulation settings: (a) No direct link between target and brain, (b) a direct link between target and brain in the presence of a confound, and (c) a weak confound and a direct link between target and brain. Green ticks indicate correct conclusions, red crosses mark incorrect ones, and warning signs the weak results.

We first consider simulated data, for which there is a ground truth. Figure 3 shows the results of the different methods to control for confounds on 3 different simulated cases (Fig. 8 gives results for the same simulations with 1,000 samples).

- In the case where there is no direct relationship between the data and the target, the performance of the prediction model should not be better than chance after controlling for the confound. Both joint deconfounding and confound-isolating cross-validation clearly reveal that all the prediction is mediated by the confound. Out-of-sample deconfounding displays a less clear signal, as there seems to be a slight prediction even after deconfounding, although it is not significant.
- For a direct link between the data and the target, joint deconfounding yields a false-negative result, in the sense that it fully removes the prediction from the brain signal: it is too aggressive in removing signal. Other approaches correctly support a successful prediction.
- For a weaker confounding signal, results are similar; however it is worth noting that the target can no longer be well predicted from the confound.

Overall, on the simulations, both out-of-sample deconfounding and confound-isolating cross-validation give reliable answers, while deconfounding the test and train jointly as well as measuring the prediction from confounds cannot be trusted.

### Experiments on resting-state fMRI data

#### Potential confounds

Figure 2 shows the relationships between target variable ***y*** and confounds ***z***. Fluid intelligence (target) is strongly negatively correlated with age (confound) on the CamCan dataset (second column of Fig. 2). Also, on the CamCan data, age and motion are very correlated (first column of Fig. 2). On the more homogeneous and larger UKBB sample (9,302 participants), this link is weaker.

#### Confound-isolating cross-validation

**Figure 4:**
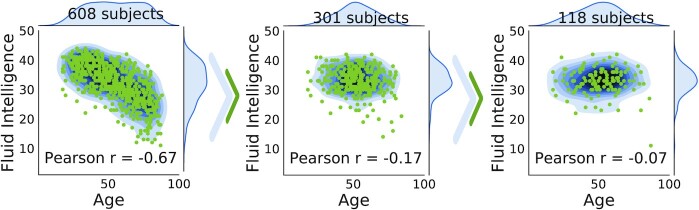
: Evolution of the test set created by confound-isolating cross-validation. The joint distribution of the target (fluid intelligence) and the confound (age) for the CamCan dataset is taken for demonstration. We show the process of selecting proper samples for the test set. We begin with the entire dataset; Pearson correlation is −0.67 with infinitesimal *P*-value (right subplot). After half of the iterations we have already reached a correlation −0.17 with *P* = 0.009 (middle subplot). The final test set is shown on the right subplot, correlation −0.007 with *P* = 0.02. It presents negligible residual dependency between targets and confounds.

**Figure 5:**
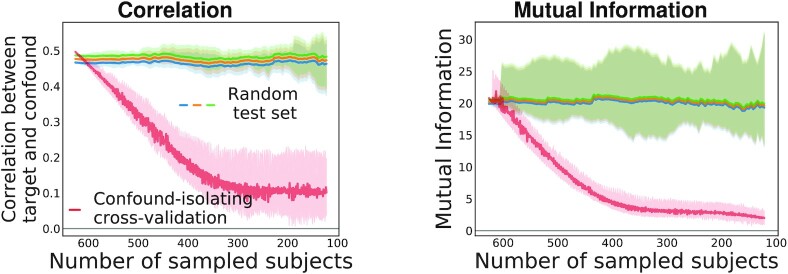
: Evolution of the link between confound and target with the number of participants for different subsampling methods on the CamCan dataset, considering age prediction. Applying Algorithm 3 effectively reduces statistical dependences between confound and target (red curve). In our experiments, we stop the sampling when the test set size is one-fifth of the dataset.

Figure 4 displays the evolution of the association between confound and target during confound-isolating cross-validation in the CamCan dataset, predicting fluid intelligence with age as a confound. In the full dataset, comprising 608 participants, the correlation between confound and target is ρ = −0.67. Iterating the algorithm to remove half of the participants leads to ρ = −0.17. The final test set contains one-fifth of the initial set of participants and achieves ρ = −0.07, showing that it indeed cancels the dependency between aging and motion. The joint distribution between target and confound displayed in Fig. 4 shows that the initial statistical dependency between these 2 variables vanishes after a few tens of iterations of the algorithm. Quantitative evaluation, measuring both Pearson correlation and mutual information (Fig. 5), confirms that the confound-isolating procedure efficiently creates a subset of the data without the dependency as soon as it reduces the data to ≤300 participants. Figure 9 shows similar success on the other prediction problems that we study.

In a cross-validation setting, the different test sets should probe different participants to maximize testing power. A risk, when using confound-isolating cross-validation, is that it could repeatedly generate test sets with the same samples. To measure the diversity of the test sets, we compute the average fraction of common samples between 2 tests sets created with different seeds. The value is in the range from 0 to 1, where 1 means that all test sets contain the same samples and 0 that test sets have no sample in common; the expected value is 1/5. We find an average intersection of 0.30 for age prediction with CamCan and 0.27 with UKBB; for fluid intelligence prediction with CamCan, we find 0.36. This demonstrates that the test sets do not repeat much, hence that there is no hidden determinism in the cross-validation scheme of the proposed method.

#### Testing for confounded prediction

**Figure 6:**
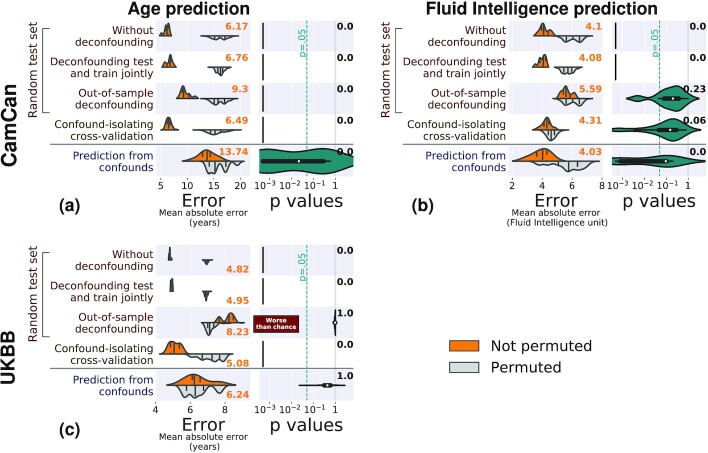
: Comparisons on population-imaging data. Each subfigure shows 1 prediction setting: (a) CamCan Age prediction, (b) CamCan fluid intelligence prediction, (c) UKBB age prediction. The left column of each subfigure reports the prediction performance as the mean absolute error for the 5 approaches considered: prediction from the data without deconfounding, prediction after deconfounding test and train jointly, prediction with out-of-sample deconfounding, prediction with confound-isolating cross-validation, and prediction from confounds. The left column displays the distribution across validation folds for the actual data (top, orange), and for permuted data distribution (bottom, gray). The right column displays the distribution of *P*-values across folds, obtained by permutation, and the text yields the aggregated *P*-value across folds (see main text), testing whether prediction accuracy is better than chance. Test subsets always represent one-fifth of the whole dataset. The figure clearly displays different behaviors across the 3 problems: without deconfounding, and deconfounding test and train jointly yield statistically significant but probably spurious accuracy; out-of-sample deconfounding can be overconservative (the prediction is worse than chance on UKBB), suggesting that the deconfounding model removes too much variance; confound-isolating cross-validation yields more nuanced results, and prediction from confounds yields variable results.

Figure 6 and Table 2 report the mean absolute error for the different approaches to control for confounds (mean absolute error is a good metric to compare across different test sets because it gives an absolute error measure in the unit of **y**, unlike explained variance, which depends on the variance of **y**). The figure also reports the *P*-value of predictive accuracy, from permutations (technically, there is one *P*-value per fold; to report only one number, we use *P*-value aggregation [59]). The first thing to note is that without controlling for confounding effects, all models lead to significant prediction. But are these driven by the confounds? Given that the various approaches measure predictions on different data, we compare how far these predictions are above chance, rather than their absolute value.

**Table 2.** Comparisons on population-imaging data, Camcan fluid intelligence prediction

Method	Mean absolute error ± σ		*P*-value
	Non-permuted	Permuted	
CamCan: Age prediction			
Without deconfounding	6.17 ± 0.43	16.00 ± 1.24	<0.001
Deconfounding test and train jointly	6.76 ± 0.53	16.24 ± 0.66	<0.001
Out-of-sample deconfounding	9.30 ± 0.80	15.79 ± 1.22	<0.001
Confound-isolating cross-validation	6.49 ± 0.46	15.21 ± 1.37	<0.001
Prediction from confounds	13.74 ± 1.50	15.22 ± 1.74	<0.001
CamCan: Fluid Intelligence prediction			
Without deconfounding	4.10 ± 0.29	6.05 ± 0.49	<0.001
Deconfounding test and train jointly	4.08 ± 0.22	5.70 ± 0.34	<0.001
Out-of-sample deconfounding	5.59 ± 0.32	6.04 ± 0.90	0.23
Confound-isolating cross-validation	4.31 ± 0.29	4.60 ± 0.30	0.06
Prediction from confounds	4.03 ± 0.60	5.70 ± 0.95	<0.001
UKBB: Age prediction			
Without deconfounding	4.82 ± 0.40	6.95 ± 0.80	<0.001
Deconfounding test and train jointly	4.95 ± 0.40	6.92 ± 0.80	<0.001
Out-of-sample deconfounding	8.23 ± 0.33	7.12 ± 0.34	>0.99
Confound-isolating cross-validation	5.08 ± 0.30	7.26 ± 0.60	<0.001
Prediction from confounds	6.24 ± 0.73	6.29 ± 0.72	>0.99
Tabular data: Income prediction			
Without deconfounding	0.79 ± 0.014	0.93 ± 0.016	<0.001
Deconfounding test and train jointly	0.79 ± 0.014	0.93 ± 0.016	<0.001
Out-of-sample deconfounding	0.77 ± 0.014	0.93 ± 0.016	<0.001
Confound-isolating cross-validation	0.85 ± 0.130	0.94 ± 0.180	>0.99
Prediction from confounds	0.87 ± 0.016	0.93 ± 0.016	<0.001

Deconfounding test and train sets jointly—removing the linear effect of the confounding variable on the full data– has little effect on the prediction performance on all datasets. On the other hand, out-of-sample deconfounding significantly changes prediction performance in a way that varies across tasks. Prediction accuracy of fluid intelligence on CamCan decreases to chance level. Age prediction on CamCan is little affected. However, age prediction on UKBB gives results worse than chance, i.e., worse than a model that learns to predict age on data where this relationship has been shuffled by permutation (see Fig. 6 and Table 2). Confound-isolating cross-validation also gives varying results on different datasets. For fluid intelligence prediction on CamCan, it also gives results at chance level. For age prediction on CamCan, it does significantly alter prediction accuracy, and on UKBB, it leads to a slightly worse prediction but still above chance. Finally, prediction from confounds leads to chance-level or good prediction of the target depending on the dataset. In particular, it does better than chance for fluid intelligence prediction.

These results show that in all these datasets, the confounds **z** are associated with both the data **X** and the target **y**. For fluid intelligence prediction on CamCan, all the prediction of **y** from **X** is mediated by **z**. However, for age prediction in CamCan, there exists within **X** some signal that is unrelated to **z** but predicts **y**. Age prediction in UKBB is a more subtle situation: **X** contains signals from **z** and **y** with shared variance, but there is enough signal beyond the effect of **z** to achieve a good prediction, as demonstrated by confound-isolating cross-validation, where the prediction cannot be driven by **z**. Yet, out-of-sample deconfounding removes the shared variance and hence creates predictions that are worse than chance.

### Tabular data

**Figure 7:**
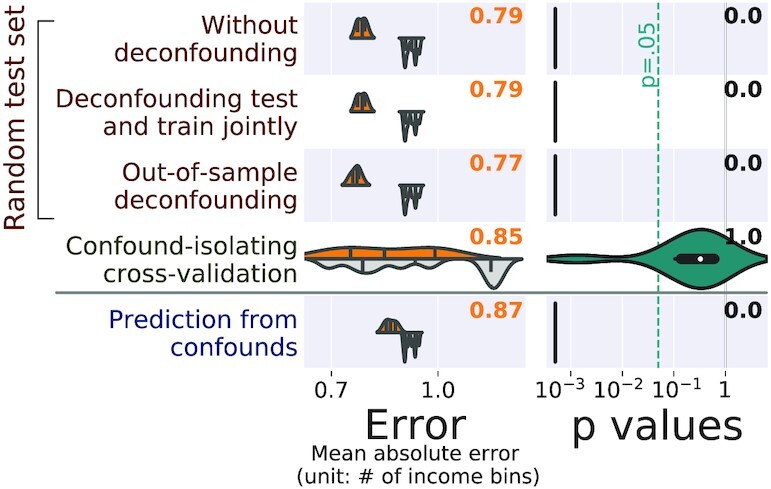
: Comparisons on tabular data: predicting income from sociodemographic characteristics and mental health, controlling for qualifications. The left column of the figure reports the prediction performance by the mean absolute error for the 5 approaches considered: prediction from the data without deconfounding, prediction after deconfounding test and train jointly, prediction with out-of-sample deconfounding, prediction with confound-isolating cross-validation, and prediction from confounds. The left column displays the distribution across validation folds for the actual data (top, orange), and for permuted data distribution (bottom, gray). The right column displays the distribution of *P*-values across folds, obtained by permutation, and the text yields the aggregated *P*-value across folds (see main text), testing whether prediction accuracy is better than chance.

Figure 7 and Table 2 give the results of analysis on the tabular data. There is a significant prediction of income from sociodemographic and mental health information, without any deconfounding. However, prediction from confounds shows that qualifications also predict income well. To control for qualification, deconfounding removes the signal explained by these in **X**. Here, deconfounding does not make the prediction worse; actually out-of-sample deconfounding improves it. Such an improvement can be explained if the deconfounding adds information about the confound to the signal rather than removing it, as can happen when the model of the confounds is mis-specified. To limit mis-specification issues, a random forest is used as the *g* function in Algorithm 2. Finally, confound-isolating cross-validation shows variable results, but overall that prediction does not work better than chance on balanced datasets, so that qualification is no longer specifically related to income.

**Figure 8:**
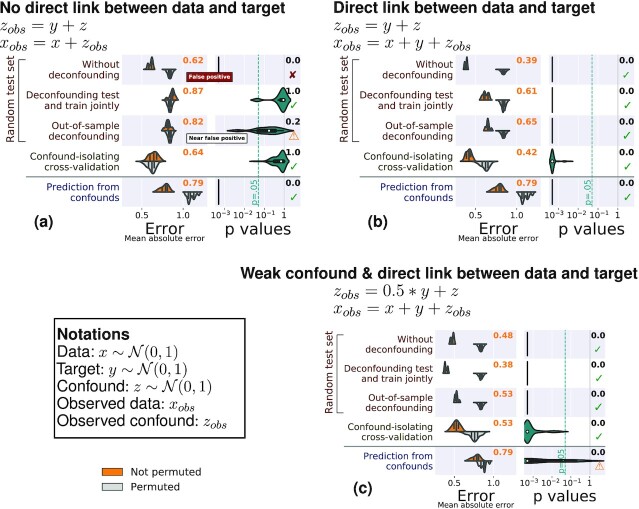
Benchmarking approaches to control confounded predictions on simulated data with many samples. The left column of each subfigure assesses the prediction performance through the mean absolute error (in signal units). We display the error distribution across validation folds for the data (top, orange) and for permuted data distribution (bottom, gray). The right column displays the distribution of P-values across folds, obtained by permutation, and the text reports the aggregated P-value across folds (see main text). Five approaches are benchmarked: without deconfounding, deconfounding test and train jointly, out-of-sampling deconfounding, confound-isolating cross-validation, and prediction from confounds. There are 3 simulation settings: (a) no direct link between target and brain, (b) a direct link between target and brain, and (c) a weak confound and a direct link between target and brain. Green ticks indicate correct conclusions, red crosses mark incorrect ones, and warning signs, the weak results.

**Figure 9:**
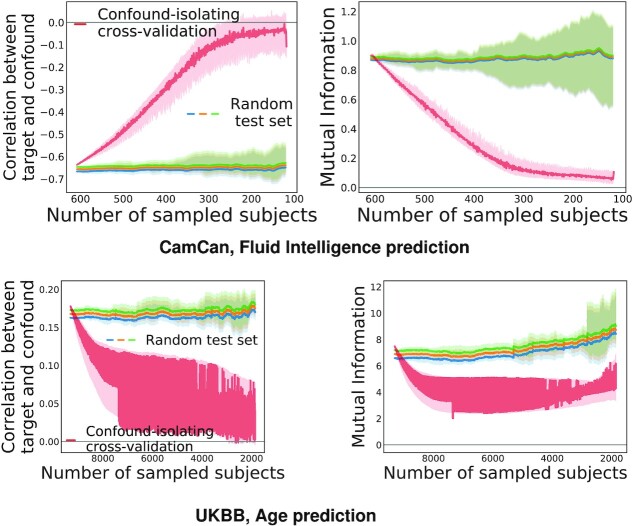
Evolution of mutual information and correlation with the number of participants for different subsampling methods on the CamCan dataset with fluid intelligence prediction and UKBB age prediction. This figure shows that the proposed method effectively reduces statistical dependencies between confound and target (red curve) for both datasets and both predictors.

Here, deconfounding leads to the conclusion that the prediction of income from sociodemographic and mental health information is not at all driven by qualifications while the other approaches suggest otherwise. The discrepancy is probably due to the complex non-linear interactions between these variables. The reality is probably that qualifications contribute to the prediction of income, as well as mental health and sociodemographic information, and that teasing out these contributions is hard.

## Discussion and Conclusion

Measuring the accuracy of predictive models, e.g., for biomarkers or brain decoding, must account for the presence of confounding effects that can contribute to the prediction. Indeed, an imaging biomarker that solely picks up head motion may detect diseases with some success but be overall a waste of scanner time. An accurate prediction of fluid intelligence from brain functional connectivity might simply be a consequence of indirectly capturing the participants’ age. Standard cross-validation procedures ignoring the confounds can overestimate prediction accuracy.

### Addressing confounds in predictive modeling

#### Approaches must be adapted to out-of-sample settings

Deconfounding approaches used in standard GLM-based analysis must be adapted to out-of-sample data by separating estimation of the confounds’ model from removal of the effect of confounds on the data, as detailed in Section Methods: controlling for confounds in predictive models and Algorithm 1. Importantly, applying deconfounding to the whole dataset without separating train and test set is not only wrong in theory—because it breaks independence of train and test data—but also leads to incorrect conclusions in practice, as clearly visible from the simulations.

Even done right, deconfounding in predictive settings can lead to pessimistic evaluations, as stressed by Snoek et al. [26] and shown in our experiments. This is because the signal explained by the confound is removed from the brain signal before it is passed to the predictive model. The corresponding correction can remove too much information when there is a large amount of shared signal between the confound and the target, e.g., aging and Alzheimer disease. Such problem does not arise in a GLM-based standard analysis because the confounds and the effects of interest are modeled *simultaneously*, and the consequences of shared signal are easier to handle.

To give a measure of predictive accuracy that is not pessimistic, we also study a different approach: testing the predictive model on a subset of the data crafted such that the effect of interest is independent from the confound. When the confounding effect is represented as a categorical variable, e.g., the effect of acquisition site, the approach can be simple because it amounts to splitting the data so as to ensure that generalization occurs for a category not observed in the training set. Creating an adequate test set for continuous confounds requires a dedicated method, as with confound-isolating cross-validation (Algorithm 3). It enables a test of the predictive power from brain imaging without discarding the potentially useful shared signal. In addition, it is non-parametric and does not rely on a linear confounding model. Empirical studies, on both brain-imaging data and simulations, show that both out-of-sample deconfounding and confound-isolating cross-validation can control correctly for confounds. Deconfounding before fitting a predictive model brings the benefit of building a predictor free of the confounding effect. However, it can remove shared variance and lead to pessimistic evaluations. Confound-isolating cross-validation brings the benefit of measuring the predictive power in the absence of the confounding effect. Such measure is of direct importance to gauge the practical value of a biomarker. As an attractive complementary approach, note that deep learning approaches for learning confound-free models have been proposed [60].

To summarize, our main claim is that it is possible to learn a confounded model yet evaluate it in an unbiased fashion. What matters in this logic is that the predictive accuracy after confound-isolating cross-validation remains better than chance, which amounts to performing an omnibus test of the variables of the model. The case where confound-isolating cross-validation would yield a null result certainly means that one should be cautious in claiming a conditional association between *X* and *y*, as slight variations in the confounding model may render the association significant or not: indeed the apparent association between features and target is dominated by the confounder and, thus, not a reliable one. In brief, this has an effect on the practical significance of claimed associations.

#### Which approach to use when: deconfounding versus confound-isolating cross-validation

Out-sample deconfounding and confound-isolating cross-validation give valid and complementary information. In the worst case, these approaches can be conservative, but they do not yield spurious associations. From a prediction perspective, when the training population reflects the target population adequately, changing the training data to remove the effect of the confounder may not improve prediction accuracy [24]. For instance, for many diseases, patients move more in the scanner than healthy individuals. Should an imaging biomarker of the pathology be developed, this effect will be most likely true in the population on which the biomarker is applied. Hence it is counterproductive to force the biomarker to discard this information. Rather, confound-isolating cross-validation should be used to check that the imaging biomarker does bring in value in addition to capturing motion.

On the other hand, confound-isolating cross-validation is not a universal remedy: removing a confounding effect from the training data may be useful if the confounder is incidentally correlated with **X** or **y** without any clear causal relationship. This is the case if the confounder is a feature of the measurement process. For instance, if the data are acquired across 2 imaging sites with different scanners, but 1 site recruited a much larger fraction of patients than the other, the risk is that the predictor learns to use information about the scanner rather than the disease. In such a case, the training strategy must be adapted, e.g., by removing the effect of the confound.

Finally, if the goal is to *interpret* successful prediction as evidence of a link between brain signals and the predicted outcome, modifying the training data is more likely to disentangle the biomarker pattern of interest from the confounding effect. In such a situation, deconfounding should be preferred, to give a model, with its parameters, that is not driven by the confounding signal.

#### Limitation: with many confounds the problem is harder

Here we have studied the case of 1, clearly identified, confound. The case of multiple confounds (e.g., age, education, sex, ethnicity) is more challenging. In such situations, deconfounding approaches may fully remove the signal of interest. For confound-isolating cross-validation, reliable estimation of mutual information will require much larger sample sizes than with a single confound. In practice, we recommend identifying the most effective confound to run confound-isolating cross-validation.

Another concern could be that such confounding factors are not well identified. In that case, the proposed approach does not help, but such a case is very hard to handle with statistical methods (see, e.g. [61]). We thus leave handling of imperfect confounder knowledge for future research.

### Elements to interpret analyses with confounds

#### Defining confounds calls for modeling choices

Whether a variable should be considered as a confounding effect or not is not dictated by the data but by the question at hand. The actual notion of confound comes from causal modeling, to give a causal interpretation to model parameters [15,62]. Confound variables are then chosen so as to model the difference between the measurements at hand and those obtained with a hypothetical intervention. Such choices are implicitly based on a model of which variables are causes or consequences of the fictional intervention and the outcome of interest (see [63] for guidelines in the case of UKBB).

In pure biomarker settings, the focus is not on potential interventions but on detecting or predicting an outcome. The concern is then that the measured accuracy might not reflect the actual application settings [22,27]. Here also, the choice of variables to control for must be governed by an understanding of how the data at hand may differ from ideal data to reflect the target application. More concretely, confounds can indeed relate to any aspect of the set-up, e.g., acquisition devices; data processing routines when these are not homogeneous across the entire dataset; measurement-related covariates such as motion; and individual conditions, such as age, sex, or genetics, that are correlated with the imaging variable and with the outcome.

#### Deconfounding for causal interpretations: the collider-bias danger

Using deconfounding to cancel the impact of a putative confound **z** removes any bias incurred by the spurious association between the data **X** and the prediction target **y**, when **z** is associated with both **X** and **y**. However, **z** may be a consequence of both the target and the data. In such a situation conditioning on it can create a form of selection bias, sometimes known as “collider bias” [64,65]. Conditioning on the third variable **z** can then reverse the correlation between 2 variables **X** and **y**, a phenomenon known as the Berkson or Simpson statistical paradox [66,67]. It can be understood from a simple example: when studying a population of hospital patients, individuals may have been admitted to the hospital because they have disease *A* or *B*. On this specific population, the 2 diseases are anti-correlated. However, concluding that disease *A* protects from disease *B* would be incorrect. Another example can be found in a cognitive experiment where both a visible-enough stimulus and a timely motor response are needed for a successful response. When learning a model decoding stimulus visibility from brain response, deconfounding on successful responses would lead this model to rely on motor cortex activity, while the link between visual stimulus and motor cortex is not neuroscientifically relevant as such. Deconfounding by itself does not suffice to yield associations with clear interpretations.

#### A sampling view on confounds

Confound-isolating cross-validation strives to sample an ideal subpopulation. This is also one of the best strategies to avoid the presence of confounds in experimental settings: targeting the recruitment of participants so that the design is balanced, e.g., with matched controls or randomized controlled trials. But this can only be done at study design, and targeted acquisitions, with matching and restriction, can make it hard to collect large samples or tackle many covariates. At analysis time, researchers have to rely on statistical methods to adapt the analysis to the presence of confounds. For in-sample analysis, propensity scores are a classic reweighting technique used to obtain reliable effect estimates from confounded datasets [68,69]. The use of subsampling in confound-isolating cross-validation can be seen as an extension of these ideas for out-sample validation of predictive accuracy. The only caveat is that one has to ensure that sampling does not deterministically lead to a fixed test set, which would weaken the statistical guarantees brought by the validation experiment. Here, we propose to perform this check a posteriori. In the future, more complex sampling strategies could be designed to ensure some randomness in the test set.

### Conclusion: deconfounding and isolating confounds are complementary

Deconfounding strives to remove confounding effects from the data, after which successful prediction can be interpreted as a direct link from the remaining brain signals to the outcome of interest. However, in biomarker settings, the primary focus may be on the quality of detection, rather than interpretation, e.g., to improve diagnosis or prognosis. In such settings, an important question is, how much do the brain signals improve the prediction upon a simpler measure of the confounding effect? Answering this question calls for a cross-validation procedure isolating this confounding effect. The corresponding prediction accuracy can then safely be interpreted as not resulting in any way from the confounding effect.

## Availability of Supporting Source Code and Requirements

- Project name: Confound Prediction
- Project home page: https://github.com/darya-chyzhyk/confound_prediction
- Operating systems: OS independent
- Programming language: Python
- Other requirements: Python (≥3.5), Scipy (≥1.1.0), Scikit-learn (≥0.21.2), Numpy (≥1.14.2), Pytest (≥5.1.1)
- License: BSD 3-Clause License

## Data Availability

Supporting data and Python implementation to control confound effect are available via the *GigaScience* database GigaDB [70].

## Abbreviations

BASC: Bootstrap Analysis of Stable Clusters; GLM: general linear model; KDE: kernel-density estimator.

## Competing Interests

The authors declare that they have no competing interests.

## Funding

This work was funded by the Child Mind Institute. Resting state MRI data analysis was done using the UK Biobank Resource under project 25163. B.T. was also supported by the SC1-DTH-07-2018 H2020 VirtualBrainCloud Project under grant agreement No. 826421, and G.V. by the DirtyData (ANR-17-CE23-0018-01) project.

## Authors' Contributions

DC carried out data preprocessing, DC, GV and BT planned the experiments. DC, GV and BT wrote the manuscript, advanced conception and design of the study, all authors contributed to the final version of the manuscript.

## Supplementary Material

giac014_GIGA-D-21-00125_Original_Submission

giac014_GIGA-D-21-00125_Revision_1

giac014_GIGA-D-21-00125_Revision_2

giac014_GIGA-D-21-00125_Revision_3

giac014_Response_to_Reviewer_Comments_Revision_1

giac014_Response_to_Reviewer_Comments_Revision_2

giac014_Response_to_Reviewer_Comments_Revision_3

giac014_Reviewer_1_Report_Original_Submission Lawrence Hall -- 6/14/2021 Reviewed

giac014_Reviewer_1_Report_Revision_1 Lawrence Hall -- 9/30/2021 Reviewed

giac014_Reviewer_2_Report_Original_Submission Qingyu Zhao -- 6/22/2021 Reviewed

giac014_Reviewer_2_Report_Revision_1 Qingyu Zhao -- 10/6/2021 Reviewed

giac014_Supplemental_Files

## Appendices

### Data preprocessing

CamCan data were preprocessed using Pypreprocess [71], a collection of Python scripts for preprocessing fMRI data, that is based on the SPM12 software and the nipype toolbox [72]. We preprocessed CamCan data only. For UKBB data the preprocessed and connectivity matrices are available from the data repository. We apply a commonly used protocol that includes the following steps: Motion correction, correction for subject’s head motion during the acquisition. Estimated six motion parameters (three translational parameters and three rotational parameters) are used as confounds in the age prediction experiments. For each subject we expressed the head motion using translation across all three axes as a square root of the mean of the sum of square finite difference of each translation axes over the time: $\sqrt{\dfrac{\vec{\Delta } translation_x ^2 + \vec{\Delta } translation_y ^2 + \vec{\Delta } translation_z ^2}{3}}$ The rest-fMRI data are coregistered to the anatomical T1-MRI and then normalized to MNI template.
