# Supplementary material for: How to remove or control confounds in predictive models, with applications to brain biomarkers
Source: Gigascience. 2022 Mar 12;11:giac014. doi: 10.1093/gigascience/giac014 (PMC8917515; doi:10.1093/gigascience/giac014)

GigaScience

How to remove or control confounds in predictive models, with applications to brain biomarkers.

--Manuscript Draft--

**Manuscript Number:**	GIGA-D-21-00125R3
**Full Title:**	How to remove or control confounds in predictive models, with applications to brain biomarkers.
**Article Type:**	Research
**Funding Information:**	Child Mind Institute — Dr. Darya Chyzhyk<br>VirtualBrainCloud Project (SC1-DTH-07-2018 H2020) — Dr Bertrand Thirion<br>DirtyData (ANR-17-CE23-0018-01) — Dr. Gaël Varoquaux
**Abstract:**	Background<br>With increasing data sizes and more easily available computational methods, neurosciences rely more and more on predictive modeling with machine learning, eg to extract biomarkers of pathologies. Yet, a successful prediction may capture a confounding effect correlated with the outcome instead of brain features specific to the outcome of interest –eg the pathology. For instance, as patients tend to move more in the scanner than controls, imaging biomarkers of a pathology may mostly reflect head motion, leading to inefficient use of resources and wrong interpretation of the biomarkers.<br>Resuts<br>Here we study how to adapt statistical methods that control for confounds to predictive modeling settings. We review how to train predictors that are not driven by such spurious effects. We also show how to measure the unbiased predictive accuracy of these biomarkers, based on a confounded dataset. For this purpose, cross-validation must be modified to account for the nuisance effect. To guide understanding and practical recommendations, we apply various strategies to assess predictive models in the presence of confounds on simulated data and population brain imaging settings. Theoretical and empirical studies show that deconfounding should not be applied to the train and test data jointly: modeling the effect of confounds, on the train data only, should instead be decoupled from removing confounds.<br>Conclusions<br>Cross-validation that isolates nuisance effects gives an additional piece of information: confound-free prediction accuracy.
**Corresponding Author:**	Darya Chyzhyk, PH.D<br>Inria Saclay: Inria Centre de Recherche Saclay-Ile-de-France<br>Palaiseau, FRANCE
**Corresponding Author Secondary Information:**	
**Corresponding Author's Institution:**	Inria Saclay: Inria Centre de Recherche Saclay-Ile-de-France
**Corresponding Author's Secondary Institution:**	
**First Author:**	Darya Chyzhyk, PH.D
**First Author Secondary Information:**	
**Order of Authors:**	Darya Chyzhyk, PH.D<br>Gaël Varoquaux<br>Michael Milham<br>Bertrand Thirion
**Order of Authors Secondary Information:**	

Powered by Editorial Manager® and ProduXion Manager® from Aries Systems Corporation

Response to Reviewers:	Dear Hans,<br><br>Thank you for proceeding so quickly.<br>We have made all asked corrections and our manuscript is revised and ready for publication.<br><br>Best regards,<br>Darya
Additional Information:	
Question	Response
Are you submitting this manuscript to a special series or article collection?	No
**Experimental design and statistics**<br><br>Full details of the experimental design and statistical methods used should be given in the Methods section, as detailed in our Minimum Standards Reporting Checklist. Information essential to interpreting the data presented should be made available in the figure legends.<br><br>Have you included all the information requested in your manuscript?	Yes
**Resources**<br><br>A description of all resources used, including antibodies, cell lines, animals and software tools, with enough information to allow them to be uniquely identified, should be included in the Methods section. Authors are strongly encouraged to cite Research Resource Identifiers (RRIDs) for antibodies, model organisms and tools, where possible.<br><br>Have you included the information requested as detailed in our Minimum Standards Reporting Checklist?	Yes
**Availability of data and materials**<br><br>All datasets and code on which the conclusions of the paper rely must be either included in your submission or	Yes

deposited in publicly available repositories (where available and ethically appropriate), referencing such data using a unique identifier in the references and in the “Availability of Data and Materials” section of your manuscript.<br><br>Have you have met the above requirement as detailed in our Minimum Standards Reporting Checklist?	

LaTeX - Main Document (TeX file)

Click here to access/download;LaTeX - Main Document (TeX file);GigaScience-2020.tex

OXFORD

(GIGA)^n SCIENCE

GigaScience, 2020, 1–13

doi: xx.xxxx/xxxx
Manuscript in Preparation
Paper

PAPER

How to remove or control confounds in predictive models, with applications to brain biomarkers

Darya Chyzhyk [1,2,3,*], Gaël Varoquaux [1,2], Michael Milham [3,4] and Bertrand Thirion [1,2]

[1]Parietal project-team, INRIA Saclay-île de France, France and [2]CEA/Neurospin bât 145, 91191 Gif-Sur-Yvette, France and [3]Center for the Developing Brain, Child Mind Institute, New York, New York 10022, USA and [4]Center for Biomedical Imaging and Neuromodulation, Nathan S. Kline Institute for Psychiatric Research, Orangeburg, New York 10962, USA

*darya.chyzhyk@gmail.com

Abstract

Background
With increasing data sizes and more easily available computational methods, neurosciences rely more and more on predictive modeling with machine learning, *eg* to extract biomarkers of pathologies. Yet, a successful prediction may capture a confounding effect correlated with the outcome instead of brain features specific to the outcome of interest –*eg* the pathology. For instance, as patients tend to move more in the scanner than controls, imaging biomarkers of a pathology may mostly reflect head motion, leading to inefficient use of resources and wrong interpretation of the biomarkers.
Resuts
Here we study how to adapt statistical methods that control for confounds to predictive modeling settings. We review how to train predictors that are not driven by such spurious effects. We also show how to measure the unbiased predictive accuracy of these biomarkers, based on a confounded dataset. For this purpose, cross-validation must be modified to account for the nuisance effect. To guide understanding and practical recommendations, we apply various strategies to assess predictive models in the presence of confounds on simulated data and population brain imaging settings. Theoretical and empirical studies show that deconfounding should not be applied to the train and test data jointly: *modeling* the effect of confounds, on the train data only, should instead be decoupled from *removing* confounds.
Conclusions
Cross-validation that isolates nuisance effects gives an additional piece of information: confound-free prediction accuracy.

Key words: confound, subsampling, phenotype, predictive models, biomarkers, statistical testing, deconfounding

Introduction

Predictive models, using machine learning, are becoming a standard tool for scientific inference. In cognitive neuroscience, they can be used for *decoding*, to make conclusions on mental processes given observed brain activity [1, 2, 3]. With the rise of large-scale brain-imaging cohorts, they can extract imaging biomarkers that predict across subjects phenotypes such as neuropsychiatric conditions [4, 5, 6] or individual traits [7, 8].

A crucial aspect of these biomarkers is their ability to *predict*

Compiled on: January 22, 2022.
Draft manuscript prepared by the author.

the outcome of interest, *ie* to generalize to new data [9]. However, these predictions can be driven by confounding effects. Such effects affect both the brain-imaging data and the prediction target but are considered as irrelevant. For instance, brain imaging reflects age quite accurately, and actually carries information about age-related diseases [8, 10, 11], yet [12] showed that subjects' in-scanner motion varies with subjects' age and it creates systematic differences in recorded brain imaging signals. Given this confounding effect, MRI biomarkers of brain aging may be nothing more than expensive measurements of head motion. Other examples may be more subtle: age matters for diagnosing Alzheimer's disease, yet an important question is whether brain imaging yields an accurate diagnosis of Alzheimer disease beyond the mere effect of age.

More generally, the data at hand often capture effects not of direct interest to the investigation. In many situations, some confounds such as head motion cannot be fully avoided. To make matters worse, large cohorts developed in population imaging to answer epidemiological questions [as UK biobank, 13] are observational data: there is no controlled intervention or balanced case-control group; rather, individuals are recruited from diverse populations with various sampling or selection biases. To conclude on the practical use of biomarkers, it is important to ensure that their predictions are not fully driven by such unwanted effects. This requires measuring model predictive accuracy after controlling for nuisance variables. Confounding effects can also make it hard to interpret brain-behavior relationships revealed by predictive models [14], as confounds can mediate the observed association or be a latent common cause of observations [15].

In experimental settings, *eg* as in a small cohort, confounding can be suppressed by balancing the acquisition for confounds, or using randomized control trials. However, constraints in the data acquisition, *eg* recruitment of a large cohort, often imply that confounds are present in the data, and appropriate analysis is needed to avoid reaching erroneous conclusions. The statistical literature on controlling confounding variables is well developed for classic statistical analysis, such as statistical testing in a linear model at the heart of the standard mass-univariate brain mapping [16, 17]. However, these procedures need to be adapted to high-dimensional predictive-modeling settings, where the focus is to achieve high-prediction accuracy based on imaging data. Indeed, predictive models do not rely on the same parametric assumptions, namely linearity of effects and Gaussian noise. Often, a predictive analysis does not build on a generative model of the signal but on optimizing discrimination [18]. In addition, predictive models draw their purpose and validity from out-of-sample prediction, rather than in-sample statistical testing [19]. The question tackled here is thus whether one can assess the predictive accuracy of brain measurements free of unwanted confounds. It is *not* to identify treatment effects size nor to perform other types of causal inference.

In this paper, we study statistical tools to control for confounding effects in predictive models. We consider that practitioners should primarily avoid or reduce the impact of confounds on their model, but this is not always feasible or maybe hard to check, hence, we choose to put the emphasis on the unbiased evaluation of models in the presence of confounds. A preliminary version of the work discussed here was presented at the PRNI conference [20]. While the core method is the same, it presents limited insights on the theoretical underpinnings and practical value of the method proposed. Experiments on simulated data are absent and experiments on neuroimaging data are limited to just one data set. In particular, statistical significance is not established thoroughly, and only one alternative approach is considered. In short the conference publication provides limited insights on the method, while the current work provides a complete description and points to the code for reuse.

We first review how the classic deconfounding procedures can be used in predictive-modeling settings, i.e. together with cross-validation. We then expose a complementary approach that is not based on removing confounding effects, but rather testing whether a given predictive model –*eg* a biomarker– predicts well when these confounds are not present. For this we introduce the *confound-isolating cross-validation* method, that consists in sampling test sets in which the effect of interest is independent from the confounding effect. The benefits of this approach are that it is non-parametric and that it directly tests the quantity of interest in a predictive analysis. We then run an extensive empirical study on three population-imaging biomarker extraction problems, a tabular dataset, as well as simulations. We draw practical recommendations to test predictive models in the presence of confounding effects.

Methods: controlling for confounds in predictive models

Formalizing the problem of prediction with a confound

Assessing predictive models

Predictive models are assessed by their prediction accuracy [19]. For this, cross-validation is the standard tool, typically k-fold cross-validation [21]. It consists in partitioning (potentially randomly) the original dataset into k equal size subsets or folds (each denoted by a color in Figure 1). One of these k sets is held out for testing, and the remaining $(k-1)$ folds are used for training the model. This process is repeated k times, where each time a different group of observations compose the test set. Prediction accuracy is measured on the test set, then averaged across folds.

Confounding variables in a prediction task

To formalize prediction in presence of a confound, we consider a dataset of n observations –*eg* subjects or time-points– comprising $p-$ *dimensional* brain signals $\mathbf{X} \in \mathbb{R}^{n \times p}$, an effect of interest[1] $\mathbf{y} \in \mathbb{R}^n$ –the biomarker target– and a confounding effect $\mathbf{z} \in \mathbb{R}^n$.

An imaging biomarker then predicts $\mathbf{y}$ from $\mathbf{X}$. If $\mathbf{X}$ and $\mathbf{z}$ on the one hand, $\mathbf{y}$ and $\mathbf{z}$ on the other hand, are not independent, the prediction of the target $\mathbf{y}$ might be affected or most accurately done by the confounding effect, $\mathbf{z}$. Such prediction may be misleading or useless. It can be misleading as it can be interpreted as a link between brain structures and $\mathbf{y}$ –*eg* fluid intelligence– while such a link only reflects the effect of $\mathbf{z}$ –*eg* age. It can be useless because brain imaging is likely much more costly to acquire than the phenotypic variable $\mathbf{z}$, hence it should be used only if it brings more diagnostic information. Moreover, this can be detrimental to accuracy: if a future dataset shows an altered relation between the confound and the features, prediction accuracy may be compromised.

A crucial problem for the validity of the biomarker is to measure whether it can predict $\mathbf{y}$ from $\mathbf{X}$ and not solely from $\mathbf{z}$. Prediction accuracy is ideally measured on an independent validation set, but most often, no large independent validation set is available and a cross-validation procedure, that iteratively separates train and test sets [21], is used. In [22] what cross-validation captures in the presence of a confounding variable is discussed. Though there can be many possible confounds

1 In classification settings, $\mathbf{y}$ does not take continuous values in $\mathbb{R}^n$, yet we use the most general notation to cover both classification and regression settings.

in brain imaging (see section 8), we focus below on simple settings, assuming that the main confounding factor has been isolated in one variable.

There are two points-of-view to controlling confounds in predictive models. One is to try and *remove* the effect of the confounding variables from the data, by regressing them out (deconfounding) or resampling the data to cancel spurious correlations (re-balancing). The other is to test that the model's prediction captures more than the confound. Removing the confounding signal can test whether predictions are fully driven by the confound $\mathbf{z}$ rather than the brain signal $\mathbf{X}$. However, it does not provide a good tool to measure the predictive power in the presence of confounds: the accuracy is likely biased, as illustrated later in the simulations.

Another point of view on confounding effects in predictive modeling consists in trying to learn a predictor from a biased population –with the confounding effect– that differs from the population of interest –without the confounding effect. The problem can then be tackled as a *domain adaptation* problem [23, 24]. However, [24] have shown that compensating for the confound does not improve prediction if the test population is not markedly different from the training population. Note that train and test samples are often drawn from the same population, either because only one cohort is available or because a proper stratification scheme is used. Our question is different: we are interested in assessing whether learning a biomarker on a confounded dataset leads to predictions that are fully driven by the confound.

Deconfounding

Deconfounding in standard analysis

In inferential statistics –as opposed to predictive modeling– proper modeling of confounds is important to control the *interpretation* of model parameters, ensuring that they are not driven by the confounding effects. Classical statistic analysis in brain imaging is based on the general linear model (GLM) [25, 16], in which confounding effects are controlled by additional regressors to capture the corresponding variance. Such an approach shows limitations in predictive-modeling settings. First, it is based on maximum-likelihood estimates of linear models, while in general, predictive models are not explicitly based on a likelihood and are often not linear. Second, it is designed to control *in-sample* properties, while predictive models are designed for *out-of-sample* prediction. The two-step approach based on applying a classical GLM to remove the confounding effect, then a predictive model, may lead to pessimistic results, *eg* below-chance prediction [8, 26].

In the context of the GLM, an alternative implementation relies on removing the effect of variables that are correlated. [25]. Note that in all this work we assume that the confounder is associated with $\mathbf{X}$ and $\mathbf{y}$ without creating three ways interactions between $\mathbf{X}$, $\mathbf{y}$ and $\mathbf{z}$. Given a sample $\mathbf{X} \in \mathbb{R}^{n \times p}$ of n observations (subjects) with p brain imaging features (*eg* connectivity matrices), $\mathbf{X}_i = (\mathbf{X}_{i1}, \mathbf{X}_{i2}, \ldots, \mathbf{X}_{ip})$ and confounds $\mathbf{z} \in \mathbb{R}^n$, the model is:

$$\mathbf{X} = \mathbf{z}^{\mathbf{T}}\mathbf{w} + \mathbf{e}, \qquad (1)$$

where $\mathbf{w}$ is a vector of weights (one per voxel, $\mathbf{w} \in \mathbb{R}^p$). $\hat{\mathbf{w}}$ represents the estimated coefficients, that are obtained typically through least-squares regression:

$$\hat{\mathbf{w}} = (\mathbf{z}^{\mathbf{T}}\mathbf{z})^{-1}\mathbf{z}^{\mathbf{T}}\mathbf{X} \qquad (2)$$

Given these equations, a linear model can be used prior to the predictive model to remove the effect of the confounds $\mathbf{z}$ on the brain signals $\mathbf{X}$. It must be adapted to out-of-sample testing. One solution is to apply deconfounding jointly on the train and the test set, but it breaks the statistical validity of cross-validation because it couples the train and the test set [21]. Hence it can give biased results.

Out-of-sample deconfounding

To adapt the above *deconfounding* approach to the two phases of training and testing a predictive model, a useful view is to consider the deconfounding model as a predictive encoding model, predicting a fraction of the signal $\mathbf{X}$ from $\mathbf{z}$. Deconfounding is then performed by removing the part of the signal captured by $\mathbf{z}$ from $\mathbf{X}$:

$$\hat{\mathbf{X}}_{\mathbf{clean}} = \mathbf{X} - \mathbf{z}\hat{\mathbf{w}} \qquad (3)$$

Where $\hat{\mathbf{w}}$ are the coefficients of the linear deconfounding model (Equation 1), estimated on the train data with Equation 2 and then applied to the test [26]. The full out-of-sample deconfounding procedure is listed in algorithm 1.

A drawback of such deconfounding is that it is strongly parametric, i.e. it relies on the model of confounds used. Equation 2 stands for the classic linear model, assuming linearity between the confounding variable $\mathbf{z}$ and the brain signal $\mathbf{X}$. The linear model only takes into account second-order statistics (covariance or correlations) and ignores more complex dependencies.

Model-agnostic out-of-sample deconfounding

A common solution to go beyond linear effects of confounds is to use a polynomial expansion of the confounds $\mathbf{z}$ in the linear deconfounding model. Another option is to use a more powerful predictive model in the confound removal. A predictive model –including a mere linear model– regressing $\mathbf{X}$ on $\mathbf{z}$ can be seen as estimating a function f so that $f(\mathbf{z}) = \mathbb{E}[\mathbf{X}|\mathbf{z}]$. There are many possibilities such as random forests or Gaussian processes. The procedure used for out-of-sample deconfounding can then be adapted as in Algorithm 2. While this approach is very powerful, the danger is to remove also part of the signal of interest. Indeed, using a more powerful predictive model, for instance a higher-order polynomial, leads to explaining in $\mathbf{X}$ more data as a function of $\mathbf{z}$; however too powerful models *overfit*, which means that they explain variance in $\mathbf{X}$ by chance. In such a situation, the deconfounding procedure may remove signal of interest, unrelated to the confound.

Comparing predictive power of confounds

A simple evaluation of the impact of $\mathbf{z}$ on the prediction of $\mathbf{y}$ is to use predictive models predicting $\mathbf{y}$ from $\mathbf{z}$ (*prediction from confound*) and compare the predictive accuracy to that obtained with biomarkers based on brain signals. This argument is used in [6] to control for the effect of movement on autism diagnostic.

Algorithm 1: Out-of-sample deconfounding

Input: Brain signal $\mathbf{X} \in \mathbb{R}^{n \times p}$, confound $\mathbf{z} \in \mathbb{R}^n$, {train} and {test} indices

$\hat{\mathbf{w}}_{\mathbf{confounds}} \leftarrow (\mathbf{z}^{\mathbf{T}}{}_{\mathbf{train}}\mathbf{z}_{\mathbf{train}})^{-1}\mathbf{z}^{\mathbf{T}}{}_{\mathbf{train}}\mathbf{X}_{\mathbf{train}}$

```
/* Regression of confounds on data */
```

$\hat{\mathbf{X}}_{\mathbf{clean,test}} \leftarrow \mathbf{X}_{\mathbf{test}} - \mathbf{z}_{\mathbf{test}}\hat{\mathbf{w}}_{\mathbf{confounds}}$

```
/* Remove confounds in the test set */
```

Output: Brain signal without confounds $\hat{\mathbf{X}}_{\mathbf{clean,test}}$

Algorithm 2: Model-agnostic deconfounding. Note that f only has one argument, as it is a function that predicts $\mathbf{X}$ from $\mathbf{z}$, while g has two arguments (the input $\mathbf{X}$ and the output $\mathbf{z}$), as it represents the learning algorithm that yields f.

Input: Brain signal $\mathbf{X} \in \mathbb{R}^{n \times p}$, confound $\mathbf{z} \in \mathbb{R}^n$, {train} and {test} indices, machine-learning algorithm g

$f \leftarrow g(\mathbf{z}_{\text{train}}, \mathbf{X}_{\text{train}})$
 /* Fit confound model capturing $\mathbb{E}[\mathbf{X}|\mathbf{z}]$ */

$\tilde{\mathbf{X}}_{\text{clean,test}} \leftarrow \mathbf{X}_{\text{test}} - f(\mathbf{z}_{\text{test}})$
 /* Remove confounds in the test set */

Output: Brain signal without confounds $\tilde{\mathbf{X}}_{\text{clean,test}}$

Figure 1. Classic and confound-isolating cross-validation. a) k-fold cross-validation is the common procedure to evaluate predictive models. It consists in splitting the data into k equal groups. k-1 folds are used to fit a model and 1 fold is used to validate the model. This process is repeated k times so that each sample is taken once in the test set. b) In *confound-isolating cross-validation* sampling we divide the data in train and test sets, but in a different way. First, using subsampling, we create a test set on which $\mathbf{y}$ and $\mathbf{z}$ are independent. The train test is constructed from the rest of the samples that are not included in the test set. In this way, the method creates a test set that contains unrelated target and confound.

Creating a test set to isolate the confounding effect

Rather than deconfounding, the investigator may ensure that the predictive model is useful by measuring its accuracy on a dataset where the confounding effect is absent. In a cross-validation setting, such a situation can be created by using as a test set a well-chosen subset of the data that isolates the confounding effect. See Figure 1 for a graphical illustration of the approach. Formally, it requires choosing a subset $\mathcal{S}$ of the data such that $\mathbf{y}_\mathcal{S}$ and $\mathbf{z}_\mathcal{S}$ are independent (the feasibility of this subset creation is discussed below).

The remainder of the data is used as a training set, to learn to predict $\mathbf{y}$ from $\mathbf{X}$. If the prediction generalizes to the test set $\mathcal{S}$, the learned relationship between $\mathbf{X}$ and $\mathbf{y}$ is not entirely mediated by $\mathbf{z}$. In particular, the prediction accuracy then measures the gain in prediction brought by $\mathbf{X}$.

Categorical confound

The confounding effect can be "categorical", for instance the site effect when learning predictive biomarkers on multi-site acquisitions as in [6]. In such settings, to test that the model can indeed predict independently from site effects, a simple solution is to resort to a cross-validation that avoids having samples from the same site both in the train and the test sets. This may imply resampling the data to cancel out associations between site and target related to data imbalance. Similarly, in multi-subject prediction with repeated measurements from the same subject, subject-wise cross-validation can rule out that prediction is driven by subject identification [27, 22]. More generally, for a categorical confound $\mathbf{z}$, having distinct values for $\mathbf{z}$ in the train and the test set ensures that the prediction cannot be driven by $\mathbf{z}$. We note that this procedure is different from the stratification strategy used in classical statistics, but it clearly avoids any bias due to imperfectly corrected association between $\mathbf{z}$ and the other variables. In the case of site-related confounds, prediction accuracy will obviously suffer. This can be addressed with techniques such as invariant risk minimization [28], but we do not further consider this approach here.

Continuous confound

When $\mathbf{z}$ is a continuous variable, such as age, it is more challenging to generate test sets on which $\mathbf{y}_\mathcal{S}$ and $\mathbf{z}_\mathcal{S}$ are independent. We describe here an algorithm to generate such sampling, "confound-isolating cross-validation" subsampling. It is based on iterative sampling to match a desired distribution: the goal is to have a test set with independence between $\mathbf{y}$ and $\mathbf{z}$, i.e. $p(\mathbf{y}, \mathbf{z}) = p(\mathbf{y})\,p(\mathbf{z})$, where $p((\mathbf{y}, \mathbf{z}))$ is the joint probability function of $\mathbf{y}$ and $\mathbf{z}$, and $p(\mathbf{y})$ and $p(\mathbf{z})$ are the marginal probability distribution.

A related quantity is mutual information, which characterizes the level of dependency between the two variables: $\eta(\mathbf{y}, \mathbf{z}) = \mathbb{E}\left[\log\left(\frac{p((\mathbf{y},\mathbf{z}))}{p(\mathbf{y})p(\mathbf{z})}\right)\right]$. In practice we estimate the probability density functions with a kernel-density estimator (KDE) using Gaussian kernels. We iteratively create the test $\mathcal{S}$ set by removing subjects; at each iteration, we consider the problem as a distribution matching problem, matching $p(\mathbf{y}_\mathcal{S}, \mathbf{z}_\mathcal{S})$ and $p(\mathbf{y}_\mathcal{S})\,p(\mathbf{z}_\mathcal{S})$. For this, we use importance sampling: we draw randomly 4 subjects to discard with a probability $\frac{p(\mathbf{y}_\mathcal{S},\mathbf{z}_\mathcal{S})}{p(\mathbf{y}_\mathcal{S})\,p(\mathbf{z}_\mathcal{S})}$ using inverse sampling method [30, sec 2.2]. Algorithm 3 gives the details. The choice of 4 samples is tailored to the sample size considered here: it makes the algorithm faster than using one sample, yet is low enough not to compromise mutual information minimization. A Python implementation is available on GitHub [31] and on PyPI repository [32] and can be installed with `pip install confound-prediction`.

Note that if $\mathbf{z}$ and $\mathbf{y}$ are too strongly related, the subsampling procedure above does not have enough degrees of freedom and may always chose the same subset: the test set would be deterministically defined by the sampling procedures, in which case there would effectively be only one fold of cross-validation. In practice, it is important to check that such a situation does not occur when analyzing a given dataset. One way is to compute the average fraction of common samples between two tests sets created with different seeds. As this value ranges from 0 to 1, where 1 means that all test sets contain the same samples and 0 that test sets have no sample in common, it is important to check that it is low enough.

Empirical study methodology

We now describe the experimental materials underlying our empirical study of confound-controlling approaches in predictive models.

Simulation studies

To understand the behavior of the different accuracy scores, we present experiments on simulated data. We simulate a data set

Algorithm 3: Confound-isolating cross-validation

Input: Target $\mathbf{y} \in \mathbb{R}^n$, confound $\mathbf{z} \in \mathbb{R}^n$, size $m < n$

$\mathcal{S} \leftarrow \{1 \ldots n\}$ /* Initialize */
while $card(\mathcal{S}) > m$ **do**
  $p_y \leftarrow \text{KDE}(\mathbf{y}_\mathcal{S})$ /* Density estimation */
  $p_z \leftarrow \text{KDE}(\mathbf{z}_\mathcal{S})$
  $p_{(y,z)} \leftarrow \text{KDE}((\mathbf{z}_\mathcal{S}, \mathbf{y}_\mathcal{S}))$
  $\mathbf{m}_i \leftarrow \frac{p_{(y,z)}((\mathbf{z}_i,\mathbf{y}_i))}{p_y(\mathbf{y}_i)p_z(\mathbf{z}_i)}, \ \forall i \in \mathcal{S}$
  $\mathcal{S} \leftarrow \mathcal{S} - \{j\}$ Draw one index j to remove from S with probability $\mathbf{m}_j$ using inversion sampling [29].
end

Output: Set of test indices $\mathcal{S}$

$\mathbf{X_0} \sim \mathcal{N}(0, 1)$ with confound $\mathbf{z_0} \sim \mathcal{N}(0, 1)$ to predict continuous variable $\mathbf{y} \sim \mathcal{N}(0, 1)$. We evaluate two samples sizes: $n = 100$ and $n = 1000$. We use $p = 100$ features in $\mathbf{X_0}$. We study 3 scenarios:

- **No direct link between target and brain** where the brain signal does not provide any direct information to predict $\mathbf{y}$, but is observed with a confound linked to $\mathbf{y}$:

$$\text{observed confound } \mathbf{z} = \mathbf{y} + \mathbf{z_0},$$
$$\text{observed signal } \mathbf{X} = \mathbf{X_0} + \mathbf{z}.$$

- **Direct link between target and brain** where the brain signal does indeed provide information to predict $\mathbf{y}$ and has an additional confound linked to $\mathbf{y}$:

$$\text{observed confound } \mathbf{z} = \mathbf{y} + \mathbf{z_0},$$
$$\text{observed signal } \mathbf{X} = \mathbf{X_0} + \mathbf{y} + \mathbf{z}.$$

- **Weak confound & direct link between target and brain**

$$\text{observed confound } \mathbf{z} = 0.5\,\mathbf{y} + \mathbf{z_0},$$
$$\text{observed signal } \mathbf{X} = \mathbf{X_0} + \mathbf{y} + \mathbf{z_0}.$$

Note that one could consider instead a canonical scheme in which $\mathbf{z}$ would cause $\mathbf{x}$ and $\mathbf{y}$. Since our work is not on causal inference *per se*, we aim at a statistical procedure that does not require a prescribed causal relationship between the variables, which is often unknown.

Two classic confounded predictions in population imaging

Motion confounding brain-age prediction

As brain aging is a risk factor of many pathologies, the prediction of brain age from MRI is a promising biomarker [11]. In childhood also, markers of functional brain development can help to recognize neurodevelopmental disorders [33, 34]. Many recent studies report age prediction, *eg* from resting-state functional connectivity [7, 33, 35], from structural imaging [36], or combining multiple imaging modalities [8, 10]. However, older people and children move more in the scanner than young adults [see fig. 2, 37, 38, 12, 39]. Thus, age-related changes observed in brain images may be confounded by head motion [40] and image quality [41].

Indeed, in-scanner motion creates complex MRI artifacts that are difficult to remove [40]. In addition, they severely impact measurements of functional connectivity [42].

Here the confounding effect is that of head motion during the few hundreds of scans of individual acquisitions. To build a variable summarizing head motion for each subject, we use the movement time-series computed during preprocessing. As suggested in [42], we create the confound $\mathbf{z}$ from the root mean squared displacements (position differences across consecutive time points) for each subject $z = \sqrt{\frac{1}{I-1}\sum_{i=2}^{I}\left((t_x^i - t_x^{i-1})^2 + (t_y^i - t_y^{i-1})^2 + (t_z^i - t_z^{i-1})^2\right)}$, where t_x is left/right, t_y anterior/posterior, and t_z - superior/inferior translation and $i \in [\![I]\!]$ is the time index. The prediction target $\mathbf{y}$ is the age in years.

Age confounding fluid-intelligence measures

Various studies have predicted individual cognitive abilities from brain functional connectivity [43, 44]. In particular, [44] used machine-learning to predict fluid intelligence from rest fMRI. Fluid Intelligence quantifies the ability to solve novel problems independently from accumulated knowledge, as opposed to crystallized intelligence that involves experience and previous knowledge [45]. It is well known that cognitive abilities change with age [46, 47, 48, 49], in particular that fluid Intelligence progressively declines in middle age [50], while crystallized intelligence continues to grow with age. Indeed, in a cohort with a large age span, the data display a strong relation between fluid intelligence and age (Figure 2). When extracting biomarkers of fluid intelligence, the danger is therefore to simply measure age. We study how to control the impact of age when predicting a fluid-intelligence score from rest-fMRI functional connectivity.

Population-imaging rest-fMRI datasets

Datasets

We ran experiments on 626 participants from the CamCan data set and 9 302 participants from UKBB. All participants are healthy subjects with no neurological disorders.

- **CamCan** Cambridge Center for Ageing and Neuroscience data [51] studies age-related changes in cognition and brain anatomy and function. Characteristics of interest of this dataset are *i)* a population lifespan of 18-88 years, *ii)* a large pool (626 subjects) of multi-modal MRI data and neurocognitive phenotypes.
- **UKBB** The UK Biobank project [52] is a prospective epidemiological study to understand the development of diseases of UK population over the years. The data used here contains 9 302 subjects from the first release of UK Biobank ongoing cohort study with available resting-state fMRI scans and extensive health and lifestyle information [53, 54].

Table 1 presents detailed information about the number of subjects and the scale of the scores for each data set.

We give detailed information on pre-processing steps for each dataset in Appendix 8, following COBIDAS recommendations [55].

Prediction from functional connectivity

To build predictive models from resting-state fMRI, we follow the recommendations in [56]. We use the BASC functional atlas [57] with 64 regions, based on which we extract fMRI time series from the CamCAN dataset. Next, we normalize, detrend and bandpass-filter between 0.01 and 0.1Hz the signal. We represent connectivity matrices with tangent parametrization [58]. Finally, we use a ridge regression with nested cross-validation to learn predictive biomarkers from the functional-connectivity matrices. We use Nilearn [59] for the whole predictive pipeline.

Tabular (non-imaging) data

The considerations on confounds in predictive models are not specific to imaging data. We also study a confounded prediction without brain signals: on the UKBB data, we consider predicting an individual's income from socio-demographics and mental-health assessments. We investigate education as a potential confound: it may be reflected both in mental-health

Table 1. Characteristics of the data used. The scores for Fluid Intelligence differ on the two datasets: CamCan uses the Cattell test, and UKBB a specifically-designed touch-screen questionnaire.

Dataset Information	**CamCan**	**UKBB**
Number of subjects	626	9 302
Age	18 – 88	40 – 70
Fluid Intelligence scale	Cattell (11 – 44 scores)	UKBB-designed (1 – 13 scores)

and in income. There are 8 556 individuals with no missing values on the outcome and confound. We use random forests for prediction, as it is a popular learner that is well suited to the distribution of these tabular data, that is often non-Gaussian and consists of categorical variables.

Experimental paradigm: cross-validation measures

We use cross-validation to assess prediction accuracy. We consider five predictive frameworks: (1) Without deconfounding, (2) Deconfounding test and train sets jointly, (3) Out-of-sampling deconfounding, (4) Confound-isolating cross-validation, (5) Prediction from confounds only. The code for these various strategy to control for confounds can be found on GitHub [31] and on PyPI repository [32] and can be installed with `pip install confound-prediction`.

We use 10 folds, with random splits of 20% of the data in the test set. For *confound-isolating cross-validation*, different seeds in the random number generator lead to different folds. We assess the null distribution of predictions with permutations (20 000 folds on permuted labels **y**).

Results of the empirical study

Simulated data

We first consider simulated data, for which there is a ground truth. Figure 3 shows the results of the different methods to control for confounds on 3 different simulated cases (Figure 9 gives results for the same simulations with 1 000 samples).

(a) In the case where there is no direct relationship between the data and the target, the performance of the prediction model should not be better than chance after controlling for the confound. Both joint deconfounding and *confound-isolating cross-validation* clearly reveal that all the prediction is mediated by the confound. Out-of-sample deconfounding displays a less clear signal, as there seems to be a slight prediction even after deconfounding, though it is not significant.

(b) For a direct link between the data and the target, joint deconfounding yields a false negative, in the sense that it fully removes the prediction from the brain signal: it is too aggressive in removing signal. Other approaches correctly support a successful prediction.

(c) For a weaker confounding signal, results are similar, however it is worth noting that the target can no longer be well predicted from the confound.

Overall, on the simulations, both *out-of-sample deconfounding* and *confound-isolating cross-validation* give reliable answers, while deconfounding the test and train jointly as well as measuring the prediction from confounds cannot be trusted.

Experiments on resting-state fMRI data

Potential confounds

Figure 2 shows the relationships between target variable y and confounds z. Fluid Intelligence (target) is strongly negatively correlated with age (confound) on the CamCan dataset (second column of Figure 2). Also, on the CamCan data, Age and Motion are very correlated (first column of Figure 2). On the more homogeneous and larger UKBB sample (9 302 subjects), this link is weaker.

Figure 2. Joint distribution of target and confound. The first column presents the scatter plot of age and motion variable for CamCan (top) and UKBB (bottom). The second column shows the case of fluid intelligence prediction with age as confound for CamCan. In all cases, the target is clearly associated with the confound; the corresponding p-values are below 10^{-5}.

Table 2. Comparisons on population-imaging data, Camcan Fluid Intelligence prediction.

Method	MAE $\pm\sigma$	MAE $\pm\sigma$ permuted	p-value
CamCan: Age prediction			
Without deconfounding	6.17 ± 0.43	16.0 ± 1.24	0
Deconfounding test and train jointly	6.76 ± 0.53	16.24 ± 0.66	0
Out-of-sample deconfounding	9.3 ± 0.8	15.79 ± 1.22	0
Confound-isolating cross-validation	6.49 ± 0.46	15.21 ± 1.37	0
Prediction from confounds	13.74 ± 1.5	15.22 ± 1.74	0
CamCan: Fluid Intelligence prediction			
Without deconfounding	4.1 ± 0.29	6.05 ± 0.49	0
Deconfounding test and train jointly	4.08 ± 0.22	5.7 ± 0.34	0
Out-of-sample deconfounding	5.59 ± 0.32	6.04 ± 0.9	0.23
Confound-isolating cross-validation	4.31 ± 0.29	4.6 ± 0.3	0.06
Prediction from confounds	4.03 ± 0.6	5.7 ± 0.95	0
UKBB: Age prediction			
Without deconfounding	4.82 ± 0.4	6.95 ± 0.8	0
Deconfounding test and train jointly	4.95 ± 0.4	6.92 ± 0.8	0
Out-of-sample deconfounding	8.23 ± 0.33	7.12 ± 0.34	1
Confound-isolating cross-validation	5.08 ± 0.3	7.26 ± 0.6	0
Prediction from confounds	6.24 ± 0.73	6.29 ± 0.72	1
Tabular data: Income prediction			
Without deconfounding	0.79 ± 0.014	0.93 ± 0.016	0
Deconfounding test and train jointly	0.79 ± 0.014	0.93 ± 0.016	0
Out-of-sample deconfounding	0.77 ± 0.014	0.93 ± 0.016	0
Confound-isolating cross-validation	0.85 ± 0.13	0.94 ± 0.18	1
Prediction from confounds	0.87 ± 0.016	0.93 ± 0.016	0

Confound-isolating cross-validation

Figure 4 displays the evolution of the association between confound and target during *Confound-isolating cross-validation* in the CamCan dataset, predicting Fluid Intelligence with Age as a confound. In the full dataset, comprising 608 subjects, the correlation between confound and target is $\rho = -0.67$. Iterating the algorithm to remove half of the subjects leads to $\rho = -0.17$. The final test set contains 1/5 of the initial set of subjects and achieves $\rho = -0.07$, showing that it indeed cancels the dependency between aging and motion. The joint distribution between target and confound displayed in Figure 4 shows that the initial statistical dependency between these

Figure 3. Comparisons on simulated data. The left column of each sub-figure reports the prediction performance as the mean absolute error for the five approaches considered: Prediction from the data without deconfounding, prediction after deconfounding test and train jointly, prediction with out-of-sample deconfounding, prediction with confound-isolating cross-validation, and prediction from confounds. The left column displays the distribution across validation folds for the actual data (top, orange), and for permuted data distribution (bottom, gray). The right column displays the distribution of p-values across folds, obtained by permutation, and the text yields the aggregated p-value across folds, testing whether prediction accuracy is better than chance. Test subsets always represent 1/5 of the whole dataset. There are three simulation settings: (a) No direct link between target and brain, (b) A direct link between target and brain in the presence of a confound and (c) A weak confound and a direct link between target and brain. Green ticks indicate correct conclusions, red crosses mark incorrect ones, and warning signs the weak results.

Figure 4. Evolution of the test set created by Confound-isolating cross-validation. The joint distribution of the target (Fluid intelligence) and the confound (Age) for the CamCan dataset is taken for demonstration. We show the process of selecting proper samples for the test set. We begin with the entire dataset, Pearson correlation is −0.67 with infinitesimal p-value (right subplot). After half of the iterations we have already reached a correlation −0.17 with p-value = 0.009 (middle subplot). The final test set is shown on the right subplot, correlation −0.007 with p-value = 0.02. It presents negligible residual dependency between targets and confounds.

Figure 5. Evolution of the link between confound and target with the number of subjects for different subsampling methods on the CamCan dataset, considering age prediction. Applying Algorithm 3 effectively reduces statistical dependences between confound and target (red curve). In our experiments, we stop the sampling when the test set size is 1/5 of the dataset.

two variables vanishes after a few tens of iterations of the algorithm. Quantitative evaluation, measuring both Pearson correlation and mutual information (Figure 5) confirms that the confound-isolating procedure efficiently creates a subset of the data without the dependency as soon as it reduces the data to 300 subjects or less. Figure 8 shows similar success on the other prediction problems that we study.

In a cross-validation setting, the different test sets should probe different subjects to maximize testing power. A risk, when using *confound-isolating cross-validation*, is that it could repeatedly generate test sets with the same samples. To measure the diversity of the test sets, we compute the average fraction of common samples between two tests sets created with different seeds. The value is in the range from 0 to 1, where 1 means that all test sets contain the same samples and 0 that test sets have no sample in common; the expected value is $\frac{1}{5}$. We find an average intersection of 0.30 for age prediction with CamCan and 0.27 with UKBB; for Fluid Intelligence prediction with CamCan, we find 0.36. This demonstrates that the test sets do not repeat much, hence that there is no hidden determinism in the cross-validation scheme of the proposed method.

Testing for confounded prediction

Figure 6 and Table 2 report the mean absolute error[2] for the different approaches to control for confounds. The figure also reports the p-value of predictive accuracy, from permutations[3]. The first thing to note is that without controlling for confounding effects, all models lead to significant prediction. But are these driven by the confounds? Given that the various approaches measure predictions on different data, we compare how far these predictions are above chance, rather than their absolute value.

Deconfounding test and train sets jointly –removing the linear effect of the confounding variable on the full data– has little impact on the prediction performance on all datasets. On the other hand, *out-of-sample deconfounding* significantly changes prediction performance in a way that varies across tasks. Prediction accuracy of fluid intelligence on CamCan falls to chance level. Age prediction on CamCan is little impacted. However, Age prediction on UKBB gives results worse than chance, i.e. worse than a model that learns to predict age on data where this relationship has been shuffled by permutation (see Figure 6 and Table 2). *Confound-isolating cross-validation* also gives varying results on different datasets. For fluid-intelligence prediction on CamCan, it also gives results at chance level. For age prediction on CamCan, it does alter significantly prediction accuracy, and on UKBB, it leads to a slightly worse prediction, but still above chance. Finally, *Prediction from confounds* leads to chance-level or good prediction of the target depending on the dataset. In particular, it does better than chance for Fluid intelligence prediction.

These results show that in all these datasets, the confounds **z** are associated with both the data **X** and the target **y**. For fluid intelligence prediction on CamCan, all the prediction of **y** from **X** is mediated by **z**. However, for age prediction in CamCan, there exists within **X** some signal that is unrelated to **z** but predicts **y**. Age prediction in UKBB is a more subtle situation: **X** contains signals from **z** and **y** with shared variance, but there is enough signal beyond the effect of **z** to achieve a good prediction, as demonstrated by *confound-isolating cross-validation*, where the prediction cannot be driven by **z**. Yet, out-of-sample deconfounding removes the shared variance and hence creates predictions that are worse than chance.

Tabular data

Figure 7 and Table 2 give the results of analysis on the tabular data. There is a significant prediction of income from socio-demographic and mental-health information, without any deconfounding. However, prediction from confounds shows that qualifications also predict income well. To control for qualification, deconfounding removes the signal explained by these in **X**. Here, deconfounding does not make the prediction worse; actually out-of-sample deconfounding improves it. Such an improvement can be explained if the deconfounding adds information about the confound to the signal rather than removing it, as can happen when the model of the confounds is mis-specified. To limit mis-specification issues, a random forest as the g function in algorithm 2. Finally, confound-isolating cross-validation shows very variable results, but overall that prediction does not work better than chance on balanced datasets, so that qualification is no longer specifically related to income.

Here, deconfounding leads to the conclusion that the prediction of income from social-demographic and mental-health information is not at all driven by qualifications while the other approaches suggest otherwise. The discrepancy is probably due to the complex non-linear interactions between these variables. The reality is probably that qualifications contribute to the prediction of income, as well as mental health and socio-demographics information, and that teasing out these contributions is hard.

2 Mean absolute error is a good metric to compare across different test sets as it gives an absolute error measure in the unit of **y**, unlike explained variance, that depends on the variance of **y**.

3 Technically, there is one p-value per fold; to report only one number, we use p-value aggregation [60].

Discussion and conclusion

Measuring the accuracy of predictive models, *eg* for biomarkers or brain decoding, must account for the presence of confounding effects that can contribute to the prediction. Indeed, an imaging biomarker that solely picks up head motion may detect pathologies with some success, but be overall a waste of scanner time. An accurate prediction of fluid intelligence from brain functional connectivity might simply be a consequence of indirectly capturing the subjects' age. Standard cross-validation procedures ignoring the confounds can overestimate prediction accuracy.

Addressing confounds in predictive modeling

Approaches must be adapted to out-of-sample settings

Deconfounding approaches used in standard GLM-based analysis must be adapted to out-of-sample data by separating estimation of the confounds' model from removal of the effect of confounds on the data, as detailed in section and algorithm 1. Importantly, applying deconfounding to the whole data without separating train and test set is not only wrong in theory –because it breaks independence of train and test data– but also leads to incorrect conclusions in practice, as clearly visible from the simulations.

Even done right, deconfounding in predictive settings can lead to pessimistic evaluations, as stressed by [26] and shown in our experiments. This is because the signal explained by the confound is removed from the brain signal before it is passed to the predictive model. The corresponding correction can remove too much information when there a large amount of shared signal between the confound and the target –eg aging and Alzheimer's disease. Such problem does not arise in a GLM-based standard analysis because the confounds and the effects of interest are modeled *simultaneously*, and the consequences of shared signal are easier to handle.

To give a measure of predictive accuracy that is not pessimistic, we also study a different approach: testing the predictive model on a subset of the data crafted such that the effect of interest is independent from the confound. When the confounding effect is represented as a categorical variable, for instance the effect of acquisition site, the approach can be simple as it amounts to splitting the data so as to ensure that generalization occurs for a category not observed in the training set. Creating an adequate test set for continuous confounds requires a dedicated method, as with *confound-isolating cross-validation* (Algorithm 3). It enables a test of the predictive power from brain imaging without discarding the potentially useful shared signal. In addition, it is non-parametric and does not rely on a linear confounding model. Empirical studies, on both brain-imaging data and simulations, show that both out-of-sample deconfounding and *confound-isolating cross-validation* can control correctly for confounds. Deconfounding before fitting a predictive model brings the benefit of building a predictor free of the confounding effect. However, it can remove shared variance and lead to pessimistic evaluations. *Confound-isolating cross-validation* brings the benefit of measuring the predictive power in the absence of the confounding effect. Such measure is of direct importance to gauge the practical value of a biomarker. As an attractive complementary approach, note that deep learning approaches for learning confound-free models have been proposed in [61].

To summarize, our main claim is that it is possible to learn a confounded model yet evaluate it in an unbiased fashion. What matters in this logic is that the predictive accuracy after *confound-isolating cross-validation* remains better than chance, which amounts to performing an omnibus test of the variables of the model. The case where *confound-isolating cross-validation* would yield a null result certainly means that one should be cautious in claiming a conditional association between X and y, as slight variations in the confounding model may render the association significant or not: indeed the apparent association between features and target is dominated by the confounder and thus, not a reliable one. In brief, this has an impact on the practical significance of claimed associations.

Which approach to use when: deconfounding versus confound-isolating cross-validation

Out-sample deconfounding and *confound-isolating cross-validation* give valid and complementary information. In the worst case, these approaches can be conservative, but they don't yield spurious associations. From a prediction perspective, when the training population reflects adequately the target population, changing the training data to remove the effect of the confounder may not improve prediction accuracy [24]. For instance, for many pathologies, patients move more in the scanner than healthy individuals. Should an imaging-biomarker of the pathology be developed, this effect will be most likely true in the population on which the biomarker is applied. Hence it is counter-productive to force the biomarker to discard this information. Rather, *confound-isolating cross-validation* should be used to check that the imaging biomarker does bring in value in addition to capturing motion.

On the other hand, *confound-isolating cross-validation* is not a universal remedy: removing a confounding effect from the training data may be useful if the confounder is incidentally correlated with $\mathbf{X}$ or $\mathbf{y}$ without any clear causal relationship. This is the case if the confounder is a feature of the measurement process. For instance, if the data are acquired across two imaging sites with different scanners, but one site recruited a much larger fraction of patients than the other, the risk is that the predictor learns to use information about the scanner rather than the pathology. In such a case, the training strategy must be adapted, for instance by removing the effect of the confound.

Finally, if the goal is to *interpret* successful prediction as evidence of a link between brain signals and the predicted outcome, modifying the training data is more likely to disentangle the biomarker pattern of interest from the confounding effect. In such a situation, deconfounding should be preferred, to give a model, with its parameters, that is not driven by the confounding signal.

Figure 6. Comparisons on population-imaging data Each sub-figure shows one prediction setting: (a) CamCan Age prediction, (b) CamCan Fluid Intelligence prediction, (c) UKBB Age prediction. The left column of each sub-figure reports the prediction performance as the mean absolute error for the five approaches considered: Prediction from the data without deconfounding, prediction after deconfounding test and train jointly, prediction with out-of-sample deconfounding, prediction with confound-isolating cross-validation, and prediction from confounds. The left column displays the distribution across validation folds for the actual data (top, orange), and for permuted data distribution (bottom, gray). The right column displays the distribution of p-values across folds, obtained by permutation, and the text yields the aggregated p-value across folds (see the main text), testing whether prediction accuracy is better than chance. Test subsets always represent 1/5 of the whole dataset. The figure clearly displays different behaviors across the three problems: without deconfounding, and deconfounding test and train jointly yield significant, but probably spurious accuracy; out-of-sample deconfounding can be over-conservative (the prediction is worse than chance on UKBB) suggesting that the deconfounding model removes too much variance; confound-isolating cross validation yields more nuanced results, and prediction from confounds yields variable results.

Limitation: with many confounds the problem is harder

Here we have studied the case of one, clearly-identified, confound. The case of multiple confounds (*eg* age, education, gender, ethnicity), is more challenging. In such situations, deconfounding approaches may remove fully the signal of interest. For *confound-isolating cross-validation*, reliable estimation of mutual information will require much larger sample sizes than with a single confound. In practice, we recommend to identify the most impactful confound to run *confound-isolating cross-validation*.

Another concern could be that such confounding factors are not well identified. In that case, the proposed approach does not help, but such a case is very hard to handle with statistical methods (see e.g. [62]). We thus leave handling of imperfect confounder knowledge for future research.

Elements to interpret analyses with confounds

Defining confounds calls for modeling choices

Whether a variable should be considered as a confounding effect or not is not dictated by the data, but by the question at hand. The actual notion of confound comes from causal modeling, to give a causal interpretation to model parameters [15, 63]. Confound variables are then chosen so as to model the difference between the measurements at hand and those obtained with a hypothetical intervention. Such choices are implicitly based on a model of which variables are causes or consequences of the fictional intervention and the outcome of interest [see 64, for guidelines in the case of UKBB].

In pure biomarker settings, the focus is not on potential interventions, but on detecting or predicting an outcome. The concern is then that the measured accuracy might not reflect the actual application settings [27, 22]. Here also, the choice of variables to control for must be governed by an understanding of how the data at hand may differ from ideal data to reflect the target application. More concretely, Confounds can indeed relate to any aspect of the setup, e.g. acquisition devices, data processing routines when these are not homogeneous across all the dataset, measurement-related covariate such as motion, individual conditions, such as age, sex or genetics, that is correlated with the imaging variable and with the outcome.

Deconfounding for causal interpretations: the collider-bias danger

Using deconfounding to cancel the impact of a putative confound $\mathbf{z}$ removes any bias incurred by the spurious association between the data $\mathbf{X}$ and the prediction target $\mathbf{y}$, when $\mathbf{z}$ is associated with both $\mathbf{X}$ and $\mathbf{y}$. However, $\mathbf{z}$ may be a *consequence* of both the target and the data. In such a situation conditioning on it can create a form of selection bias, sometimes known as "collider bias" [65, 66]. Conditioning on the third variable $\mathbf{z}$ can then reverse the correlation between two variables $\mathbf{X}$ and $\mathbf{y}$, a phenomenon known as Berkson's or Simpson's statistical paradox [67, 68]. It can be understood from a simple example: when studying a population of hospital patients, individuals may have been admitted to the hospital because they have disease *A* or *B*. On this specific population, the two diseases are anti-correlated. However, concluding that disease *A* protects from disease *B* would be incorrect. Another example can be found in a cognitive experiment where both a visible-enough stimuli and a timely motor response are needed for a successful response. When learning a model decoding stimuli visibility from brain response, deconfounding on successful responses would lead this model to rely on motor-cortex activity, while the link between visual stimuli and motor cortex is not neuroscientifically relevant as such. Deconfounding by itself does not suffice to yield associations with clear interpretations.

A sampling view on confounds

Confound-isolating cross-validation strives to sample an ideal sub-population. This is also one of the best strategies to avoid the presence of confounds in experimental settings: targeting the recruitment of participants so that the design is balanced, for instance with matched controls or randomized controlled trials. But this can only be done at study design, and targeted acquisitions, with matching and restriction, can make it hard to collect large samples or tackle many covariates. At analysis time, researchers have to rely on statistical methods to adapt the analysis to the presence of confounds. For in-sample analysis, propensity scores are a classic reweighting technique used to obtain reliable effect estimates from confounded datasets [69, 70]. The use of subsampling in *confound-isolating cross-validation* can be seen as an extension of these ideas for out-sample validation of predictive accuracy. The only caveat is that one has to ensure that sampling does not deterministically lead to a fixed test set, which would weaken the statistical guarantees brought by the validation experiment. Here, we propose to perform this check a posteriori. In the future, more complex sampling strategies could be designed to ensure some randomness in the test set.

Conclusion: deconfounding and isolating confounds are complementary

Deconfounding strives to remove confounding effects from the data, after which successful prediction can be interpreted as a direct link from the remaining brain signals to the outcome of interest. However, in biomarkers settings, the primary focus may be on the quality of detection, rather than interpretation, for instance to improve diagnosis or prognosis. In such settings, an important question is: how much do the brain signals improve the prediction upon a simpler measure of the confounding effect? Answering this question calls for a cross-validation procedure isolating this confounding effect. The corresponding prediction accuracy can then safely be interpreted as not resulting in any way from the confounding effect.

Availability of Supporting Source code and Requirements

- Project name: Confound Prediction
- Project home page: https://github.com/darya-chyzhyk/confound_prediction
- Operating system(s): OS independent
- Programming language: Python
- Other requirements: Python (>= 3.5), Scipy (>=1.1.0), Scikit-learn (>=0.21.2), Numpy (>=1.14.2), Pytest(>=5.1.1)
- License: BSD 3-Clause License

Figure 7. Comparisons on tabular data: predicting income from socio-demographics and mental-health, controlling for qualifications. The left column of the figure reports the prediction performance by the mean absolute error for the five approaches considered: Prediction from the data without deconfounding, prediction after deconfounding test and train jointly, prediction with out-of-sample deconfounding, prediction with confound-isolating cross-validation, and prediction from confounds. The left column displays the distribution across validation folds for the actual data (top, orange), and for permuted data distribution (bottom, gray). The right column displays the distribution of p-values across folds, obtained by permutation, and the text yields the aggregated p-value across folds (see the main text), testing whether prediction accuracy is better than chance.

Data Availability

Supporting data and Python implementation to control confound effect are available via the GigaScience database GigaDB [71]

Acknowledgment

This work was funded by the Child Mind Institute Resting state MRI data analysis was done using the UK Biobank Resource under project 25163. We show appreciation to the UK Biobank contributors and participants for collecting and sharing the quality data to researchers. BT was also supported by the SC1-DTH-07-2018 H2020 VirtualBrainCloud Project under grant agreement No 826421, and GV by the DirtyData (ANR-17-CE23-0018-01) project.

References

1. Norman KA, Polyn SM, Detre GJ, Haxby JV. Beyond mind-reading: multi-voxel pattern analysis of fMRI data. Trends in cognitive sciences 2006;10:424.
2. Poldrack RA. Inferring mental states from neuroimaging data: from reverse inference to large-scale decoding. Neuron 2011;72:692.
3. Varoquaux G, Poldrack RA. Predictive models avoid excessive reductionism in cognitive neuroimaging. Current opinion in neurobiology 2019;55:1–6.
4. Plant C, Teipel SJ, Oswald A, Böhm C, Meindl T, Mourao-Miranda J, et al. Automated detection of brain atrophy patterns based on MRI for the prediction of Alzheimer's disease. Neuroimage 2010;50(1):162–174.
5. Wager TD, Atlas LY, Lindquist MA, Roy M, Woo CW, Kross E. An fMRI-based neurologic signature of physical pain. New England Journal of Medicine 2013;368:1388.
6. Abraham A, Milham MP, Di Martino A, Craddock RC, Samaras D, Thirion B, et al. Deriving reproducible biomarkers from multi-site resting-state data: An Autism-based example. NeuroImage 2017;147:736–745.
7. Dosenbach NU, Nardos B, Cohen AL, Fair DA, Power JD, Church JA, et al. Prediction of individual brain maturity using fMRI. Science 2010;329(5997):1358–1361.
8. Liem F, Varoquaux G, Kynast J, et al. Predicting brain-age from multimodal imaging data captures cognitive impairment. NeuroImage 2017;148:179.
9. Woo CW, Chang LJ, Lindquist MA, Wager TD. Building better biomarkers: brain models in translational neuroimaging. Nature neuroscience 2017;20(3):365–377.
10. Engemann DA, Kozynets O, Sabbagh D, Lemaître G, Varoquaux G, Liem F, et al. Combining magnetoencephalography with magnetic resonance imaging enhances learning of surrogate-biomarkers. eLife 2020;9.
11. Cole JH, et al. Brain age predicts mortality. Molecular Psychiatry 2018;23(5):1385–1392.
12. Power JD, Barnes KA, Snyder AZ, Schlaggar BL, Petersen SE. Spurious but systematic correlations in functional connectivity MRI networks arise from subject motion. Neuroimage 2011;59:2142.
13. Miller KL, Alfaro-Almagro F, Bangerter NK, Thomas DL, Yacoub E, Xu J, et al. Multimodal population brain imaging in the UK Biobank prospective epidemiological study. Nature Neuroscience 2016;.
14. Smith SM, Nichols TE. Statistical Challenges in "Big Data" Human Neuroimaging. Neuron 2018;97(2):263 – 268.
15. Pearl J. Causality: Models, Reasoning, and Inference. New York, NY, USA: Cambridge University Press; 2000.
16. Worsley K, Liao C, Aston J, Petre V, Duncan G, Morales F, et al. A general statistical analysis for fMRI data. NeuroImage 2002;15:1.
17. Poldrack RA, Mumford JA, Nichols TE. Handbook of functional MRI data analysis. Cambridge: University Press; 2011.
18. Breiman L. Statistical Modeling: The Two Cultures (with comments and a rejoinder by the author). Statistical Science 2001;16(3):199 – 231. https://doi.org/10.1214/ss/1009213726.
19. Poldrack RA, Huckins G, Varoquaux G. Establishment of best practices for evidence for prediction: a review. JAMA psychiatry 2020;77(5):534–540.
20. Chyzhyk D, Varoquaux G, Thirion B, Milham M. Controlling a confound in predictive models with a test set minimizing its effect. In: PRNI 2018 - 8th International Workshop on Pattern Recognition in Neuroimaging Singapore, Singapore; 2018. p. 1–4. https://hal.archives-ouvertes.fr/hal-01831701.
21. Varoquaux G, Raamana PR, Engemann DA, Hoyos-Idrobo A, Schwartz Y, Thirion B. Assessing and tuning brain decoders: cross-validation, caveats, and guidelines. NeuroImage 2017;145:166–179.
22. Little MA, Varoquaux G, Saeb S, Lonini L, Jayaraman A, Mohr DC, et al. Using and understanding cross-validation strategies. Perspectives on Saeb et al. GigaScience 2017;6:1.
23. Linn KA, Gaonkar B, Doshi J, Davatzikos C, Shinohara RT. Addressing confounding in predictive models with an application to neuroimaging. The international journal of biostatistics 2016;12(1):31–44.
24. Rao A, Monteiro JM, Mourao-Miranda J, Initiative AD, et al. Predictive modelling using neuroimaging data in the presence of confounds. NeuroImage 2017;150:23–49.
25. Friston KJ, Holmes AP, Worsley KJ, Poline JB, Frith C, Frackowiak RSJ. Statistical Parametric Maps in Functional Imaging: A General Linear Approach. Hum Brain Mapp 1995;p. 189.
26. Snoek L, Miletic S, Scholte HS. How to control for confounds in decoding analyses of neuroimaging data. bioRxiv 2018;.
27. Saeb S, Lonini L, Jayaraman A, Mohr DC, Kording KP. The need to approximate the use-case in clinical machine learning. Gigascience 2017;6(5):1–9.
28. Arjovsky M, Bottou L, Gulrajani I, Lopez-Paz D, Invariant Risk Minimization; 2020.
29. Devroye L. Non-Uniform Random Variate Generation(originally published with. Springer-Verlag; 1986. http://cg.scs.carleton.ca/~luc/rnbookindex.html.
30. Devroye L. Non-Uniform Random Variate Generation. Springer-Verlag; 1986.
31. Chyzhyk D. Confound Prediction. GitHub repository 2019;https://github.com/darya-chyzhyk/confound_prediction.
32. Chyzhyk D. Confound Prediction. Python package version 0.0.1a1. The Python Package Index (PyPI) repository 2019;https://pypi.org/project/confound-prediction/.
33. Long X, Benischek A, Dewey D, Lebel C. Age-related functional brain changes in young children. NeuroImage 2017;155:322–330.
34. Zepf FD, Bubenzer-Busch S, Runions KC, Rao P, Wong JWY, Mahfouda S, et al. Functional connectivity of the vigilant-attention network in children and adolescents with attention-deficit/hyperactivity disorder. Brain and Cognition 2017;.
35. Li H, Satterthwaite TD, Fan Y. Brain age prediction based on resting-state functional connectivity patterns using convolutional neural networks. 2018 IEEE 15th International Symposium on Biomedical Imaging (ISBI 2018)

2018;p. 101–104.
36. Franke K, Ziegler G, Klöppel S, Gaser C, Initiative ADN, et al. Estimating the age of healthy subjects from T1-weighted MRI scans using kernel methods: exploring the influence of various parameters. Neuroimage 2010;50(3):883–892.
37. Geerligs L, Tsvetanov K, Cam-CAN, Henson R. Challenges in measuring individual differences in functional connectivity using fMRI: The case of healthy aging. Human Brain Mapping 2017;38(8):4125–4156.
38. Yan CG, Cheung B, Kelly C, Colcombe S, Craddock RC, Martino AD, et al. A comprehensive assessment of regional variation in the impact of head micromovements on functional connectomics. NeuroImage 2013;76:183–201.
39. Satterthwaite TD, Wolf DH, Loughead J, Ruparel K, Elliott MA, Hakonarson H, et al. Impact of in-scanner head motion on multiple measures of functional connectivity: Relevance for studies of neurodevelopment in youth. NeuroImage 2012;60(1):623 – 632.
40. Satterthwaite TD, Elliott MA, Gerraty RT, Ruparel K, Loughead J, Calkins ME, et al. An Improved Framework for Confound Regression and Filtering for Control of Motion Artifact in the Preprocessing of Resting-State Functional Connectivity Data. NeuroImage 2012;.
41. Gilmore A, Buser N, Hanson JL. Variations in Structural MRI Quality Impact Measures of Brain Anatomy: Relations with Age and Other Sociodemographic Variables. bioRxiv 2019;.
42. Van Dijk KRA, Sabuncu MR, Buckner RL. The influence of head motion on intrinsic functional connectivity MRI. Neuroimage 2012;59:431.
43. Finn ES, Shen X, Scheinost D, Rosenberg MD, Huang J, Chun MM, et al. Functional connectome fingerprinting: identifying individuals using patterns of brain connectivity. Nature Neuroscience 2015 oct;18(11):1664–1671.
44. Hearne LJ, Mattingley JB, Cocchi L. Functional brain networks related to individual differences in human intelligence at rest. In: Scientific reports; 2016. .
45. Cattell RB. Abilities : their structure, growth, and action. Houghton Mifflin Boston; 1971.
46. Hartshorne JK, Germine LT. When does cognitive functioning peak? The asynchronous rise and fall of different cognitive abilities across the life span. Psychological science 2015;26:433.
47. Samu D, Campbell KL, Tsvetanov KA, Shafto MA, Cam-CAN consortium, Tyler LK. Preserved cognitive functions with age are determined by domain-dependent shifts in network responsivity. Nature communications 2017 May;8.
48. Bugg JM, Zook NA, DeLosh EL, Davalos DB, Davis HP. Age differences in fluid intelligence: Contributions of general slowing and frontal decline. Brain and Cognition 2006;62(1):9 – 16.
49. Rönnlund M, Pudas S. The neural determinants of age-related changes in fluid intelligence : a pre-registered , longitudinal analysis in UK Biobank; 2018. .
50. Horn JL, Cattell RB. Age differences in fluid and crystallized intelligence. Acta Psychologica 1967;26:107 – 129.
51. Taylor JR, Williams N, Cusack R, Auer T, Shafto MA, Dixon M, et al. The Cambridge Centre for Ageing and Neuroscience (Cam-CAN) data repository: Structural and functional MRI, MEG, and cognitive data from a cross-sectional adult lifespan sample. NeuroImage 2017 jan;144:262.
52. Sudlow C, et al. UK Biobank: An Open Access Resource for Identifying the Causes of a Wide Range of Complex Diseases of Middle and Old Age. PLOS Medicine 2015 03;12(3):1–10.
53. Smith S, Alfaro Almagro F, Miller K. UK Biobank Brain Imaging Documentation 2017;http://biobank.ctsu.ox.ac.uk/crystal/docs/brain_mri.pdf.
54. Alfaro-Almagro F, Jenkinson M, Bangerter NK, Andersson JLR, Griffanti L, Douaud G, et al. Image processing and Quality Control for the first 10,000 brain imaging datasets from UK Biobank. NeuroImage 2018;166:400 – 424.
55. Nichols TE, Das S, Eickhoff SB, Evans AC, Glatard T, Hanke M, et al. Best Practices in Data Analysis and Sharing in Neuroimaging using MRI. bioRxiv 2016;.
56. Dadi K, Rahim M, Abraham A, Chyzhyk D, Thirion B, Varoquaux G. Benchmarking functional connectome-based predictive models for resting-state fMRI. NeuroImage 2019;192:115–134.
57. Bellec P, Rosa-Neto P, Lyttelton OC, Benali H, Evans AC. Multi-level bootstrap analysis of stable clusters in resting-state fMRI. NeuroImage 2010;51:1126.
58. Varoquaux G, Baronnet F, Kleinschmidt A, Fillard P, Thirion B. Detection of brain functional-connectivity difference in post-stroke patients using group-level covariance modeling. In: MICCAI; 2010.
59. Abraham A, Pedregosa F, Eickenberg M, Gervais P, Mueller A, Kossaifi J, et al. Machine learning for neuroimaging with scikit-learn. Frontiers in Neuroinformatics 2014;8:14.
60. Meinshausen N, Meier L, Bühlmann P. P-values for high-dimensional regression. Journal of the American Statistical Association 2009;104(488):1671–1681.
61. Zhao Q, Adeli E, Pohl KM. Training confounder-free deep learning models for medical applications. Nature Communications 2020 Nov;11(1):6010. https://doi.org/10.1038/s41467-020-19784-9.
62. Brumback BA, Hernán MA, Haneuse SJ, Robins JM. Sensitivity analyses for unmeasured confounding assuming a marginal structural model for repeated measures. Stat Med 2004 Mar;23(5):749–767.
63. Angrist JD, Pischke JS. Mostly harmless econometrics: An empiricist's companion. Princeton university press; 2008.
64. Alfaro-Almagro F, McCarthy P, Afyouni S, Andersson JLR, Bastiani M, Miller KL, et al. Confound modelling in UK Biobank brain imaging. NeuroImage 2020;p. 117002. http://www.sciencedirect.com/science/article/pii/S1053811920304882.
65. Cole SR, Platt RW, Schisterman EF, Chu H, Westreich D, Richardson D, et al. Illustrating bias due to conditioning on a collider. International journal of epidemiology 2009;39(2):417–420.
66. Greenland S. Quantifying biases in causal models: classical confounding vs collider-stratification bias. Epidemiology 2003;14(3):300–306.
67. Berkson J. Limitations of the application of fourfold table analysis to hospital data. Biometrics Bulletin 1946;2(3):47–53.
68. Simpson EH. The interpretation of interaction in contingency tables. Journal of the Royal Statistical Society: Series B (Methodological) 1951;13(2):238–241.
69. Rubin DB. Estimating causal effects from large data sets using propensity scores. Annals of internal medicine 1997;127(8_Part_2):757–763.
70. Becker SO, Ichino A. Estimation of average treatment effects based on propensity scores. The stata journal 2002;2(4):358–377.
71. Chyzhyk D, Thirion B, Varoquaux G, Milham MP. Supporting data for "How to remove or control confounds in predictive models, with applications to brain biomarkers". GigaScience Database 2022;http://dx.doi.org/10.5524/100980.
72. Pypreprocess. GitHub repository 2019;https://github.com/neurospin/pypreprocess.
73. Gorgolewski K, Burns CD, Madison C, Clark D, Halchenko YO, Waskom ML, et al. Nipype: a flexible, lightweight

and extensible neuroimaging data processing framework in python. Frontiers in neuroinformatics 2011;5:13.

Appendices

Data preprocessing

CamCan data were preprocessed using Pypreprocess [72], a collection of Python scripts for preprocessing fMRI data, that is based on the SPM12 software and the nipype toolbox [73]. We preprocessed CamCan data only. For UKBB data the preprocessed and connectivity matrices are available from the data repository. We apply a commonly used protocol that includes the following steps: Motion correction, correction for subject's head motion during the acquisition. Estimated six motion parameters (three translational parameters and three rotational parameters) are used as confounds in the age prediction experiments. For each subject we expressed the head motion using translation across all three axes as a square root of the mean of the sum of square finite difference of each translation axes over the time: $\sqrt{\frac{\vec{\Delta}translation_x^2 + \vec{\Delta}translation_y^2 + \vec{\Delta}translation_z^2}{3}}$
The rest-fMRI data are coregistered to the anatomical T1-MRI and then normalized to MNI template.

Supplementary results on the resting state data sets

Figure 8. Evolution of mutual information and correlation with the number of subjects for different subsampling methods on the CamCan dataset with Fluid Intelligence prediction and UKBB Age prediction. This figure shows that the proposed method effectively reduces statistical dependencies between confound and target (red curve) for both data sets and both predictors.

Supplementary results on simulated data, 1000 samples

Figure 9. Benchmarking approaches to control confounded predictions on simulated data with many samples. The left column of each sub-figure assesses the prediction performance through the mean absolute error (in signal units). We display the error distribution across validation folds for the data (top, orange), and for permuted data distribution (bottom, gray). The right column displays the distribution of p-values across folds, obtained by permutation, and the text reports the aggregated p-value across folds (see the main text). Five approaches are benchmarked: Without deconfounding, Deconfounding test and train jointly, Out-of-sampling deconfounding, Confound-isolating cross-validation, and Prediction from confounds. There are three simulation settings: (a) No direct link between target and brain, (b) A direct link between target and brain and (c) A weak confound and a direct link between target and brain. Green ticks indicate correct conclusions, red crosses mark incorrect ones, and warning signs the weak results.

Manuscript

Click here to access/download;Manuscript;GigaScience-2022-01.pdf

OXFORD (GIGA)^n SCIENCE

GigaScience, 2020, 1–12

doi: xx.xxxx/xxxx
Manuscript in Preparation
Paper

PAPER

How to remove or control confounds in predictive models, with applications to brain biomarkers

Darya Chyzhyk [1,2,3,*], Gaël Varoquaux [1,2], Michael Milham [3,4] and Bertrand Thirion [1,2]

[1]Parietal project-team, INRIA Saclay-île de France, France and [2]CEA/Neurospin bât 145, 91191 Gif-Sur-Yvette, France and [3]Center for the Developing Brain, Child Mind Institute, New York, New York 10022, USA and [4]Center for Biomedical Imaging and Neuromodulation, Nathan S. Kline Institute for Psychiatric Research, Orangeburg, New York 10962, USA

[*]darya.chyzhyk@gmail.com

Abstract

Background
With increasing data sizes and more easily available computational methods, neurosciences rely more and more on predictive modeling with machine learning, *eg* to extract biomarkers of pathologies. Yet, a successful prediction may capture a confounding effect correlated with the outcome instead of brain features specific to the outcome of interest –*eg* the pathology. For instance, as patients tend to move more in the scanner than controls, imaging biomarkers of a pathology may mostly reflect head motion, leading to inefficient use of resources and wrong interpretation of the biomarkers.
Resuts
Here we study how to adapt statistical methods that control for confounds to predictive modeling settings. We review how to train predictors that are not driven by such spurious effects. We also show how to measure the unbiased predictive accuracy of these biomarkers, based on a confounded dataset. For this purpose, cross-validation must be modified to account for the nuisance effect. To guide understanding and practical recommendations, we apply various strategies to assess predictive models in the presence of confounds on simulated data and population brain imaging settings. Theoretical and empirical studies show that deconfounding should not be applied to the train and test data jointly: *modeling* the effect of confounds, on the train data only, should instead be decoupled from *removing* confounds.
Conclusions
Cross-validation that isolates nuisance effects gives an additional piece of information: confound-free prediction accuracy.

Key words: confound, subsampling, phenotype, predictive models, biomarkers, statistical testing, deconfounding

Introduction

Predictive models, using machine learning, are becoming a standard tool for scientific inference. In cognitive neuroscience, they can be used for *decoding*, to make conclusions on mental processes given observed brain activity [1, 2, 3]. With the rise of large-scale brain-imaging cohorts, they can extract imaging biomarkers that predict across subjects phenotypes such as neuropsychiatric conditions [4, 5, 6] or individual traits [7, 8].

A crucial aspect of these biomarkers is their ability to *predict* the outcome of interest, *ie* to generalize to new data [9]. However, these predictions can be driven by confounding effects. Such effects affect both the brain-imaging data and the prediction target but are considered as irrelevant. For instance, brain imaging reflects age quite accurately, and actually carries infor-

Compiled on: January 22, 2022.
Draft manuscript prepared by the author.

mation about age-related diseases [8, 10, 11], yet [12] showed that subjects' in-scanner motion varies with subjects' age and it creates systematic differences in recorded brain imaging signals. Given this confounding effect, MRI biomarkers of brain aging may be nothing more than expensive measurements of head motion. Other examples may be more subtle: age matters for diagnosing Alzheimer's disease, yet an important question is whether brain imaging yields an accurate diagnosis of Alzheimer disease beyond the mere effect of age.

More generally, the data at hand often capture effects not of direct interest to the investigation. In many situations, some confounds such as head motion cannot be fully avoided. To make matters worse, large cohorts developed in population imaging to answer epidemiological questions [as UK biobank, 13] are observational data: there is no controlled intervention or balanced case-control group; rather, individuals are recruited from diverse populations with various sampling or selection biases. To conclude on the practical use of biomarkers, it is important to ensure that their predictions are not fully driven by such unwanted effects. This requires measuring model predictive accuracy after controlling for nuisance variables. Confounding effects can also make it hard to interpret brain-behavior relationships revealed by predictive models [14], as confounds can mediate the observed association or be a latent common cause of observations [15].

In experimental settings, *eg* as in a small cohort, confounding can be suppressed by balancing the acquisition for confounds, or using randomized control trials. However, constraints in the data acquisition, *eg* recruitment of a large cohort, often imply that confounds are present in the data, and appropriate analysis is needed to avoid reaching erroneous conclusions. The statistical literature on controlling confounding variables is well developed for classic statistical analysis, such as statistical testing in a linear model at the heart of the standard mass-univariate brain mapping [16, 17]. However, these procedures need to be adapted to high-dimensional predictive-modeling settings, where the focus is to achieve high-prediction accuracy based on imaging data. Indeed, predictive models do not rely on the same parametric assumptions, namely linearity of effects and Gaussian noise. Often, a predictive analysis does not build on a generative model of the signal but on optimizing discrimination [18]. In addition, predictive models draw their purpose and validity from out-of-sample prediction, rather than in-sample statistical testing [19]. The question tackled here is thus whether one can assess the predictive accuracy of brain measurements free of unwanted confounds. It is *not* to identify treatment effects size nor to perform other types of causal inference.

In this paper, we study statistical tools to control for confounding effects in predictive models. We consider that practitioners should primarily avoid or reduce the impact of confounds on their model, but this is not always feasible or maybe hard to check, hence, we choose to put the emphasis on the unbiased evaluation of models in the presence of confounds. A preliminary version of the work discussed here was presented at the PRNI conference [20]. While the core method is the same, it presents limited insights on the theoretical underpinnings and practical value of the method proposed. Experiments on simulated data are absent and experiments on neuroimaging data are limited to just one data set. In particular, statistical significance is not established thoroughly, and only one alternative approach is considered. In short the conference publication provides limited insights on the method, while the current work provides a complete description and points to the code for reuse.

We first review how the classic deconfounding procedures can be used in predictive-modeling settings, i.e. together with cross-validation. We then expose a complementary approach that is not based on removing confounding effects, but rather testing whether a given predictive model –*eg* a biomarker– predicts well when these confounds are not present. For this we introduce the *confound-isolating cross-validation* method, that consists in sampling test sets in which the effect of interest is independent from the confounding effect. The benefits of this approach are that it is non-parametric and that it directly tests the quantity of interest in a predictive analysis. We then run an extensive empirical study on three population-imaging biomarker extraction problems, a tabular dataset, as well as simulations. We draw practical recommendations to test predictive models in the presence of confounding effects.

Methods: controlling for confounds in predictive models

Formalizing the problem of prediction with a confound

Assessing predictive models

Predictive models are assessed by their prediction accuracy [19]. For this, cross-validation is the standard tool, typically k-fold cross-validation [21]. It consists in partitioning (potentially randomly) the original dataset into k equal size subsets or folds (each denoted by a color in Figure 1). One of these k sets is held out for testing, and the remaining $(k-1)$ folds are used for training the model. This process is repeated k times, where each time a different group of observations compose the test set. Prediction accuracy is measured on the test set, then averaged across folds.

Confounding variables in a prediction task

To formalize prediction in presence of a confound, we consider a dataset of n observations –*eg* subjects or time-points– comprising $p-$ *dimensional* brain signals $\mathbf{X} \in \mathbb{R}^{n \times p}$, an effect of interest[1] $\mathbf{y} \in \mathbb{R}^n$ –the biomarker target– and a confounding effect $\mathbf{z} \in \mathbb{R}^n$.

An imaging biomarker then predicts $\mathbf{y}$ from $\mathbf{X}$. If $\mathbf{X}$ and $\mathbf{z}$ on the one hand, $\mathbf{y}$ and $\mathbf{z}$ on the other hand, are not independent, the prediction of the target $\mathbf{y}$ might be affected or most accurately done by the confounding effect, $\mathbf{z}$. Such prediction may be misleading or useless. It can be misleading as it can be interpreted as a link between brain structures and $\mathbf{y}$ –*eg* fluid intelligence– while such a link only reflects the effect of $\mathbf{z}$ –*eg* age. It can be useless because brain imaging is likely much more costly to acquire than the phenotypic variable $\mathbf{z}$, hence it should be used only if it brings more diagnostic information. Moreover, this can be detrimental to accuracy: if a future dataset shows an altered relation between the confound and the features, prediction accuracy may be compromised.

A crucial problem for the validity of the biomarker is to measure whether it can predict $\mathbf{y}$ from $\mathbf{X}$ and not solely from $\mathbf{z}$. Prediction accuracy is ideally measured on an independent validation set, but most often, no large independent validation set is available and a cross-validation procedure, that iteratively separates train and test sets [21], is used. In [22] what cross-validation captures in the presence of a confounding variable is discussed. Though there can be many possible confounds in brain imaging (see section 8), we focus below on simple settings, assuming that the main confounding factor has been isolated in one variable.

There are two points-of-view to controlling confounds in predictive models. One is to try and *remove* the effect of the con-

1 In classification settings, $\mathbf{y}$ does not take continuous values in $\mathbb{R}^n$, yet we use the most general notation to cover both classification and regression settings.

founding variables from the data, by regressing them out (deconfounding) or resampling the data to cancel spurious correlations (re-balancing). The other is to test that the model's prediction captures more than the confound. Removing the confounding signal can test whether predictions are fully driven by the confound $\mathbf{z}$ rather than the brain signal $\mathbf{X}$. However, it does not provide a good tool to measure the predictive power in the presence of confounds: the accuracy is likely biased, as illustrated later in the simulations.

Another point of view on confounding effects in predictive modeling consists in trying to learn a predictor from a biased population –with the confounding effect– that differs from the population of interest –without the confounding effect. The problem can then be tackled as a *domain adaptation* problem [23, 24]. However, [24] have shown that compensating for the confound does not improve prediction if the test population is not markedly different from the training population. Note that train and test samples are often drawn from the same population, either because only one cohort is available or because a proper stratification scheme is used. Our question is different: we are interested in assessing whether learning a biomarker on a confounded dataset leads to predictions that are fully driven by the confound.

Deconfounding

Deconfounding in standard analysis

In inferential statistics –as opposed to predictive modeling– proper modeling of confounds is important to control the *interpretation* of model parameters, ensuring that they are not driven by the confounding effects. Classical statistic analysis in brain imaging is based on the general linear model (GLM) [25, 16], in which confounding effects are controlled by additional regressors to capture the corresponding variance. Such an approach shows limitations in predictive-modeling settings. First, it is based on maximum-likelihood estimates of linear models, while in general, predictive models are not explicitly based on a likelihood and are often not linear. Second, it is designed to control *in-sample* properties, while predictive models are designed for *out-of-sample* prediction. The two-step approach based on applying a classical GLM to remove the confounding effect, then a predictive model, may lead to pessimistic results, *eg* below-chance prediction [8, 26].

In the context of the GLM, an alternative implementation relies on removing the effect of variables that are correlated. [25]. Note that in all this work we assume that the confounder is associated with $\mathbf{X}$ and $\mathbf{y}$ without creating three ways interactions between $\mathbf{X}$, $\mathbf{y}$ and $\mathbf{z}$. Given a sample $\mathbf{X} \in \mathbb{R}^{n \times p}$ of n observations (subjects) with p brain imaging features (*eg* connectivity matrices), $\mathbf{X}_i = (\mathbf{X}_{i1}, \mathbf{X}_{i2}, \ldots, \mathbf{X}_{ip})$ and confounds $\mathbf{z} \in \mathbb{R}^n$, the model is:

$$\mathbf{X} = \mathbf{z}^{\mathbf{T}}\mathbf{w} + \mathbf{e}, \tag{1}$$

where $\mathbf{w}$ is a vector of weights (one per voxel, $\mathbf{w} \in \mathbb{R}^p$). $\hat{\mathbf{w}}$ represents the estimated coefficients, that are obtained typically through least-squares regression:

$$\hat{\mathbf{w}} = (\mathbf{z}^{\mathbf{T}}\mathbf{z})^{-1}\mathbf{z}^{\mathbf{T}}\mathbf{X} \tag{2}$$

Given these equations, a linear model can be used prior to the predictive model to remove the effect of the confounds $\mathbf{z}$ on the brain signals $\mathbf{X}$. It must be adapted to out-of-sample testing. One solution is to apply deconfounding jointly on the train and the test set, but it breaks the statistical validity of cross-validation because it couples the train and the test set [21]. Hence it can give biased results.

Out-of-sample deconfounding

To adapt the above *deconfounding* approach to the two phases of training and testing a predictive model, a useful view is to consider the deconfounding model as a predictive encoding model, predicting a fraction of the signal $\mathbf{X}$ from $\mathbf{z}$. Deconfounding is then performed by removing the part of the signal captured by $\mathbf{z}$ from $\mathbf{X}$:

$$\hat{\mathbf{X}}_{\mathbf{clean}} = \mathbf{X} - \mathbf{z}\hat{\mathbf{w}} \tag{3}$$

Where $\hat{\mathbf{w}}$ are the coefficients of the linear deconfounding model (Equation 1), estimated on the train data with Equation 2 and then applied to the test [26]. The full out-of-sample deconfounding procedure is listed in algorithm 1.

A drawback of such deconfounding is that it is strongly parametric, i.e. it relies on the model of confounds used. Equation 2 stands for the classic linear model, assuming linearity between the confounding variable $\mathbf{z}$ and the brain signal $\mathbf{X}$. The linear model only takes into account second-order statistics (covariance or correlations) and ignores more complex dependencies.

Model-agnostic out-of-sample deconfounding

A common solution to go beyond linear effects of confounds is to use a polynomial expansion of the confounds $\mathbf{z}$ in the linear deconfounding model. Another option is to use a more powerful predictive model in the confound removal. A predictive model –including a mere linear model– regressing $\mathbf{X}$ on $\mathbf{z}$ can be seen as estimating a function f so that $f(\mathbf{z}) = \mathbb{E}[\mathbf{X}|\mathbf{z}]$. There are many possibilities such as random forests or Gaussian processes. The procedure used for out-of-sample deconfounding can then be adapted as in Algorithm 2. While this approach is very powerful, the danger is to remove also part of the signal of interest. Indeed, using a more powerful predictive model, for instance a higher-order polynomial, leads to explaining in $\mathbf{X}$ more data as a function of $\mathbf{z}$; however too powerful models *overfit*, which means that they explain variance in $\mathbf{X}$ by chance. In such a situation, the deconfounding procedure may remove signal of interest, unrelated to the confound.

Comparing predictive power of confounds

A simple evaluation of the impact of $\mathbf{z}$ on the prediction of $\mathbf{y}$ is to use predictive models predicting $\mathbf{y}$ from $\mathbf{z}$ (*prediction from confound*) and compare the predictive accuracy to that obtained with biomarkers based on brain signals. This argument is used in [6] to control for the effect of movement on autism diagnostic.

Creating a test set to isolate the confounding effect

Rather than deconfounding, the investigator may ensure that the predictive model is useful by measuring its accuracy on a dataset where the confounding effect is absent. In a cross-validation setting, such a situation can be created by using as a test set a well-chosen subset of the data that isolates the con-

Algorithm 1: Out-of-sample deconfounding

Input: Brain signal $\mathbf{X} \in \mathbb{R}^{n \times p}$, confound $\mathbf{z} \in \mathbb{R}^n$, {train} and {test} indices

1 $\hat{\mathbf{w}}_{\mathbf{confounds}} \leftarrow (\mathbf{z}^{\mathbf{T}}{}_{\mathbf{train}}\mathbf{z}_{\mathbf{train}})^{-1}\mathbf{z}^{\mathbf{T}}{}_{\mathbf{train}}\mathbf{X}_{\mathbf{train}}$
`/* Regression of confounds on data */`

2 $\hat{\mathbf{X}}_{\mathbf{clean,test}} \leftarrow \mathbf{X}_{\mathbf{test}} - \mathbf{z}_{\mathbf{test}}\hat{\mathbf{w}}_{\mathbf{confounds}}$
`/* Remove confounds in the test set */`

Output: Brain signal without confounds $\hat{\mathbf{X}}_{\mathbf{clean,test}}$

Algorithm 2: Model-agnostic deconfounding. Note that f only has one argument, as it is a function that predicts $\mathbf{X}$ from $\mathbf{z}$, while g has two arguments (the input $\mathbf{X}$ and the output $\mathbf{z}$), as it represents the learning algorithm that yields f.

Input: Brain signal $\mathbf{X} \in \mathbb{R}^{n \times p}$, confound $\mathbf{z} \in \mathbb{R}^n$, {train} and {test} indices, machine-learning algorithm g

```
f ← g(z_train, X_train)
                        /* Fit confound model capturing E[X|z] */
X̂_clean,test ← X_test − f(z_test)
                        /* Remove confounds in the test set */
```

Output: Brain signal without confounds $\hat{\mathbf{X}}_{\text{clean,test}}$

Figure 1. Classic and confound-isolating cross-validation. a) k-fold cross-validation is the common procedure to evaluate predictive models. It consists in splitting the data into k equal groups. k-1 folds are used to fit a model and 1 fold is used to validate the model. This process is repeated k times so that each sample is taken once in the test set. b) In *confound-isolating cross-validation* sampling we divide the data in train and test sets, but in a different way. First, using subsampling, we create a test set on which $\mathbf{y}$ and $\mathbf{z}$ are independent. The train test is constructed from the rest of the samples that are not included in the test set. In this way, the method creates a test set that contains unrelated target and confound.

founding effect. See Figure 1 for a graphical illustration of the approach. Formally, it requires choosing a subset $\mathcal{S}$ of the data such that $\mathbf{y}_\mathcal{S}$ and $\mathbf{z}_\mathcal{S}$ are independent (the feasibility of this subset creation is discussed below).

The remainder of the data is used as a training set, to learn to predict $\mathbf{y}$ from $\mathbf{X}$. If the prediction generalizes to the test set $\mathcal{S}$, the learned relationship between $\mathbf{X}$ and $\mathbf{y}$ is not entirely mediated by $\mathbf{z}$. In particular, the prediction accuracy then measures the gain in prediction brought by $\mathbf{X}$.

Categorical confound

The confounding effect can be "categorical", for instance the site effect when learning predictive biomarkers on multi-site acquisitions as in [6]. In such settings, to test that the model can indeed predict independently from site effects, a simple solution is to resort to a cross-validation that avoids having samples from the same site both in the train and the test sets. This may imply resampling the data to cancel out associations between site and target related to data imbalance. Similarly, in multi-subject prediction with repeated measurements from the same subject, subject-wise cross-validation can rule out that prediction is driven by subject identification [27, 22]. More generally, for a categorical confound $\mathbf{z}$, having distinct values for $\mathbf{z}$ in the train and the test set ensures that the prediction cannot be driven by $\mathbf{z}$. We note that this procedure is different from the stratification strategy used in classical statistics, but it clearly avoids any bias due to imperfectly corrected association between $\mathbf{z}$ and the other variables. In the case of site-related confounds, prediction accuracy will obviously suffer. This can be addressed with techniques such as invariant risk minimization [28], but we do not further consider this approach here.

Continuous confound

When $\mathbf{z}$ is a continuous variable, such as age, it is more challenging to generate test sets on which $\mathbf{y}_\mathcal{S}$ and $\mathbf{z}_\mathcal{S}$ are independent. We describe here an algorithm to generate such sampling, "confound-isolating cross-validation" subsampling. It is based on iterative sampling to match a desired distribution: the goal is to have a test set with independence between $\mathbf{y}$ and $\mathbf{z}$, i.e. $p(\mathbf{y}, \mathbf{z}) = p(\mathbf{y})\,p(\mathbf{z})$, where $p((\mathbf{y}, \mathbf{z}))$ is the joint probability function of $\mathbf{y}$ and $\mathbf{z}$, and $p(\mathbf{y})$ and $p(\mathbf{z})$ are the marginal probability distribution.

A related quantity is mutual information, which characterizes the level of dependency between the two variables: $\eta(\mathbf{y}, \mathbf{z}) = \mathbb{E}\left[\log\left(\frac{p((\mathbf{y},\mathbf{z}))}{p(\mathbf{y})p(\mathbf{z})}\right)\right]$. In practice we estimate the probability density functions with a kernel-density estimator (KDE) using Gaussian kernels. We iteratively create the test $\mathcal{S}$ set by removing subjects; at each iteration, we consider the problem as a distribution matching problem, matching $p(\mathbf{y}_\mathcal{S}, \mathbf{z}_\mathcal{S})$ and $p(\mathbf{y}_\mathcal{S})\,p(\mathbf{z}_\mathcal{S})$. For this, we use importance sampling: we draw randomly 4 subjects to discard with a probability $\frac{p(\mathbf{y}_\mathcal{S}, \mathbf{z}_\mathcal{S})}{p(\mathbf{y}_\mathcal{S})\,p(\mathbf{z}_\mathcal{S})}$ using inverse sampling method [30, sec 2.2]. Algorithm 3 gives the details. The choice of 4 samples is tailored to the sample size considered here: it makes the algorithm faster than using one sample, yet is low enough not to compromise mutual information minimization. A Python implementation is available on GitHub [31] and on PyPI repository [32] and can be installed with `pip install confound-prediction`.

Note that if $\mathbf{z}$ and $\mathbf{y}$ are too strongly related, the subsampling procedure above does not have enough degrees of freedom and may always chose the same subset: the test set would be deterministically defined by the sampling procedures, in which case there would effectively be only one fold of cross-validation. In practice, it is important to check that such a situation does not occur when analyzing a given dataset. One way is to compute the average fraction of common samples between two tests sets created with different seeds. As this value ranges from 0 to 1, where 1 means that all test sets contain the same samples and 0 that test sets have no sample in common, it is important to check that it is low enough.

Empirical study methodology

We now describe the experimental materials underlying our empirical study of confound-controlling approaches in predictive models.

Simulation studies

To understand the behavior of the different accuracy scores, we present experiments on simulated data. We simulate a data set $\mathbf{X_0} \sim \mathcal{N}(0, 1)$ with confound $\mathbf{z_0} \sim \mathcal{N}(0, 1)$ to predict continuous variable $\mathbf{y} \sim \mathcal{N}(0, 1)$. We evaluate two samples sizes: $n = 100$ and $n = 1000$. We use $p = 100$ features in $\mathbf{X_0}$. We study 3 scenarios:

- **No direct link between target and brain** where the brain signal does not provide any direct information to predict $\mathbf{y}$, but is observed with a confound linked to $\mathbf{y}$:

Algorithm 3: Confound-isolating cross-validation

Input: Target $\mathbf{y} \in \mathbb{R}^n$, confound $\mathbf{z} \in \mathbb{R}^n$, size $m < n$

```
S ← {1...n}                                  /* Initialize */
while card(S) > m do
    p_y ← KDE(y_S)                   /* Density estimation */
    p_z ← KDE(z_S)
    p_(y,z) ← KDE((z_S, y_S))
    m_i ← p_(y,z)((z_i, y_i)) / (p_y(y_i) p_z(z_i)),  ∀i ∈ S
    S ← S − {j} Draw one index j to remove from S with
        probability m_j using inversion sampling [29].
end
```

Output: Set of test indices $\mathcal{S}$

$$\text{observed confound } \mathbf{z} = \mathbf{y} + \mathbf{z_0},$$
$$\text{observed signal } \mathbf{X} = \mathbf{X_0} + \mathbf{z}.$$

- **Direct link between target and brain** where the brain signal does indeed provide information to predict **y** and has an additional confound linked to **y**:

$$\text{observed confound } \mathbf{z} = \mathbf{y} + \mathbf{z_0},$$
$$\text{observed signal } \mathbf{X} = \mathbf{X_0} + \mathbf{y} + \mathbf{z}.$$

- **Weak confound & direct link between target and brain**

$$\text{observed confound } \mathbf{z} = 0.5\,\mathbf{y} + \mathbf{z_0},$$
$$\text{observed signal } \mathbf{X} = \mathbf{X_0} + \mathbf{y} + \mathbf{z_0}.$$

Note that one could consider instead a canonical scheme in which **z** would cause **x** and **y**. Since our work is not on causal inference *per se*, we aim at a statistical procedure that does not require a prescribed causal relationship between the variables, which is often unknown.

Two classic confounded predictions in population imaging

Motion confounding brain-age prediction

As brain aging is a risk factor of many pathologies, the prediction of brain age from MRI is a promising biomarker [11]. In childhood also, markers of functional brain development can help to recognize neurodevelopmental disorders [33, 34]. Many recent studies report age prediction, *eg* from resting-state functional connectivity [7, 33, 35], from structural imaging [36], or combining multiple imaging modalities [8, 10]. However, older people and children move more in the scanner than young adults [see fig. 2, 37, 38, 12, 39]. Thus, age-related changes observed in brain images may be confounded by head motion [40] and image quality [41].

Indeed, in-scanner motion creates complex MRI artifacts that are difficult to remove [40]. In addition, they severely impact measurements of functional connectivity [42].

Here the confounding effect is that of head motion during the few hundreds of scans of individual acquisitions. To build a variable summarizing head motion for each subject, we use the movement time-series computed during preprocessing. As suggested in [42], we create the confound **z** from the root mean squared displacements (position differences across consecutive time points) for each subject $z = \sqrt{\frac{1}{I-1}\sum_{i=2}^{I}\left((t_x^i - t_x^{i-1})^2 + (t_y^i - t_y^{i-1})^2 + (t_z^i - t_z^{i-1})^2\right)}$, where t_x is left/right, t_y anterior/posterior, and t_z - superior/inferior translation and $i \in [\![I]\!]$ is the time index. The prediction target **y** is the age in years.

Age confounding fluid-intelligence measures

Various studies have predicted individual cognitive abilities from brain functional connectivity [43, 44]. In particular, [44] used machine-learning to predict fluid intelligence from rest fMRI. Fluid Intelligence quantifies the ability to solve novel problems independently from accumulated knowledge, as opposed to crystallized intelligence that involves experience and previous knowledge [45]. It is well known that cognitive abilities change with age [46, 47, 48, 49], in particular that fluid Intelligence progressively declines in middle age [50], while crystallized intelligence continues to grow with age. Indeed, in a cohort with a large age span, the data display a strong relation between fluid intelligence and age (Figure 2). When extracting biomarkers of fluid intelligence, the danger is therefore to simply measure age. We study how to control the impact of age when predicting a fluid-intelligence score from rest-fMRI functional connectivity.

Population-imaging rest-fMRI datasets

Datasets

We ran experiments on 626 participants from the CamCan data set and 9 302 participants from UKBB. All participants are healthy subjects with no neurological disorders.

- **CamCan** Cambridge Center for Ageing and Neuroscience data [51] studies age-related changes in cognition and brain anatomy and function. Characteristics of interest of this dataset are *i)* a population lifespan of 18-88 years, *ii)* a large pool (626 subjects) of multi-modal MRI data and neurocognitive phenotypes.
- **UKBB** The UK Biobank project [52] is a prospective epidemiological study to understand the development of diseases of UK population over the years. The data used here contains 9 302 subjects from the first release of UK Biobank ongoing cohort study with available resting-state fMRI scans and extensive health and lifestyle information [53, 54].

Table 1 presents detailed information about the number of subjects and the scale of the scores for each data set.

We give detailed information on pre-processing steps for each dataset in Appendix 8, following COBIDAS recommendations [55].

Prediction from functional connectivity

To build predictive models from resting-state fMRI, we follow the recommendations in [56]. We use the BASC functional atlas [57] with 64 regions, based on which we extract fMRI time series from the CamCAN dataset. Next, we normalize, detrend and bandpass-filter between 0.01 and 0.1Hz the signal. We represent connectivity matrices with tangent parametrization [58]. Finally, we use a ridge regression with nested cross-validation to learn predictive biomarkers from the functional-connectivity matrices. We use Nilearn [59] for the whole predictive pipeline.

Tabular (non-imaging) data

The considerations on confounds in predictive models are not specific to imaging data. We also study a confounded prediction without brain signals: on the UKBB data, we consider predicting an individual's income from socio-demographics and mental-health assessments. We investigate education as a potential confound: it may be reflected both in mental-health and in income. There are 8 556 individuals with no missing values on the outcome and confound. We use random forests for prediction, as it is a popular learner that is well suited to the distribution of these tabular data, that is often non-Gaussian and consists of categorical variables.

Experimental paradigm: cross-validation measures

We use cross-validation to assess prediction accuracy. We consider five predictive frameworks: (1) Without deconfounding, (2) Deconfounding test and train sets jointly, (3) Out-

Table 1. Characteristics of the data used. The scores for Fluid Intelligence differ on the two datasets: CamCan uses the Cattell test, and UKBB a specifically-designed touch-screen questionnaire.

Dataset Information	**CamCan**	**UKBB**
Number of subjects	626	9 302
Age	18 – 88	40 – 70
Fluid Intelligence scale	Cattell (11 – 44 scores)	UKBB-designed (1 – 13 scores)

of-sampling deconfounding, (4) Confound-isolating cross-validation, (5) Prediction from confounds only. The code for these various strategy to control for confounds can be found on GitHub [31] and on PyPI repository [32] and can be installed with `pip install confound-prediction`.

We use 10 folds, with random splits of 20% of the data in the test set. For *confound-isolating cross-validation*, different seeds in the random number generator lead to different folds. We assess the null distribution of predictions with permutations (20 000 folds on permuted labels $\mathbf{y}$).

Results of the empirical study

Simulated data

We first consider simulated data, for which there is a ground truth. Figure 3 shows the results of the different methods to control for confounds on 3 different simulated cases (Figure 9 gives results for the same simulations with 1 000 samples).

(a) In the case where there is no direct relationship between the data and the target, the performance of the prediction model should not be better than chance after controlling for the confound. Both joint deconfounding and *confound-isolating cross-validation* clearly reveal that all the prediction is mediated by the confound. Out-of-sample deconfounding displays a less clear signal, as there seems to be a slight prediction even after deconfounding, though it is not significant.

(b) For a direct link between the data and the target, joint deconfounding yields a false negative, in the sense that it fully removes the prediction from the brain signal: it is too aggressive in removing signal. Other approaches correctly support a successful prediction.

(c) For a weaker confounding signal, results are similar, however it is worth noting that the target can no longer be well predicted from the confound.

Overall, on the simulations, both *out-of-sample deconfounding* and *confound-isolating cross-validation* give reliable answers, while deconfounding the test and train jointly as well as measuring the prediction from confounds cannot be trusted.

Experiments on resting-state fMRI data

Potential confounds

Figure 2 shows the relationships between target variable y and confounds z. Fluid Intelligence (target) is strongly negatively correlated with age (confound) on the CamCan dataset (second column of Figure 2). Also, on the CamCan data, Age and Motion are very correlated (first column of Figure 2). On the more homogeneous and larger UKBB sample (9 302 subjects), this link is weaker.

Confound-isolating cross-validation

Figure 4 displays the evolution of the association between confound and target during *Confound-isolating cross-validation* in the CamCan dataset, predicting Fluid Intelligence with Age as a confound. In the full dataset, comprising 608 subjects, the correlation between confound and target is $\rho = -0.67$. Iterating the algorithm to remove half of the subjects leads to $\rho = -0.17$. The final test set contains 1/5 of the initial set of subjects and achieves $\rho = -0.07$, showing that it indeed cancels the dependency between aging and motion. The joint distribution between target and confound displayed in Figure 4 shows that the initial statistical dependency between these two variables vanishes after a few tens of iterations of the algorithm. Quantitative evaluation, measuring both Pearson correlation and mutual information (Figure 5) confirms that the confound-isolating procedure efficiently creates a subset of the data without the dependency as soon as it reduces the data to 300 subjects or less. Figure 8 shows similar success on the other prediction problems that we study.

In a cross-validation setting, the different test sets should probe different subjects to maximize testing power. A risk, when using *confound-isolating cross-validation*, is that it could repeatedly generate test sets with the same samples. To measure the diversity of the test sets, we compute the average fraction of common samples between two tests sets created with different seeds. The value is in the range from 0 to 1, where 1

Figure 2. Joint distribution of target and confound. The first column presents the scatter plot of age and motion variable for CamCan (top) and UKBB (bottom). The second column shows the case of fluid intelligence prediction with age as confound for CamCan. In all cases, the target is clearly associated with the confound; the corresponding p-values are below 10^{-5}.

Table 2. Comparisons on population-imaging data, Camcan Fluid Intelligence prediction.

Method	MAE $\pm\sigma$	MAE $\pm\sigma$ permuted	p-value
CamCan: Age prediction			
Without deconfounding	6.17 ± 0.43	16.0 ± 1.24	0
Deconfounding test and train jointly	6.76 ± 0.53	16.24 ± 0.66	0
Out-of-sample deconfounding	9.3 ± 0.8	15.79 ± 1.22	0
Confound-isolating cross-validation	6.49 ± 0.46	15.21 ± 1.37	0
Prediction from confounds	13.74 ± 1.5	15.22 ± 1.74	0
CamCan: Fluid Intelligence prediction			
Without deconfounding	4.1 ± 0.29	6.05 ± 0.49	0
Deconfounding test and train jointly	4.08 ± 0.22	5.7 ± 0.34	0
Out-of-sample deconfounding	5.59 ± 0.32	6.04 ± 0.9	0.23
Confound-isolating cross-validation	4.31 ± 0.29	4.6 ± 0.3	0.06
Prediction from confounds	4.03 ± 0.6	5.7 ± 0.95	0
UKBB: Age prediction			
Without deconfounding	4.82 ± 0.4	6.95 ± 0.8	0
Deconfounding test and train jointly	4.95 ± 0.4	6.92 ± 0.8	0
Out-of-sample deconfounding	8.23 ± 0.33	7.12 ± 0.34	1
Confound-isolating cross-validation	5.08 ± 0.3	7.26 ± 0.6	0
Prediction from confounds	6.24 ± 0.73	6.29 ± 0.72	1
Tabular data: Income prediction			
Without deconfounding	0.79 ± 0.014	0.93 ± 0.016	0
Deconfounding test and train jointly	0.79 ± 0.014	0.93 ± 0.016	0
Out-of-sample deconfounding	0.77 ± 0.014	0.93 ± 0.016	0
Confound-isolating cross-validation	0.85 ± 0.13	0.94 ± 0.18	1
Prediction from confounds	0.87 ± 0.016	0.93 ± 0.016	0

Figure 3. Comparisons on simulated data. The left column of each sub-figure reports the prediction performance as the mean absolute error for the five approaches considered: Prediction from the data without deconfounding, prediction after deconfounding test and train jointly, prediction with out-of-sample deconfounding, prediction with confound-isolating cross-validation, and prediction from confounds. The left column displays the distribution across validation folds for the actual data (top, orange), and for permuted data distribution (bottom, gray). The right column displays the distribution of p-values across folds, obtained by permutation, and the text yields the aggregated p-value across folds, testing whether prediction accuracy is better than chance. Test subsets always represent 1/5 of the whole dataset. There are three simulation settings: (a) No direct link between target and brain, (b) A direct link between target and brain in the presence of a confound and (c) A weak confound and a direct link between target and brain. Green ticks indicate correct conclusions, red crosses mark incorrect ones, and warning signs the weak results.

Figure 4. Evolution of the test set created by Confound-isolating cross-validation. The joint distribution of the target (Fluid intelligence) and the confound (Age) for the CamCan dataset is taken for demonstration. We show the process of selecting proper samples for the test set. We begin with the entire dataset, Pearson correlation is −0.67 with infinitesimal p-value (right subplot). After half of the iterations we have already reached a correlation −0.17 with p-value = 0.009 (middle subplot). The final test set is shown on the right subplot, correlation −0.007 with p-value = 0.02. It presents negligible residual dependency between targets and confounds.

Figure 5. Evolution of the link between confound and target with the number of subjects for different subsampling methods on the CamCan dataset, considering age prediction. Applying Algorithm 3 effectively reduces statistical dependences between confound and target (red curve). In our experiments, we stop the sampling when the test set size is 1/5 of the dataset.

means that all test sets contain the same samples and 0 that test sets have no sample in common; the expected value is $\frac{1}{5}$. We find an average intersection of 0.30 for age prediction with CamCan and 0.27 with UKBB; for Fluid Intelligence prediction with CamCan, we find 0.36. This demonstrates that the test sets do not repeat much, hence that there is no hidden determinism in the cross-validation scheme of the proposed method.

Testing for confounded prediction

Figure 6 and Table 2 report the mean absolute error[2] for the different approaches to control for confounds. The figure also reports the p-value of predictive accuracy, from permutations[3]. The first thing to note is that without controlling for confounding effects, all models lead to significant prediction. But are these driven by the confounds? Given that the various approaches measure predictions on different data, we compare how far these predictions are above chance, rather than their absolute value.

Deconfounding test and train sets jointly –removing the linear effect of the confounding variable on the full data– has little impact on the prediction performance on all datasets. On the other hand, *out-of-sample deconfounding* significantly changes prediction performance in a way that varies across tasks. Prediction accuracy of fluid intelligence on CamCan falls to chance level. Age prediction on CamCan is little impacted. However, Age prediction on UKBB gives results worse than chance, i.e. worse than a model that learns to predict age on data where this relationship has been shuffled by permutation (see Figure 6 and Table 2). *Confound-isolating cross-validation* also gives varying results on different datasets. For fluid-intelligence prediction on CamCan, it also gives results at chance level. For age prediction on CamCan, it does alter significantly prediction accuracy, and on UKBB, it leads to a slightly worse prediction, but still above chance. Finally, *Prediction from confounds* leads to chance-level or good prediction of the target depending on the dataset. In particular, it does better than chance for Fluid intelligence prediction.

These results show that in all these datasets, the confounds **z** are associated with both the data **X** and the target **y**. For fluid intelligence prediction on CamCan, all the prediction of **y** from **X** is mediated by **z**. However, for age prediction in CamCan, there exists within **X** some signal that is unrelated to **z** but predicts **y**. Age prediction in UKBB is a more subtle situation: **X** contains signals from **z** and **y** with shared variance, but there is enough signal beyond the effect of **z** to achieve a good prediction, as demonstrated by *confound-isolating cross-validation*, where the prediction cannot be driven by **z**. Yet, out-of-sample deconfounding removes the shared variance and hence creates predictions that are worse than chance.

2 Mean absolute error is a good metric to compare across different test sets as it gives an absolute error measure in the unit of **y**, unlike explained variance, that depends on the variance of **y**.

3 Technically, there is one p-value per fold; to report only one number, we use p-value aggregation [60].

Tabular data

Figure 7 and Table 2 give the results of analysis on the tabular data. There is a significant prediction of income from socio-demographic and mental-health information, without any deconfounding. However, prediction from confounds shows that qualifications also predict income well. To control for qualification, deconfounding removes the signal explained by these in **X**. Here, deconfounding does not make the prediction worse; actually out-of-sample deconfounding improves it. Such an improvement can be explained if the deconfounding adds information about the confound to the signal rather than removing it, as can happen when the model of the confounds is mis-specified. To limit mis-specification issues, a random forest as the g function in algorithm 2. Finally, confound-isolating cross-validation shows very variable results, but overall that prediction does not work better than chance on balanced datasets, so that qualification is no longer specifically related to income.

Here, deconfounding leads to the conclusion that the prediction of income from social-demographic and mental-health information is not at all driven by qualifications while the other approaches suggest otherwise. The discrepancy is probably due to the complex non-linear interactions between these variables. The reality is probably that qualifications contribute to the prediction of income, as well as mental health and socio-demographics information, and that teasing out these contributions is hard.

Discussion and conclusion

Measuring the accuracy of predictive models, *eg* for biomarkers or brain decoding, must account for the presence of confounding effects that can contribute to the prediction. Indeed, an imaging biomarker that solely picks up head motion may detect pathologies with some success, but be overall a waste of scanner time. An accurate prediction of fluid intelligence from brain functional connectivity might simply be a consequence of indirectly capturing the subjects' age. Standard cross-validation procedures ignoring the confounds can overestimate prediction accuracy.

Addressing confounds in predictive modeling

Approaches must be adapted to out-of-sample settings

Deconfounding approaches used in standard GLM-based analysis must be adapted to out-of-sample data by separating estimation of the confounds' model from removal of the effect of confounds on the data, as detailed in section and algorithm 1. Importantly, applying deconfounding to the whole data without separating train and test set is not only wrong in theory –because it breaks independence of train and test data– but also leads to incorrect conclusions in practice, as clearly visible from the simulations.

Even done right, deconfounding in predictive settings can lead to pessimistic evaluations, as stressed by [26] and shown in our experiments. This is because the signal explained by the confound is removed from the brain signal before it is passed to the predictive model. The corresponding correction can remove too much information when there a large amount of shared signal between the confound and the target –eg aging and Alzheimer's disease. Such problem does not arise in a GLM-based standard analysis because the confounds and the effects of interest are modeled *simultaneously*, and the consequences of shared signal are easier to handle.

To give a measure of predictive accuracy that is not pessimistic, we also study a different approach: testing the predictive model on a subset of the data crafted such that the effect of interest is independent from the confound. When the confounding effect is represented as a categorical variable, for instance the effect of acquisition site, the approach can be simple as it amounts to splitting the data so as to ensure that generalization occurs for a category not observed in the training set. Creating an adequate test set for continuous confounds requires a dedicated method, as with *confound-isolating cross-validation* (Algorithm 3). It enables a test of the predictive power from brain imaging without discarding the potentially useful shared signal. In addition, it is non-parametric and does not rely on a linear confounding model. Empirical studies, on both brain-imaging data and simulations, show that both out-of-sample deconfounding and *confound-isolating cross-validation* can control correctly for confounds. Deconfounding before fitting a predictive model brings the benefit of building a predictor free of the confounding effect. However, it can remove shared variance and lead to pessimistic evaluations. *Confound-isolating cross-validation* brings the benefit of measuring the predictive power in the absence of the confounding effect. Such measure is of direct importance to gauge the practical value of a biomarker. As an attractive complementary approach, note that deep learning approaches for learning confound-free models have been proposed in [61].

To summarize, our main claim is that it is possible to learn a confounded model yet evaluate it in an unbiased fashion. What matters in this logic is that the predictive accuracy after *confound-isolating cross-validation* remains better than chance, which amounts to performing an omnibus test of the variables of the model. The case where *confound-isolating cross-validation* would yield a null result certainly means that one should be cautious in claiming a conditional association between X and y, as slight variations in the confounding model may render the association significant or not: indeed the apparent association between features and target is dominated by the confounder and thus, not a reliable one. In brief, this has an impact on the practical significance of claimed associations.

Which approach to use when: deconfounding versus confound-isolating cross-validation

Out-sample deconfounding and *confound-isolating cross-validation* give valid and complementary information. In the worst case, these approaches can be conservative, but they don't yield spurious associations. From a prediction perspective, when the training population reflects adequately the target population, changing the training data to remove the effect of the confounder may not improve prediction accuracy [24]. For instance, for many pathologies, patients move more in the scanner than healthy individuals. Should an imaging-biomarker of the pathology be developed, this effect will be most likely true in the population on which the biomarker is applied. Hence it is counter-productive to force the biomarker to discard this information. Rather, *confound-isolating cross-validation* should be used to check that the imaging biomarker does bring in value in addition to capturing motion.

On the other hand, *confound-isolating cross-validation* is not a universal remedy: removing a confounding effect from the training data may be useful if the confounder is incidentally correlated with $\mathbf{X}$ or $\mathbf{y}$ without any clear causal relationship. This is the case if the confounder is a feature of the measurement process. For instance, if the data are acquired across two imaging sites with different scanners, but one site recruited a much larger fraction of patients than the other, the risk is that the predictor learns to use information about the scanner rather than the pathology. In such a case, the training strategy must be adapted, for instance by removing the effect of the confound.

Finally, if the goal is to *interpret* successful prediction as evidence of a link between brain signals and the predicted outcome, modifying the training data is more likely to disentangle the biomarker pattern of interest from the confounding effect. In such a situation, deconfounding should be preferred, to give a model, with its parameters, that is not driven by the confounding signal.

Limitation: with many confounds the problem is harder

Here we have studied the case of one, clearly-identified, confound. The case of multiple confounds (*eg* age, education, gender, ethnicity), is more challenging. In such situations, deconfounding approaches may remove fully the signal of interest. For *confound-isolating cross-validation*, reliable estimation of mutual information will require much larger sample sizes than with a single confound. In practice, we recommend to identify the most impactful confound to run *confound-isolating cross-validation*.

Another concern could be that such confounding factors are not well identified. In that case, the proposed approach does

Figure 6. Comparisons on population-imaging data Each sub-figure shows one prediction setting: (a) CamCan Age prediction, (b) CamCan Fluid Intelligence prediction, (c) UKBB Age prediction. The left column of each sub-figure reports the prediction performance as the mean absolute error for the five approaches considered: Prediction from the data without deconfounding, prediction after deconfounding test and train jointly, prediction with out-of-sample deconfounding, prediction with confound-isolating cross-validation, and prediction from confounds. The left column displays the distribution across validation folds for the actual data (top, orange), and for permuted data distribution (bottom, gray). The right column displays the distribution of p-values across folds, obtained by permutation, and the text yields the aggregated p-value across folds (see the main text), testing whether prediction accuracy is better than chance. Test subsets always represent 1/5 of the whole dataset. The figure clearly displays different behaviors across the three problems: without deconfounding, and deconfounding test and train jointly yield significant, but probably spurious accuracy; out-of-sample deconfounding can be over-conservative (the prediction is worse than chance on UKBB) suggesting that the deconfounding model removes too much variance; confound-isolating cross validation yields more nuanced results, and prediction from confounds yields variable results.

not help, but such a case is very hard to handle with statistical methods (see e.g. [62]). We thus leave handling of imperfect confounder knowledge for future research.

Elements to interpret analyses with confounds

Defining confounds calls for modeling choices

Whether a variable should be considered as a confounding effect or not is not dictated by the data, but by the question at hand. The actual notion of confound comes from causal modeling, to give a causal interpretation to model parameters [15, 63]. Confound variables are then chosen so as to model the difference between the measurements at hand and those obtained with a hypothetical intervention. Such choices are implicitly based on a model of which variables are causes or consequences of the fictional intervention and the outcome of interest [see 64, for guidelines in the case of UKBB].

In pure biomarker settings, the focus is not on potential interventions, but on detecting or predicting an outcome. The concern is then that the measured accuracy might not reflect the actual application settings [27, 22]. Here also, the choice of variables to control for must be governed by an understanding of how the data at hand may differ from ideal data to reflect the target application. More concretely, Confounds can indeed relate to any aspect of the setup, e.g. acquisition devices, data processing routines when these are not homogeneous across all the dataset, measurement-related covariate such as motion, individual conditions, such as age, sex or genetics, that is correlated with the imaging variable and with the outcome.

Deconfounding for causal interpretations: the collider-bias danger

Using deconfounding to cancel the impact of a putative confound $\mathbf{z}$ removes any bias incurred by the spurious association between the data $\mathbf{X}$ and the prediction target $\mathbf{y}$, when $\mathbf{z}$ is associated with both $\mathbf{X}$ and $\mathbf{y}$. However, $\mathbf{z}$ may be a *consequence* of both the target and the data. In such a situation conditioning on it can create a form of selection bias, sometimes known as "collider bias" [65, 66]. Conditioning on the third variable $\mathbf{z}$ can then reverse the correlation between two variables $\mathbf{X}$ and $\mathbf{y}$, a phenomenon known as Berkson's or Simpson's statistical paradox [67, 68]. It can be understood from a simple example: when studying a population of hospital patients, individuals may have been admitted to the hospital because they have disease A or B. On this specific population, the two diseases are anti-correlated. However, concluding that disease A protects from disease B would be incorrect. Another example can be found in a cognitive experiment where both a visible-enough stimuli and a timely motor response are needed for a successful response. When learning a model decoding stimuli visibility from brain response, deconfounding on successful responses would lead this model to rely on motor-cortex activity, while the link between visual stimuli and motor cortex is not neuroscientifically relevant as such. Deconfounding by itself does not suffice to yield associations with clear interpretations.

Figure 7. Comparisons on tabular data: predicting income from socio-demographics and mental-health, controlling for qualifications. The left column of the figure reports the prediction performance by the mean absolute error for the five approaches considered: Prediction from the data without deconfounding, prediction after deconfounding test and train jointly, prediction with out-of-sample deconfounding, prediction with confound-isolating cross-validation, and prediction from confounds. The left column displays the distribution across validation folds for the actual data (top, orange), and for permuted data distribution (bottom, gray). The right column displays the distribution of p-values across folds, obtained by permutation, and the text yields the aggregated p-value across folds (see the main text), testing whether prediction accuracy is better than chance.

A sampling view on confounds

Confound-isolating cross-validation strives to sample an ideal sub-population. This is also one of the best strategies to avoid the presence of confounds in experimental settings: targeting the recruitment of participants so that the design is balanced, for instance with matched controls or randomized controlled trials. But this can only be done at study design, and targeted acquisitions, with matching and restriction, can make it hard to collect large samples or tackle many covariates. At analysis time, researchers have to rely on statistical methods to adapt the analysis to the presence of confounds. For in-sample analysis, propensity scores are a classic reweighting technique used to obtain reliable effect estimates from confounded datasets [69, 70]. The use of subsampling in *confound-isolating cross-validation* can be seen as an extension of these ideas for out-sample validation of predictive accuracy. The only caveat is that one has to ensure that sampling does not deterministically lead to a fixed test set, which would weaken the statistical guarantees brought by the validation experiment. Here, we propose to perform this check a posteriori. In the future, more complex sampling strategies could be designed to ensure some randomness in the test set.

Conclusion: deconfounding and isolating confounds are complementary

Deconfounding strives to remove confounding effects from the data, after which successful prediction can be interpreted as a direct link from the remaining brain signals to the outcome of interest. However, in biomarkers settings, the primary focus may be on the quality of detection, rather than interpretation, for instance to improve diagnosis or prognosis. In such settings, an important question is: how much do the brain signals improve the prediction upon a simpler measure of the confounding effect? Answering this question calls for a cross-validation procedure isolating this confounding effect. The corresponding prediction accuracy can then safely be interpreted as not resulting in any way from the confounding effect.

Availability of Supporting Source code and Requirements

- Project name: Confound Prediction
- Project home page: https://github.com/darya-chyzhyk/confound_prediction
- Operating system(s): OS independent
- Programming language: Python
- Other requirements: Python (>= 3.5), Scipy (>=1.1.0), Scikit-learn (>=0.21.2), Numpy (>=1.14.2), Pytest(>=5.1.1)
- License: BSD 3-Clause License

Data Availability

Supporting data and Python implementation to control confound effect are available via the GigaScience database GigaDB [71]

Acknowledgment

This work was funded by the Child Mind Institute Resting state MRI data analysis was done using the UK Biobank Resource under project 25163. We show appreciation to the UK Biobank contributors and participants for collecting and sharing the quality data to researchers. BT was also supported by the SC1-DTH-07-2018 H2020 VirtualBrainCloud Project under grant agreement

No 826421, and GV by the DirtyData (ANR-17-CE23-0018-01) project.

References

1. Norman KA, Polyn SM, Detre GJ, Haxby JV. Beyond mind-reading: multi-voxel pattern analysis of fMRI data. Trends in cognitive sciences 2006;10:424.
2. Poldrack RA. Inferring mental states from neuroimaging data: from reverse inference to large-scale decoding. Neuron 2011;72:692.
3. Varoquaux G, Poldrack RA. Predictive models avoid excessive reductionism in cognitive neuroimaging. Current opinion in neurobiology 2019;55:1–6.
4. Plant C, Teipel SJ, Oswald A, Böhm C, Meindl T, Mourao-Miranda J, et al. Automated detection of brain atrophy patterns based on MRI for the prediction of Alzheimer's disease. Neuroimage 2010;50(1):162–174.
5. Wager TD, Atlas LY, Lindquist MA, Roy M, Woo CW, Kross E. An fMRI-based neurologic signature of physical pain. New England Journal of Medicine 2013;368:1388.
6. Abraham A, Milham MP, Di Martino A, Craddock RC, Samaras D, Thirion B, et al. Deriving reproducible biomarkers from multi-site resting-state data: An Autism-based example. NeuroImage 2017;147:736–745.
7. Dosenbach NU, Nardos B, Cohen AL, Fair DA, Power JD, Church JA, et al. Prediction of individual brain maturity using fMRI. Science 2010;329(5997):1358–1361.
8. Liem F, Varoquaux G, Kynast J, et al. Predicting brain-age from multimodal imaging data captures cognitive impairment. NeuroImage 2017;148:179.
9. Woo CW, Chang LJ, Lindquist MA, Wager TD. Building better biomarkers: brain models in translational neuroimaging. Nature neuroscience 2017;20(3):365–377.
10. Engemann DA, Kozynets O, Sabbagh D, Lemaître G, Varoquaux G, Liem F, et al. Combining magnetoencephalography with magnetic resonance imaging enhances learning of surrogate-biomarkers. eLife 2020;9.
11. Cole JH, et al. Brain age predicts mortality. Molecular Psychiatry 2018;23(5):1385–1392.
12. Power JD, Barnes KA, Snyder AZ, Schlaggar BL, Petersen SE. Spurious but systematic correlations in functional connectivity MRI networks arise from subject motion. Neuroimage 2011;59:2142.
13. Miller KL, Alfaro-Almagro F, Bangerter NK, Thomas DL, Yacoub E, Xu J, et al. Multimodal population brain imaging in the UK Biobank prospective epidemiological study. Nature Neuroscience 2016;.
14. Smith SM, Nichols TE. Statistical Challenges in "Big Data" Human Neuroimaging. Neuron 2018;97(2):263 – 268.
15. Pearl J. Causality: Models, Reasoning, and Inference. New York, NY, USA: Cambridge University Press; 2000.
16. Worsley K, Liao C, Aston J, Petre V, Duncan G, Morales F, et al. A general statistical analysis for fMRI data. NeuroImage 2002;15:1.
17. Poldrack RA, Mumford JA, Nichols TE. Handbook of functional MRI data analysis. Cambridge: University Press; 2011.
18. Breiman L. Statistical Modeling: The Two Cultures (with comments and a rejoinder by the author). Statistical Science 2001;16(3):199 – 231. https://doi.org/10.1214/ss/1009213726.
19. Poldrack RA, Huckins G, Varoquaux G. Establishment of best practices for evidence for prediction: a review. JAMA psychiatry 2020;77(5):534–540.
20. Chyzhyk D, Varoquaux G, Thirion B, Milham M. Controlling a confound in predictive models with a test set minimizing its effect. In: PRNI 2018 - 8th International Workshop on Pattern Recognition in Neuroimaging Singapore, Singapore; 2018. p. 1–4. https://hal.archives-ouvertes.fr/hal-01831701.
21. Varoquaux G, Raamana PR, Engemann DA, Hoyos-Idrobo A, Schwartz Y, Thirion B. Assessing and tuning brain decoders: cross-validation, caveats, and guidelines. NeuroImage 2017;145:166–179.
22. Little MA, Varoquaux G, Saeb S, Lonini L, Jayaraman A, Mohr DC, et al. Using and understanding cross-validation strategies. Perspectives on Saeb et al. GigaScience 2017;6:1.
23. Linn KA, Gaonkar B, Doshi J, Davatzikos C, Shinohara RT. Addressing confounding in predictive models with an application to neuroimaging. The international journal of biostatistics 2016;12(1):31–44.
24. Rao A, Monteiro JM, Mourao-Miranda J, Initiative AD, et al. Predictive modelling using neuroimaging data in the presence of confounds. NeuroImage 2017;150:23–49.
25. Friston KJ, Holmes AP, Worsley KJ, Poline JB, Frith C, Frackowiak RSJ. Statistical Parametric Maps in Functional Imaging: A General Linear Approach. Hum Brain Mapp 1995;p. 189.
26. Snoek L, Miletic S, Scholte HS. How to control for confounds in decoding analyses of neuroimaging data. bioRxiv 2018;.
27. Saeb S, Lonini L, Jayaraman A, Mohr DC, Kording KP. The need to approximate the use-case in clinical machine learning. Gigascience 2017;6(5):1–9.
28. Arjovsky M, Bottou L, Gulrajani I, Lopez-Paz D, Invariant Risk Minimization; 2020.
29. Devroye L. Non-Uniform Random Variate Generation(originally published with. Springer-Verlag; 1986. http://cg.scs.carleton.ca/~luc/rnbookindex.html.
30. Devroye L. Non-Uniform Random Variate Generation. Springer-Verlag; 1986.
31. Chyzhyk D. Confound Prediction. GitHub repository 2019;https://github.com/darya-chyzhyk/confound_prediction.
32. Chyzhyk D. Confound Prediction. Python package version 0.0.1a1. The Python Package Index (PyPI) repository 2019;https://pypi.org/project/confound-prediction/.
33. Long X, Benischek A, Dewey D, Lebel C. Age-related functional brain changes in young children. NeuroImage 2017;155:322–330.
34. Zepf FD, Bubenzer-Busch S, Runions KC, Rao P, Wong JWY, Mahfouda S, et al. Functional connectivity of the vigilant-attention network in children and adolescents with attention-deficit/hyperactivity disorder. Brain and Cognition 2017;.
35. Li H, Satterthwaite TD, Fan Y. Brain age prediction based on resting-state functional connectivity patterns using convolutional neural networks. 2018 IEEE 15th International Symposium on Biomedical Imaging (ISBI 2018) 2018;p. 101–104.
36. Franke K, Ziegler G, Klöppel S, Gaser C, Initiative ADN, et al. Estimating the age of healthy subjects from T1-weighted MRI scans using kernel methods: exploring the influence of various parameters. Neuroimage 2010;50(3):883–892.
37. Geerligs L, Tsvetanov K, Cam-CAN, Henson R. Challenges in measuring individual differences in functional connectivity using fMRI: The case of healthy aging. Human Brain Mapping 2017;38(8):4125–4156.
38. Yan CG, Cheung B, Kelly C, Colcombe S, Craddock RC, Martino AD, et al. A comprehensive assessment of regional variation in the impact of head micromovements on functional connectomics. NeuroImage 2013;76:183–201.
39. Satterthwaite TD, Wolf DH, Loughead J, Ruparel K, Elliott

MA, Hakonarson H, et al. Impact of in-scanner head motion on multiple measures of functional connectivity: Relevance for studies of neurodevelopment in youth. NeuroImage 2012;60(1):623 – 632.
40. Satterthwaite TD, Elliott MA, Gerraty RT, Ruparel K, Loughead J, Calkins ME, et al. An Improved Framework for Confound Regression and Filtering for Control of Motion Artifact in the Preprocessing of Resting-State Functional Connectivity Data. NeuroImage 2012;.
41. Gilmore A, Buser N, Hanson JL. Variations in Structural MRI Quality Impact Measures of Brain Anatomy: Relations with Age and Other Sociodemographic Variables. bioRxiv 2019;.
42. Van Dijk KRA, Sabuncu MR, Buckner RL. The influence of head motion on intrinsic functional connectivity MRI. Neuroimage 2012;59:431.
43. Finn ES, Shen X, Scheinost D, Rosenberg MD, Huang J, Chun MM, et al. Functional connectome fingerprinting: identifying individuals using patterns of brain connectivity. Nature Neuroscience 2015 oct;18(11):1664–1671.
44. Hearne LJ, Mattingley JB, Cocchi L. Functional brain networks related to individual differences in human intelligence at rest. In: Scientific reports; 2016. .
45. Cattell RB. Abilities : their structure, growth, and action. Houghton Mifflin Boston; 1971.
46. Hartshorne JK, Germine LT. When does cognitive functioning peak? The asynchronous rise and fall of different cognitive abilities across the life span. Psychological science 2015;26:433.
47. Samu D, Campbell KL, Tsvetanov KA, Shafto MA, Cam-CAN consortium, Tyler LK. Preserved cognitive functions with age are determined by domain-dependent shifts in network responsivity. Nature communications 2017 May;8.
48. Bugg JM, Zook NA, DeLosh EL, Davalos DB, Davis HP. Age differences in fluid intelligence: Contributions of general slowing and frontal decline. Brain and Cognition 2006;62(1):9 – 16.
49. Rönnlund M, Pudas S. The neural determinants of age-related changes in fluid intelligence : a pre-registered , longitudinal analysis in UK Biobank; 2018. .
50. Horn JL, Cattell RB. Age differences in fluid and crystallized intelligence. Acta Psychologica 1967;26:107 – 129.
51. Taylor JR, Williams N, Cusack R, Auer T, Shafto MA, Dixon M, et al. The Cambridge Centre for Ageing and Neuroscience (Cam-CAN) data repository: Structural and functional MRI, MEG, and cognitive data from a cross-sectional adult lifespan sample. NeuroImage 2017 jan;144:262.
52. Sudlow C, et al. UK Biobank: An Open Access Resource for Identifying the Causes of a Wide Range of Complex Diseases of Middle and Old Age. PLOS Medicine 2015 03;12(3):1–10.
53. Smith S, Alfaro Almagro F, Miller K. UK Biobank Brain Imaging Documentation 2017;http://biobank.ctsu.ox.ac.uk/crystal/docs/brain_mri.pdf.
54. Alfaro-Almagro F, Jenkinson M, Bangerter NK, Andersson JLR, Griffanti L, Douaud G, et al. Image processing and Quality Control for the first 10,000 brain imaging datasets from UK Biobank. NeuroImage 2018;166:400 – 424.
55. Nichols TE, Das S, Eickhoff SB, Evans AC, Glatard T, Hanke M, et al. Best Practices in Data Analysis and Sharing in Neuroimaging using MRI. bioRxiv 2016;.
56. Dadi K, Rahim M, Abraham A, Chyzhyk D, Thirion B, Varoquaux G. Benchmarking functional connectome-based predictive models for resting-state fMRI. NeuroImage 2019;192:115–134.
57. Bellec P, Rosa-Neto P, Lyttelton OC, Benali H, Evans AC. Multi-level bootstrap analysis of stable clusters in resting-state fMRI. NeuroImage 2010;51:1126.
58. Varoquaux G, Baronnet F, Kleinschmidt A, Fillard P, Thirion B. Detection of brain functional-connectivity difference in post-stroke patients using group-level covariance modeling. In: MICCAI; 2010.
59. Abraham A, Pedregosa F, Eickenberg M, Gervais P, Mueller A, Kossaifi J, et al. Machine learning for neuroimaging with scikit-learn. Frontiers in Neuroinformatics 2014;8:14.
60. Meinshausen N, Meier L, Bühlmann P. P-values for high-dimensional regression. Journal of the American Statistical Association 2009;104(488):1671–1681.
61. Zhao Q, Adeli E, Pohl KM. Training confounder-free deep learning models for medical applications. Nature Communications 2020 Nov;11(1):6010. https://doi.org/10.1038/s41467-020-19784-9.
62. Brumback BA, Hernán MA, Haneuse SJ, Robins JM. Sensitivity analyses for unmeasured confounding assuming a marginal structural model for repeated measures. Stat Med 2004 Mar;23(5):749–767.
63. Angrist JD, Pischke JS. Mostly harmless econometrics: An empiricist's companion. Princeton university press; 2008.
64. Alfaro-Almagro F, McCarthy P, Afyouni S, Andersson JLR, Bastiani M, Miller KL, et al. Confound modelling in UK Biobank brain imaging. NeuroImage 2020;p. 117002. http://www.sciencedirect.com/science/article/pii/S1053811920304882.
65. Cole SR, Platt RW, Schisterman EF, Chu H, Westreich D, Richardson D, et al. Illustrating bias due to conditioning on a collider. International journal of epidemiology 2009;39(2):417–420.
66. Greenland S. Quantifying biases in causal models: classical confounding vs collider-stratification bias. Epidemiology 2003;14(3):300–306.
67. Berkson J. Limitations of the application of fourfold table analysis to hospital data. Biometrics Bulletin 1946;2(3):47–53.
68. Simpson EH. The interpretation of interaction in contingency tables. Journal of the Royal Statistical Society: Series B (Methodological) 1951;13(2):238–241.
69. Rubin DB. Estimating causal effects from large data sets using propensity scores. Annals of internal medicine 1997;127(8_Part_2):757–763.
70. Becker SO, Ichino A. Estimation of average treatment effects based on propensity scores. The stata journal 2002;2(4):358–377.
71. Chyzhyk D, Thirion B, Varoquaux G, Milham MP. Supporting data for "How to remove or control confounds in predictive models, with applications to brain biomarkers". GigaScience Database 2022;http://dx.doi.org/10.5524/100980.
72. Pypreprocess. GitHub repository 2019;https://github.com/neurospin/pypreprocess.
73. Gorgolewski K, Burns CD, Madison C, Clark D, Halchenko YO, Waskom ML, et al. Nipype: a flexible, lightweight and extensible neuroimaging data processing framework in python. Frontiers in neuroinformatics 2011;5:13.

Appendices

Data preprocessing

CamCan data were preprocessed using Pypreprocess [72], a collection of Python scripts for preprocessing fMRI data, that is based on the SPM12 software and the nipype toolbox [73]. We preprocessed CamCan data only. For UKBB data the preprocessed and connectivity matrices are available from the data repository. We apply a commonly used protocol that includes the following steps: Motion correction, correction for subject's head motion during the acquisition. Estimated six motion parameters (three translational parameters and three rotational parameters) are used as confounds in the age prediction experiments. For each subject we expressed the head motion using translation across all three axes as a square root of the mean of the sum of square finite difference of each translation axes over the time: $\sqrt{\frac{\vec{\Delta} translation_x^2 + \vec{\Delta} translation_y^2 + \vec{\Delta} translation_z^2}{3}}$
The rest-fMRI data are coregistered to the anatomical T1-MRI and then normalized to MNI template.

Supplementary results on the resting state data sets

Figure 8. Evolution of mutual information and correlation with the number of subjects for different subsampling methods on the CamCan dataset with Fluid Intelligence prediction and UKBB Age prediction. This figure shows that the proposed method effectively reduces statistical dependencies between confound and target (red curve) for both data sets and both predictors.

Supplementary results on simulated data, 1000 samples

Figure 9. Benchmarking approaches to control confounded predictions on simulated data with many samples. The left column of each sub-figure assesses the prediction performance through the mean absolute error (in signal units). We display the error distribution across validation folds for the data (top, orange), and for permuted data distribution (bottom, gray). The right column displays the distribution of p-values across folds, obtained by permutation, and the text reports the aggregated p-value across folds (see the main text). Five approaches are benchmarked: Without deconfounding, Deconfounding test and train jointly, Out-of-sampling deconfounding, Confound-isolating cross-validation, and Prediction from confounds. There are three simulation settings: (a) No direct link between target and brain, (b) A direct link between target and brain and (c) A weak confound and a direct link between target and brain. Green ticks indicate correct conclusions, red crosses mark incorrect ones, and warning signs the weak results.

Figure
Click here to access/download;Figure;figures_01.pdf

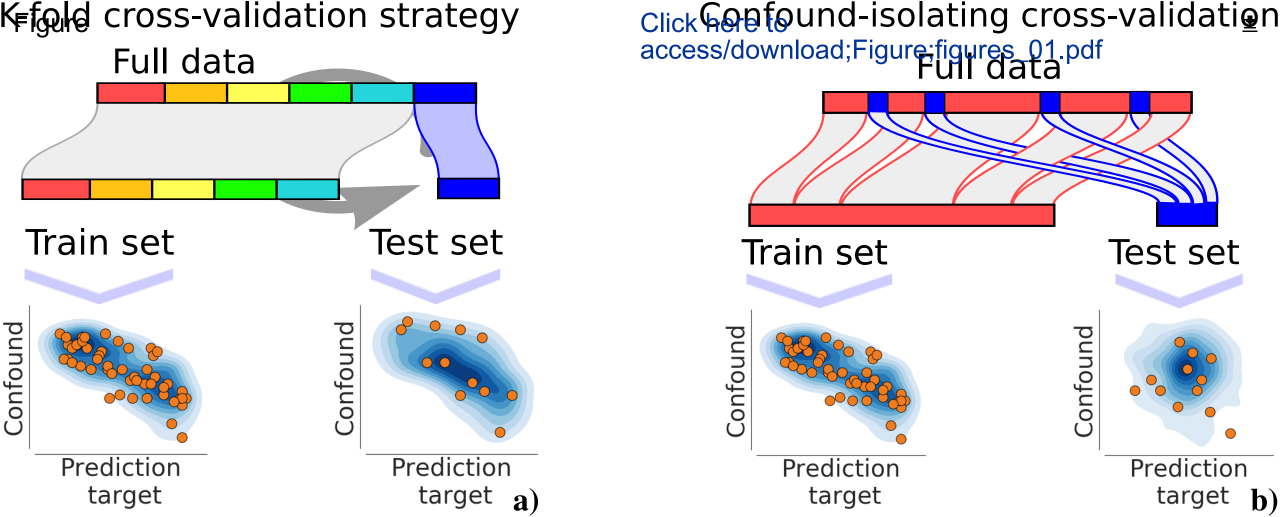

Figure
Click here to access/download;Figure;figur

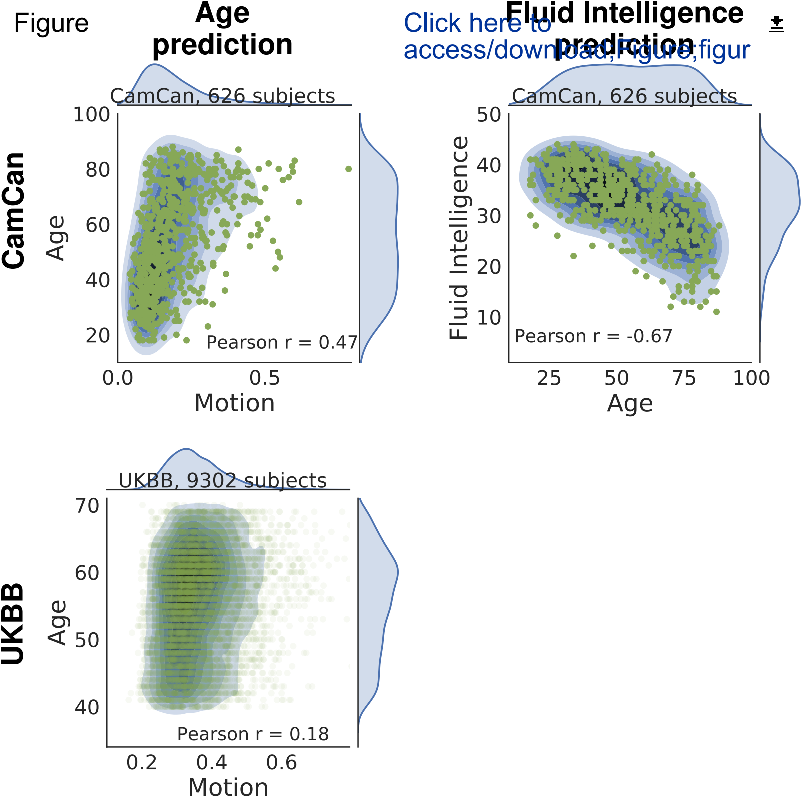

Figure

Click here to access/download;Figure;figures_04.pdf

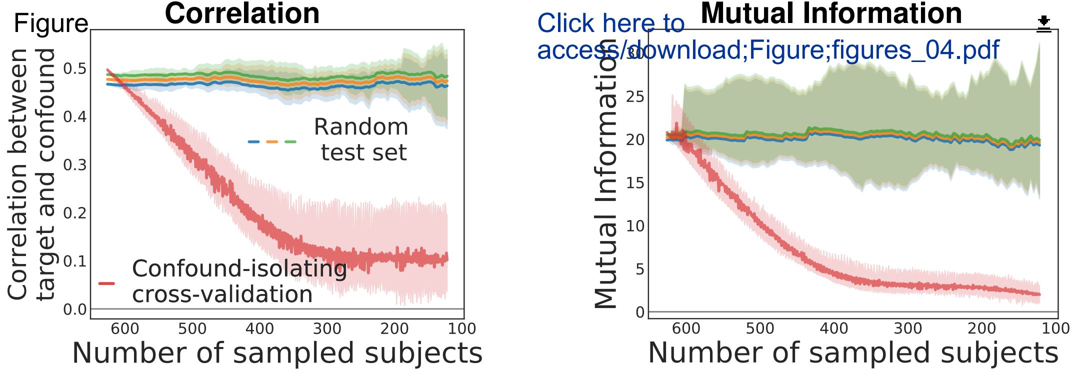

Figure

Click here to access/download;Figure;figures_05.pdf

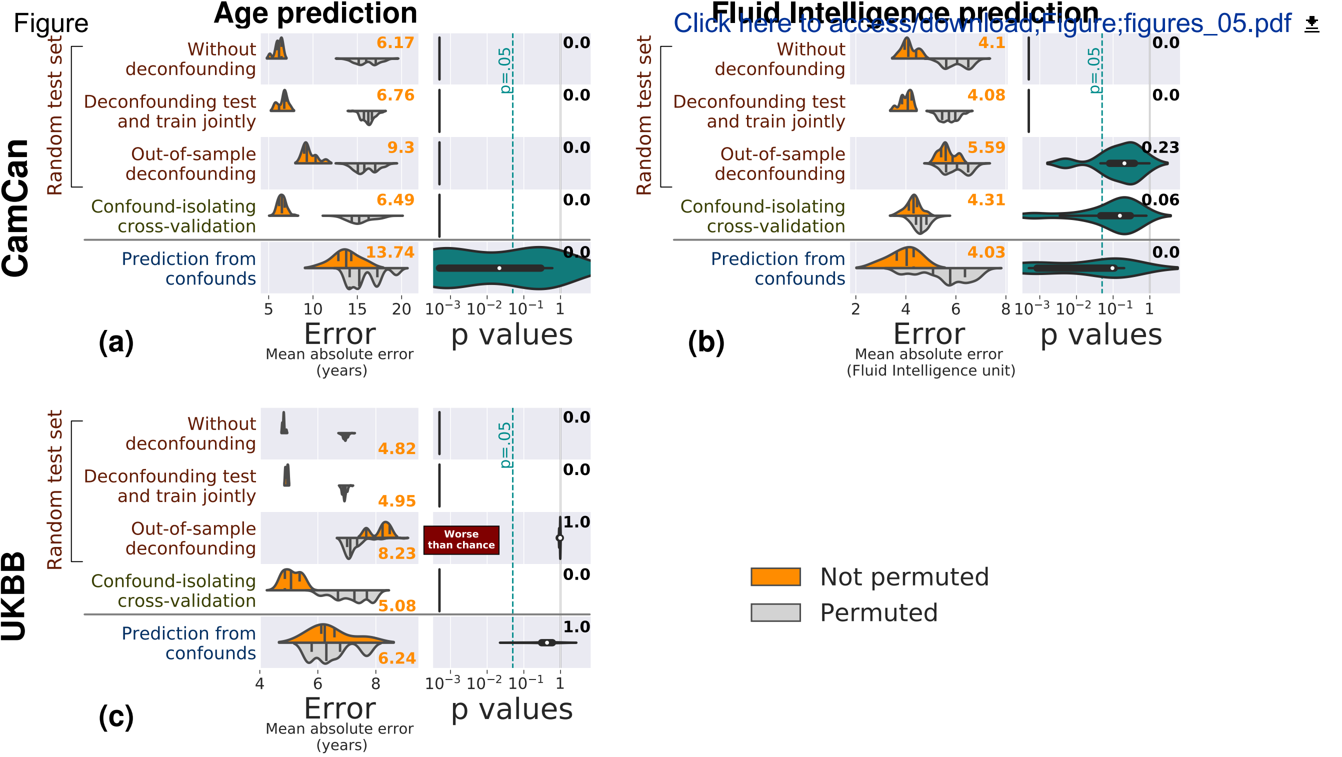

Figure

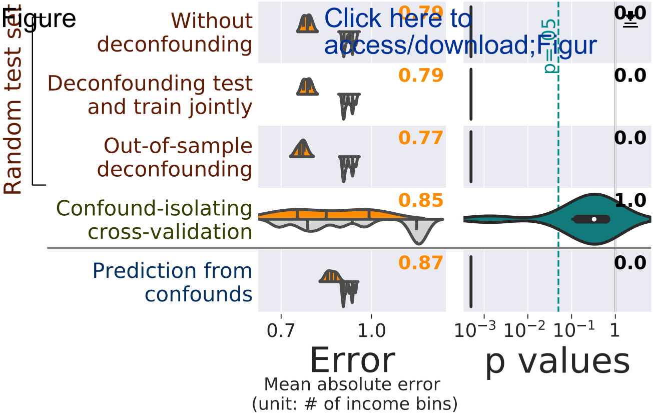
Click here to access/download;Figur
Random test set
Without deconfounding
0.79
0.0
Deconfounding test and train jointly
0.79
0.0
Out-of-sample deconfounding
0.77
0.0
Confound-isolating cross-validation
0.85
1.0
Prediction from confounds
0.87
0.0
p=.05
0.7
1.0
10^{-3}
10^{-2}
10^{-1}
1
Error
Mean absolute error (unit: # of income bins)
p values

Figure
Click here to access/download;Figure;figures_07.pdf

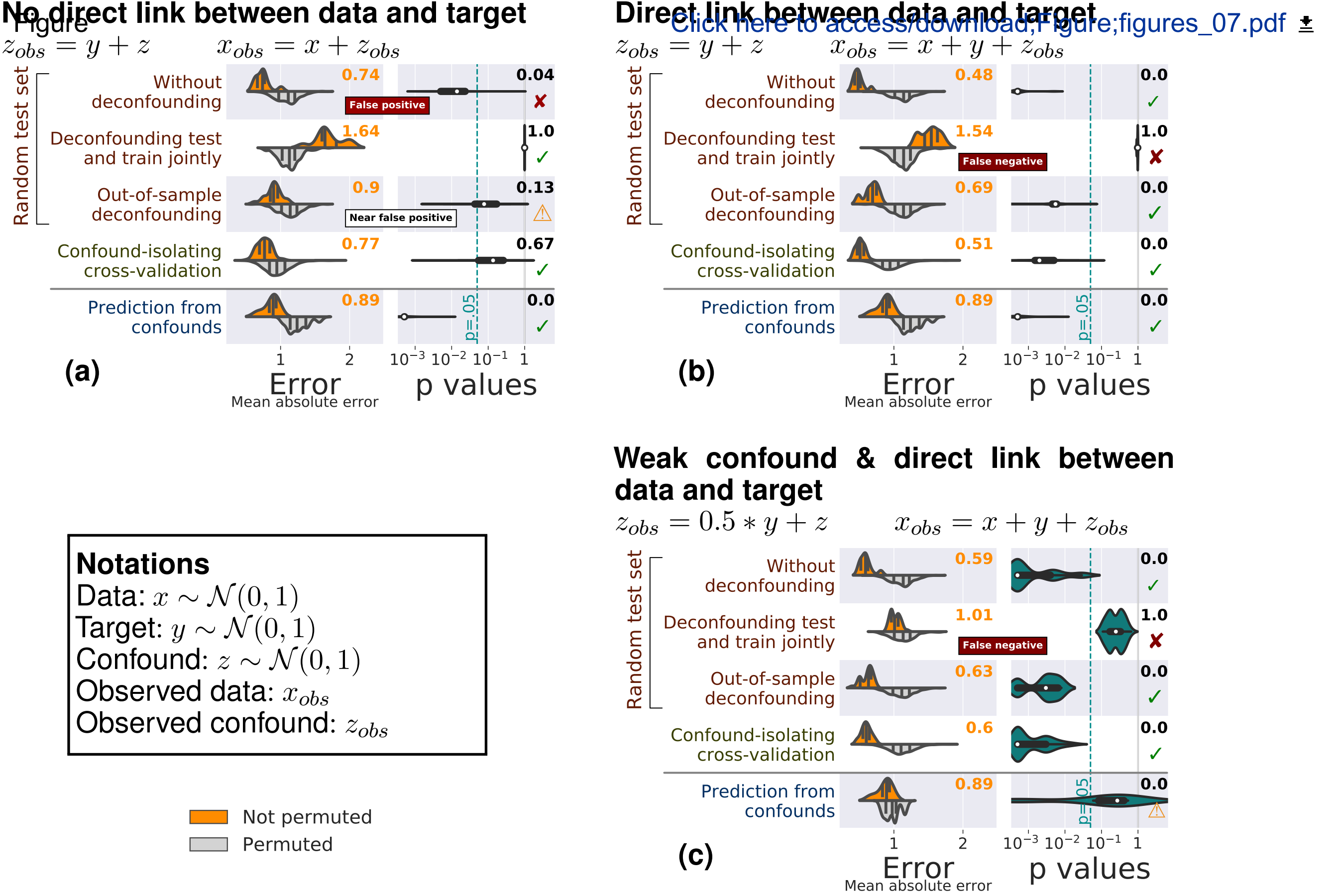

Figure
Click here to access/download;Figure;figures_08_Sup

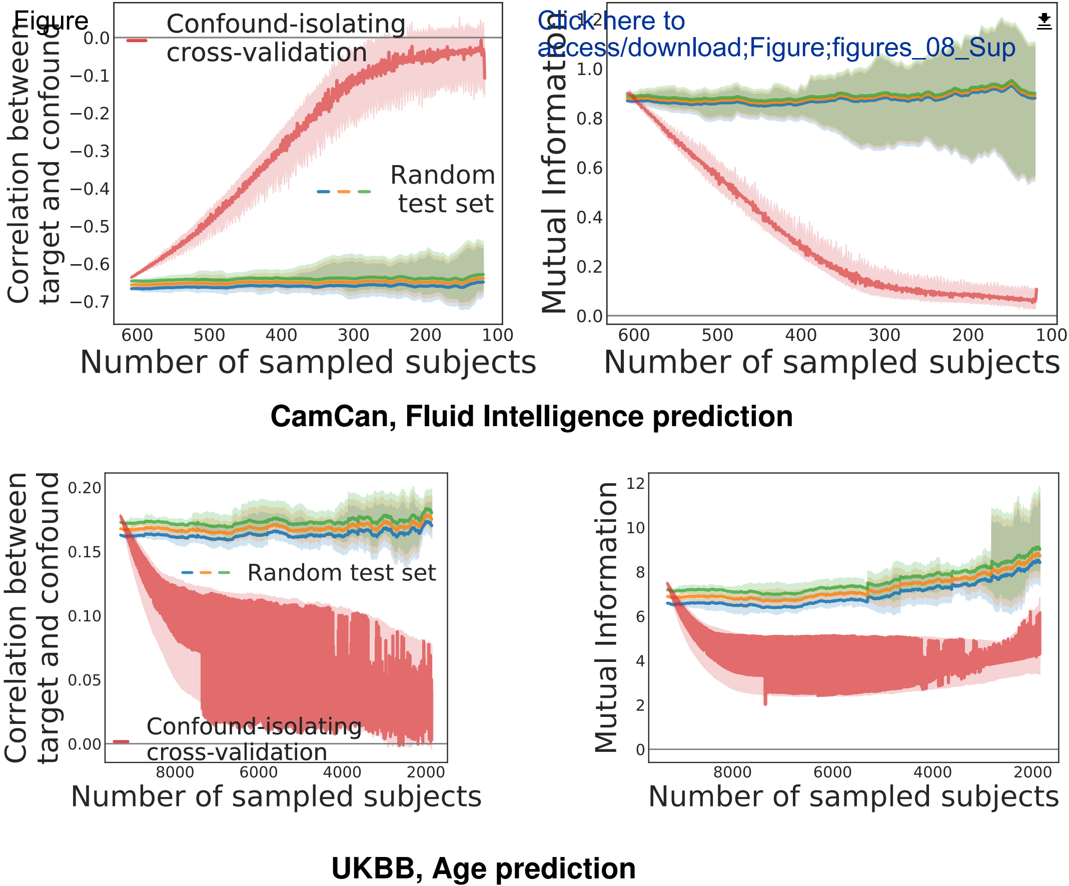

Figure
Click here to access/download;Figure;figures_09_Supplementary

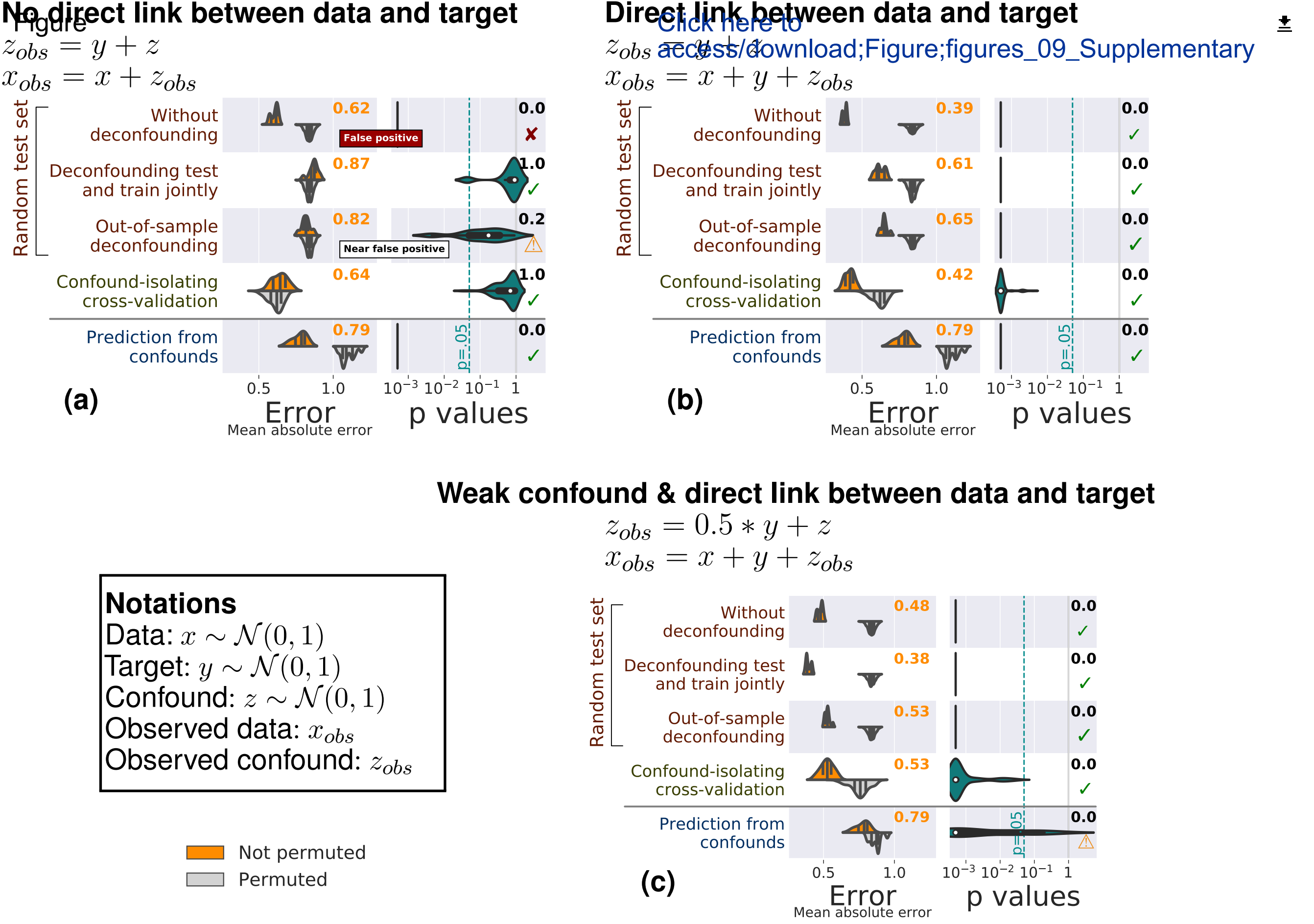

Figure

Click here to access/download;Figure;figures_03.pdf

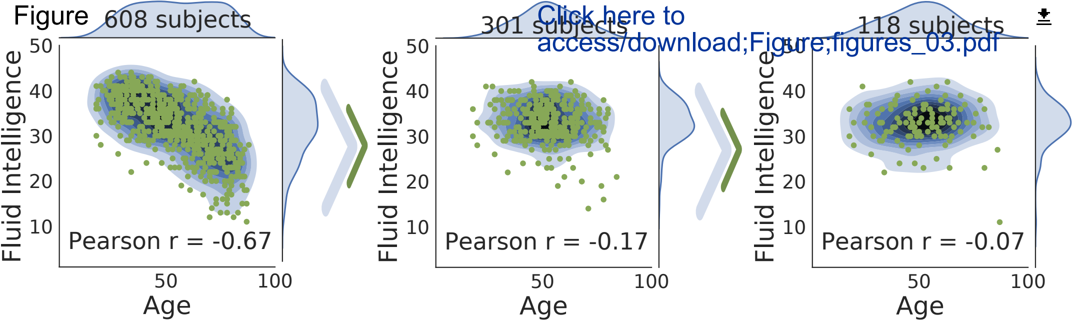

Supplement: giac014_GIGA-D-21-00125_Revision_3 [file giac014_giga-d-21-00125_revision_3.pdf]
