# Supplementary material for: How to remove or control confounds in predictive models, with applications to brain biomarkers
Source: Gigascience. 2022 Mar 12;11:giac014. doi: 10.1093/gigascience/giac014 (PMC8917515; doi:10.1093/gigascience/giac014)
Supplement: giac014_Supplemental_Files [file giac014_supplemental_files.zip › figures_09_Supplementary Material.pdf]

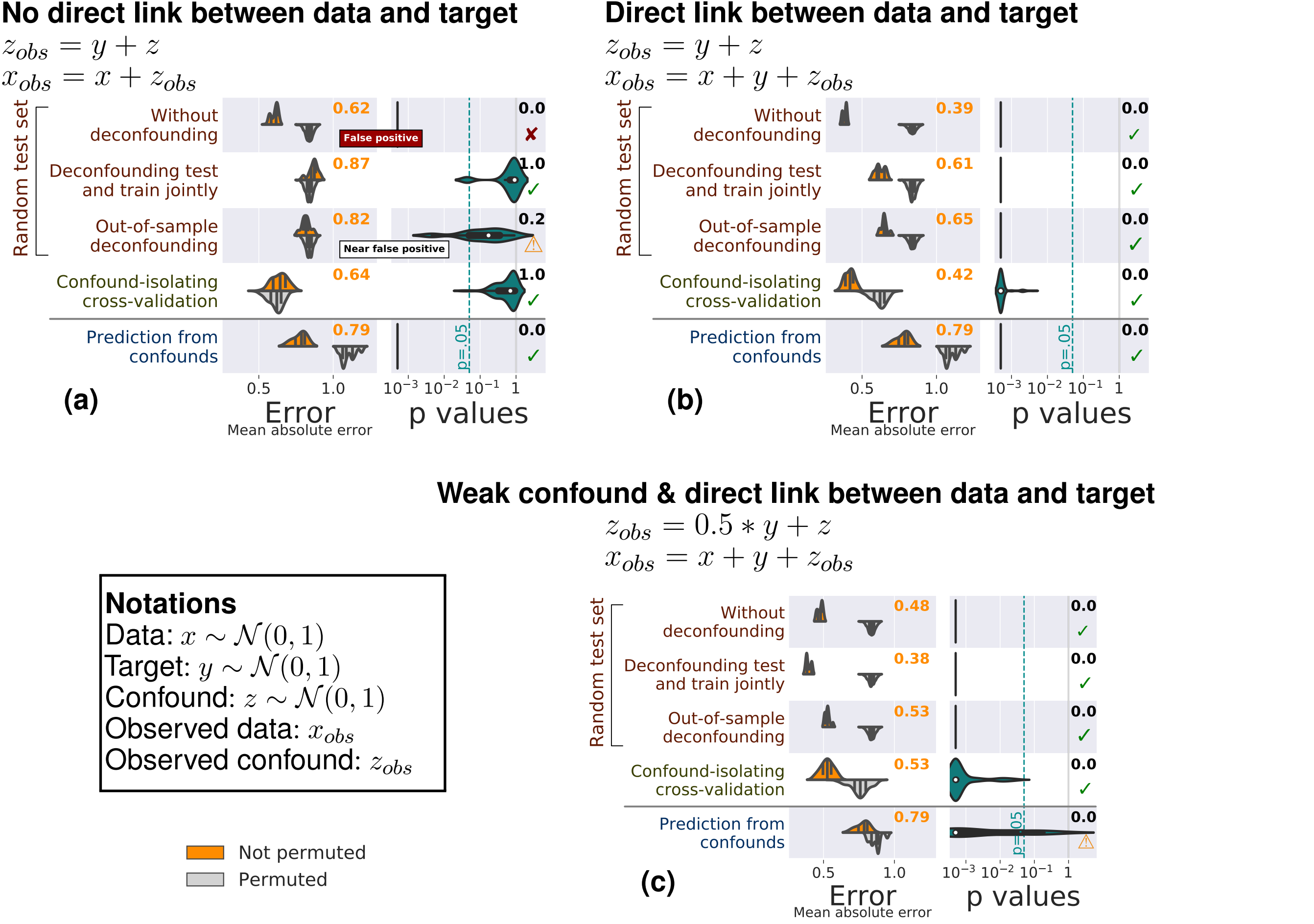
No direct link between data and target
$z_{obs} = y + z$
$x_{obs} = x + z_{obs}$
Random test set
Without deconfounding
Deconfounding test and train jointly
Out-of-sample deconfounding
Confound-isolating cross-validation
Prediction from confounds
0.62
0.87
0.82
0.64
0.79
False positive
Near false positive
0.0
1.0
0.2
1.0
0.0
p=.05
0.5
1.0
10^{-3} 10^{-2} 10^{-1} 1
(a)
Error
Mean absolute error
p values
Direct link between data and target
$z_{obs} = y + z$
$x_{obs} = x + y + z_{obs}$
Random test set
Without deconfounding
Deconfounding test and train jointly
Out-of-sample deconfounding
Confound-isolating cross-validation
Prediction from confounds
0.39
0.61
0.65
0.42
0.79
0.0
0.0
0.0
0.0
0.0
p=.05
0.5
1.0
10^{-3} 10^{-2} 10^{-1} 1
(b)
Error
Mean absolute error
p values
Weak confound & direct link between data and target
$z_{obs} = 0.5 * y + z$
$x_{obs} = x + y + z_{obs}$
Random test set
Without deconfounding
Deconfounding test and train jointly
Out-of-sample deconfounding
Confound-isolating cross-validation
Prediction from confounds
0.48
0.38
0.53
0.53
0.79
0.0
0.0
0.0
0.0
0.0
p=.05
0.5
1.0
10^{-3} 10^{-2} 10^{-1} 1
(c)
Error
Mean absolute error
p values
Notations
Data: $x \sim \mathcal{N}(0, 1)$
Target: $y \sim \mathcal{N}(0, 1)$
Confound: $z \sim \mathcal{N}(0, 1)$
Observed data: x_{obs}
Observed confound: z_{obs}
Not permuted
Permuted
