# Supplementary figures and images for: How to remove or control confounds in predictive models, with applications to brain biomarkers
Source: Gigascience. 2022 Mar 12;11:giac014. doi: 10.1093/gigascience/giac014 (PMC8917515; doi:10.1093/gigascience/giac014)

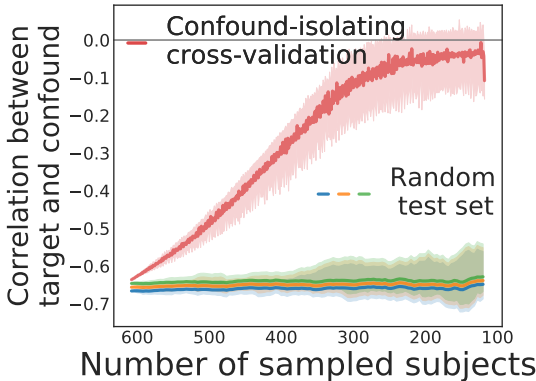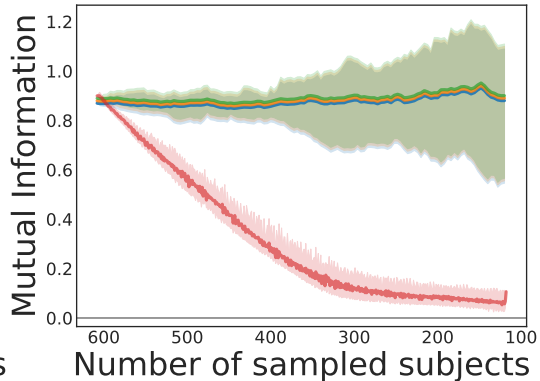

**CamCan, Fluid Intelligence prediction**

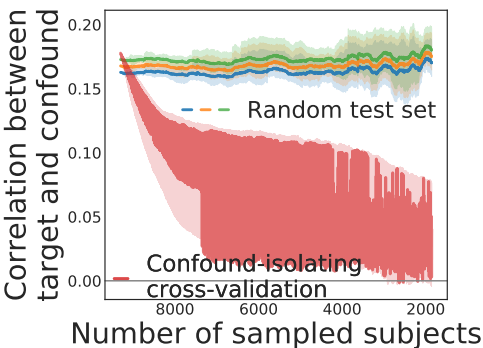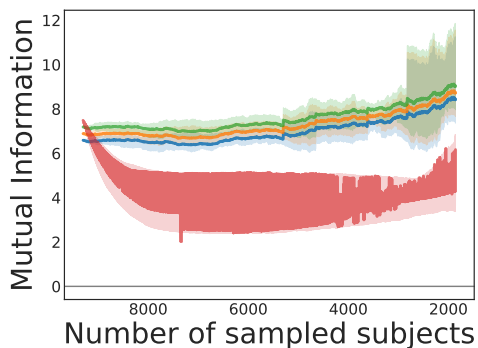

**UKBB, Age prediction**

Supplement: giac014_Supplemental_Files [file giac014_supplemental_files.zip › figures_08_Supplementary Material.pdf]
