# Supplementary material for: How to remove or control confounds in predictive models, with applications to brain biomarkers
Source: Gigascience. 2022 Mar 12;11:giac014. doi: 10.1093/gigascience/giac014 (PMC8917515; doi:10.1093/gigascience/giac014)
Supplement: giac014_Response_to_Reviewer_Comments_Revision_1 [file giac014_response_to_reviewer_comments_revision_1.pdf]

Author's Response To Reviewer Comments

Close

Dear editor and reviewers,

Thank you for reviewing our manuscript, we have revised the manuscript according to your comments and suggestions. Please see the point by point responses to the comments as listed below.

Reviewer #1
I made minor notes on improving the writing on the manuscript. In a few places, where I could not figure out what you meant that has been noted.
- Thank you for providing comments in that form, this was really helpful to us. We have tried to clarify the formulation in places that you outlined.

Generally, the writing was imprecise. Your meaning of confounds was not clear to me. You say "MRI biomarkers of brain aging may be nothing more than expensive measurements of head motion." which can mean that using a feature for head motion will be utilizing a confound. Of course, the head motion will affect many measured image features also. I think you would mean both.
- Indeed. Part of the difficulty comes from the fact that an effect is a “confound” depends on the research question. The scenario that we think of is that people may use brain image features for prediction, ignoring that some these features have little or nothing to do with neural activity, but mostly reflect the motion effect, and yet are predictive of an outcome (say, age). People would then use the motion signal carried by brain data to predict age, which is suboptimal (if motion is the feature of interest, it can be captured more efficiently) and misleading (motion-related information being interpreted as neural feature).

It would be helpful to have a nice, clear description of what you intend to address. You say "procedures need to be adapted to predictive-modelling settings" when talking of confound removal in statistics. Yet, statistics underlies (in one way or another) arguably all learned predictive models. The sentence "For this we introduce confound-isolating cross-validation, sampling test sets in which the effect of interest is independent from the confounding effect." was not helpful. Later we can see you try to get a test set without a confound (assuming you realize there may be one).
- We thank the reviewer for raising this point.
Regarding considerations about statistics vs predictive models, we agree that predictive models are statistical models, but we want to emphasize the distinction between classical statistics, that perform in-sample inference, use extensively the maximum likelihood principle and thus rely heavily on distributional hypotheses, vs predictive models that are weakly parametric and are merely built to optimize accuracy scores. This distinction was probably best characterized by L. Breiman (see reference [18] in the new version).
We have added the following statement: “However, these procedures need to be adapted to high-dimensional predictive-modeling settings, where the focus is to achieve high-prediction accuracy based on imaging data.[…] It is not to identify treatment effects size nor to perform other types of causal inference.”

On page 2, notation gets confusing. You do not tell us what p is. Usually, it would be features. Then z in R^n is trying to say a dimensionless confound is in all the examples, I think. Needs clarity. Trying to look at confounds in CV is interesting especially if it enables good performance on unseen sources (which in medical data can be very challenging unless you have a diverse training set, which I assume you well know but want to note a perspective).
- Point well-taken. We have added the following sentence: “Such prediction may be misleading or useless [...]. Moreover, this can be detrimental to accuracy: if a future dataset shows an altered relation between the confound and the features, the accuracy may be compromised.”

When you talk of re-balancing the data, that is in the case that the confound is highly imbalanced data where predicting the majority class all the time is very accurate, but not useful?

- Not exactly. We consider the case of discrete confounders, where the distribution of the target is non-independent from that of the confounders. Resampling the distributions to reduce imbalance is an effective way of deconfounding, as explained in the Categorical confound section.

When you talk about target population at the bottom of page 2, you mean test population? Seems obvious that if train/test are exactly the same then you have to remove confounds from both, but your comment is unclear when discussing [20].
- We have changed “target population” for “test population”. Note that in [20] (now [22]), the discussion is not about confounding but about covariate shift between train and test samples, thus assuming a simple validation procedure. We have tried to make it clear that we are not addressing the same question as [20]([22]): “However, [22] have shown that compensating for the confound does not improve prediction if the test population is not markedly different from the training population. Note that train and test samples are often drawn from the same population, either because only one cohort is available or if a proper stratification scheme is used. Our question is different: we are interested in knowing whether learning a biomarker on a confounded dataset leads to predictions that are fully driven by the confound.”

For Algorithm 1 and 2, the features (p) are missing. If not an oversight, needs explanation.
- This has been fixed, thanks

Why 4 subjects to discard vs. 2, 3, etc.?
- This is a choice tailored to the sample size considered here: 4 makes the algorithm faster than using 1, yet is low enough not to compromise mutual information minimization.

What are i and i-1 in the equation for z in column 2 on page 4? When you talk about multi-modal MRI data, I think you mean multi-sequence.
- These correspond to the time index of fMRI data, that are time series. We have improved the description here.

Your figures are hard to read. MAE for UKBB is low, but you say it is worse than chance. I think you mean something else. Figures 5 and 7 need better explanation in the text to convince us you have really achieved your goals.
- This is an important point. The “worse than chance” expression comes from a comparison with a permuted distribution. Note that MAE strongly depends on the age span of the observed cohort. This has been clarified in the main text.

Don't know what you mean by: Using deconfounding to condition on a putative confound z help isolating causal links between the data X and the prediction target y, when z is a common cause of X and y.
- Thanks for pointing this. Indeed this formulation was quite impressive and was requiring the reader to adopt a causal perspective, which is not our main aim here. We have rephrased this to better clarify the conceptual shift involved : “Using deconfounding to cancel the impact of a putative confound z removes any bias incurred by the spurious association between the data X and the prediction target y, when z is associated with both X and y.”

Overall, identifying confounds and removing them when doing cross validation can be useful. It is not clear to me what kinds of confounds you can find. Different machines for two classes? Motion (seems so as it is an example)? Sequence differences for classes? Etc. A good, clear takeaway of the impacts and limits would help (yes I know you have done something on this, but it left questions).
- Thanks for raising this, indeed it is worth illustrating the point: confounds can indeed relate to any aspect of the setup (acquisition device, data processing routine when it is not homogeneous across all dataset, measurement-related covariate such as motion, individual conditions, such as age, sex or genetics, that is correlated with the imaging variable and with the outcome. This has been added to the main text.

Reviewer #2
The manuscripts presents a test scheme for validating predictive models named confound-isolating cross-validation, which allows investigators to look at whether a model is fully driven by a confounder or has additional predictive power with respect to the target variable. Experiments include several large-scale datasets including both imaging and non-imaging data. The manuscript is prepared in a good manner. I believe the authors are addressing an important problem of handling confounders in predictive modeling, considering there is a surge of using machine learning to probe neuroscientific

research.
- Thanks for your comments.

Here are some of my suggestions:

The results seem to have valuable information in it, but it becomes very difficult for readers to harmonize the results with the conclusion/discussion. There seems to be a lot of messages being conveyed, but not in a coherent line. This is partly due to the results of different tasks (Figs. 5-7) being so variant. E.g., the text states "Prediction from confounds leads to good prediction of the target in all datasets.", but this is not the case in Fig 5, especially Fig 5c. The discussion states "Out-sample deconfounding give valid information", but how do we know this given that it gives worse-than-chance results in Fig. 5c. Basically the violin plots are so different across methods and tasks, which are not intuitively understandable.
- Indeed the description of the results was sometimes too sketchy. We have striven to make it more accurate. By “valid” we mean that deconfounding is conservative in the worst case, which is certainly suboptimal but not misleading. We have clarified in the main text.
“In the worst case, these approaches can be conservative, but they don't yield spurious associations.”
Please note two additional points:
* the quality of methods can only be asserted based on simulated data, where the ground truth is known.
* the variability of the results can be related to the complex relationship between confounder, data and target: the causal link are not necessarily from confounder to data and target. For this reason, as explained in the main text, deconfounding can be beneficial or detrimental, and it is hard to know in advance what will occur.
We have tried to convey these more clearly in the results section.

It is not clear how the proposed approach can make an impact in the clinical setting. The learned model in the context of CICV is still confounded; i.e., it can not be used to perform diagnosis, pinpoint biomarkers (e.g., find which functional connections predict age), nor hypothesis testing. Even if CICV shows that a model is fully confounded (no additional power), it does not mean there is no relationship between X and y just because the model is not learned correctly. The only benefit I see here is that it can validate whether the model has additional power beyond capturing confounding effect and therefore can compare models, but this is only useful in the machine learning context, not in neuroscience applications.
- The reviewer raises a very important point. Indeed, our only claim is that it is possible to learn a confounded model yet evaluate it in an unbiased fashion. What matters in this logic is that the predictive accuracy after CICV remains better than chance, which amounts to performing an omnibus test of the variables of the model. Note that we explicitly recommend to use deconfounding if the goal is to obtain a model free of confounds.
The case where CICV would yield a null or weak result certainly means that one should be cautious in claiming an association between X and y, as slight variation in the confounding model may render the association significant or not: indeed the apparent association between X and y is dominated by z and is thus spurious. This has been added to the main text.
We think that even in a neuroscience context, practitioners should be made aware that the claimed association between covariates and target is dominated by a confounding effect, and in that sense, spurious. In other words this has an impact on the practical significance of claimed associations.

The proposed concept of using confounder-invariant test set is closely related to concepts of demographic parity in fair machine learning and has been explored in Zhao et al. Training confounder-free models for medical applications.
- Thanks for the reference to this nice piece of work, which we have added to the main text.

Some technical concerns:
the discussion on categorical variables seems to be flawed. Avoiding samples from the same site both in the train and the test sets is the OPPOSITE of having independent site and target. Instead, one should have equal (or proportional) number of control and diseased subjects from each site (i.e. a non-significant chi-square test). In an extremely scenario, if all diseased subjects are from one site, the proposed construction would be fully confounded.
- Thanks for raising this point, which is very important. Actually there are two alternatives strategies here: stratification versus generalization across confounder values. While the first one corresponds to classical deconfounding in statistics, the second one is inspired by the machine learning point of view of generalization across contexts. Generalizing across discrete confounder values is indeed a more

stringent test than stratification. And it is useful, because standard stratification may leave some latent association in the data, which is impossible in the strict generalization approach. For this reason, we recommend it. This has been clarified in the main text: “We note that this procedure is different from the stratification strategy used in classical statistics, but it clearly avoids any bias due to imperfectly corrected association between z and the other variables.”

A confounder, by definition, impacts both X and y, i.e., a 3-way interaction, so removing confounding effects solely using z and X is theoretically questionable (Eqs 1-3).
- Indeed our operational definition of confounders is narrower that the general definition that would allow more complex interactions. We have clarified the point in the main text: “Note that in all this work we assume that the confounder is associated with X and y without creating three ways interactions between X, y and z.”

The generative process in the direct-link scenario in the Simulation studies seems wrong. x and y are still independent, but x_obs and y are dependent. One should instead generate dependent x and y, and generate x_obx directly from x.
- What the reviewer proposes here is indeed a canonical scheme in which the confounder would cause X and y. Since our work is not on causal inference per se, we aim at a statistical procedure that does not require a prescribed causal relationship between the variables (which is often unknown). This point has been made explicit in the main paper. Note that we have updated the notation to ease understanding.

In both old-fashioned statistical methods and modern machine learning, controlling confounders in neuroscience studies still primarily relies on matching confounders, i.e. constructing confounder-free training set instead of confounder-free test set. I wonder why this option is not discussed at all.
- Of course, when it is feasible to construct confounder-free training sets, this approach should be considered, and could actually rely on the procedure we use for the test set creation. However, in our experience this is not feasible, as soon as there are several confounders. We would also like to emphasize this does not circumvent the necessity to validate the model in an unbiased way using procedures like CICV.
We have added in the introduction “We consider that practitioners should primarily avoid or reduce the impact of confounders on their model, but this is not always feasible or hard to check, hence, we choose to put the emphasis on the unbiased evaluation of models even in the presence of confounders.”

Other confusion: in general I feel the figures contain many elements but the caption is concise, so it is hard to parse. The figures suggest CICV uses a model other than random forest, so what is it? What is the statistical test underlying the p-values?
- We have striven to improve the captions by making them more explicit.
CICV uses kernel-density estimation.

Close
