# Supplementary material for: How to remove or control confounds in predictive models, with applications to brain biomarkers
Source: Gigascience. 2022 Mar 12;11:giac014. doi: 10.1093/gigascience/giac014 (PMC8917515; doi:10.1093/gigascience/giac014)
Supplement: giac014_Response_to_Reviewer_Comments_Revision_2 [file giac014_response_to_reviewer_comments_revision_2.pdf]

Author's Response To Reviewer Comments

Close

Reviewer #1: I have mostly minor comments on the writing. Overall, the paper is pretty dense. Confounds are an issue and the authors point out how age can be one in brain imaging and education. Figuring out what the predictive accuracy is without confounds is certainly important. This paper has two approaches to the problem.

- First of all, thanks for taking time to provide detailed on this paper.

When you say link between brain imaging and age do you mean you can tell age in introduction? I think you mean that age affects movement and you can tell from movement the age (from later). Clarity is generally needed throughout this paper.

- There is an important focus of current research in neuroimaging on brain age, i.e. the assessment of the aging process from brain images --- possibly one of the bigger successes of brain imaging in the recent years. All this line of research capitalizes on the possibility to predict age from brain imaging features. The problem is that part of this analysis is confounded by motion, whereby motion during image acquisition increases with age, and yields widespread effects in the data. It is thus important to assess how well age can be predicted from brain imaging beyond mere motion effects.
We have rewritten the sentence in introduction to make it clearer: “For instance, brain imaging reflects age quite accurately, and actually carries information about age-related diseases [8, 11, 12], yet [10] showed that subjects’ in-scanner motion severely affects the link between brain-imaging signals and their age: in-scanner motion varies with subjects’ age and it creates systematic differences in brain signals. Given this confounding effect, MRI biomarkers of brain aging may be nothing more than expensive measurements of head motion.”

nuisance factors have been isolated in one confound variable. -> How would you do this? One has to assume nuisance == confound. Please clarify.

- Sorry, the wording was indeed inadequate here: While there are a priori several nuisance factors, the proposed approach does not make it possible to handle several confounding effects.
We have rephrased the sentence as follows: “assuming that the main confounding factor has been isolated in one variable”
We have added the following sentence in the discussion: “In practice, we recommend to identify the most impactful confound to run confound-isolating cross-validation.”

Is it always going to be possible to isolate the confounding effect in a CV? The statement implies that you know the confound. If so, state it. There are a number of problems where we do not know the confound. This has happened with CXRs quite a bit.

- The reviewer raises an important point here. In general, it is very hard to handle unobserved confounding. The literature on treatment effects estimation shows that rather complex strategies need to be used, relying on additional hypotheses on these confounds. Yet, it may indeed be the case that the main confound is not observed.
We choose to acknowledge this explicitly in the discussion section: “Another concern could be such confounding factors are not well identified In that case, the proposed approach does not help, but such a case is very hard to handle with statistical methods (see e.g. [58]). We thus leave handling of imperfect confounder knowledge for future research.”

A question that remained for me after reading this is how you would remove a confound (or would you) if site related? Obviously segregating cites tells you of confounds, but if one exists you have to solve the problem.

- Indeed sites-related confounds link to potential shifts between the training and test phase (assuming the the cross-validation is based on a leave-site-out principle). Yet in principle, our approach can handle

such cases: we do not attempt to fix confounding at training side, yet make sure that the test set does not exhibit any dependency between target and outcome using the sampling approach. This makes the analysis insensitive to shifts between train and test.
Nevertheless, the main text indicates “Note that in all this work we assume that the confounder is associated with X and y without creating three ways interactions between X, y and z.”, which precludes the case mentioned by the reviewer.
For completeness, we added the following statement “In the case of site-related confounds, prediction accuracy will obviously suffer. This can be addressed with techniques such as invariant risk minimization [28], but we do not further consider this approach here.”

Your k-fold approach seems unnecessarily confusing with standard CV. You are really creating a set (say s) of test sets that are not necessarily non-overlapping. They might not be unique? Later you say they could all be the same, but clarity up front would help the reader.

- One of our goals with this paper is to raise the attention of practitioners toward cross-validation design choices, that are quite often handled as a routine.
The reviewer is raising an important concern, namely that there may be degenerate cases (e.g. if the association between target and confounder is very strong) where there is not enough variability in the test set obtained by sampling.
We have made the point more explicit in the discussion by adding the following to the “A sampling view on confounds” paragraph: “The only caveat is that one has to ensure that sampling does not deterministically lead to a fixed test set, which would weaken the statistical guarantees brought by the validation experiment. Here, we propose to perform this check a posteriori. In the future, more complex sampling strategies could be designed to ensure some randomness in the test set.”
We have also emphasize the point in the methods section.

This work depends on knowing what the confound z is. In many imaging problems there appear to be confounds because performance is not the same for a different site, image capture settings, etc., but we do not know what they are. So, it would be helpful to note this work requires knowing or being able to estimate the confound. The confusion for me is that all of my work deals with confounds that are not like age and are unknown, so finding them really matters and then I guess we might use your approach.

- This perfectly true, we have thus made the point more explicit in the discussion:
“Another concern could be such confounding factors are not well identified In that case, the proposed approach does not help, but such a case is very hard to handle with statistical methods (see e.g. [58]).”

w^ is an estimate of w which should be explicitly said.

- Sure, when first using the notation, we indicate that “\hat{w} represents the estimated coefficients, that are obtained typically through least-squares regression”.

In algorithm 2: f seems to have two arguments, but then 1. I think you mean that f becomes the trained model and then you use z_test for testing. So, g produces values for each feature or a model taking all features?

- f only has one argument, but its estimation requires fitting a model g with 2 arguments (one is the input, the second is the output). We have added the following sentence in the caption of Alg. 2: Note that f only has one argument, as it predicts X from z, while g has two arguments (the input X and the output z), as it represents the learning algorithm that yields f.

Maybe someone could figure it all out from code, but first they have to decide this approach is useful. It might be if you can make it clear.

- We have reorganized a bit the paper and simplified the writing for the sake of clarity.

All results are in figures that are complex and hard to read. Your MAE is generally low and yet you say some is worse than random which is odd. I think most readers would prefer some tabular results with a clear explanation. The overall takeaway points are not clearly made and the paper needs to be written more clearly so non-experts can benefit from it.

- Thanks for your suggestion. We have added table 2 that summarizes our experimental results, and indeed synthesizes the results we obtained. The MAE we report are not much different from values

observed in the literature on this type of data.

Reviewer #2: The authors adequately answered to my questions. Last sanity check, is it 608 or 626 subjects in Figure 2?

- Thank you for checking, we ran experiments on 626 participants from the CamCan dataset.

Thanks for all the detailed comments in the pdf paper. We have taken into account and hope that the reviewer will approve the changes we made.

Close
