# Supplementary material for: How to remove or control confounds in predictive models, with applications to brain biomarkers
Source: Gigascience. 2022 Mar 12;11:giac014. doi: 10.1093/gigascience/giac014 (PMC8917515; doi:10.1093/gigascience/giac014)
Supplement: giac014_Response_to_Reviewer_Comments_Revision_3 [file giac014_response_to_reviewer_comments_revision_3.pdf]

Author's Response To Reviewer Comments

Close

Dear Hans,

Thank you for proceeding so quickly.
We have made all asked corrections and our manuscript is revised and ready for publication.

Best regards,
Darya

Close
