# Supplementary material for: How to remove or control confounds in predictive models, with applications to brain biomarkers
Source: Gigascience. 2022 Mar 12;11:giac014. doi: 10.1093/gigascience/giac014 (PMC8917515; doi:10.1093/gigascience/giac014)
Supplement: giac014_Reviewer_1_Report_Original_Submission — Lawrence Hall -- 6/14/2021 Reviewed [file giac014_reviewer_1_report_original_submission.pdf]

Reviewer Report

Title: How to remove or control confounds in predictive models, with applications to brain biomarkers.

Version: Original Submission **Date:** 6/14/2021

Reviewer name: Lawrence Hall

Reviewer Comments to Author:

I made minor notes on improving the writing on the manuscript. In a few places, where I could not figure out what you meant that has been noted.
Generally, the writing was imprecise. Your meaning of confounds was not clear to me. You say "MRI biomarkers of brain aging may be nothing more than expensive measurements of head motion." which can mean that using a feature for head motion will be utilizing a confound. Of course, the head motion will affect many measured image features also. I think you would mean both. It would be helpful to have a nice, clear description of what you intend to address. You say "procedures need to be adapted to predictive-modelling settings" when talking of confound removal in statistics. Yet, statistics underlies (in one way or another) arguably all learned predictive models. The sentence "For this we introduce confound-isolating cross-validation, sampling test sets in which the effect of interest is independent from the confounding effect." was not helpful. Later we can see you try to get a test set without a confound (assuming you realize there may be one).
On page 2, notation gets confusing. You do not tell us what p is. Usually, it would be features. Then z in R^n is trying to say a dimensionless confound is in all the examples, I think. Needs clarity. Trying to look at confounds in CV is interesting especially if it enables good performance on unseen sources (which in medical data can be very challenging unless you have a diverse training set, which I assume you well know but want to note a perspective). When you talk of re-balancing the data, that is in the case that the confound is highly imbalanced data where predicting the majority class all the time is very accurate, but not useful? When you talk about target population at the bottom of page 2, you mean test population? Seems obvious that if train/test are exactly the same then you have to remove confounds from both, but your comment is unclear when discussing [20].
For Algorithm 1 and 2, the features (p) are missing. If not an oversight, needs explanation. Why 4 subjects to discard vs. 2, 3, etc.? What are i and i-1 in the equation for z in column 2 on page 4? When you talk about multi-modal MRI data, I think you mean multi-sequence.
Your figures are hard to read. MAE for UKBB is low, but you say it is worse than chance. I think you mean something else. Figures 5 and 7 need better explanation in the text to convince us you have really achieved your goals.
Don't know what you mean by: Using deconfounding to condition on a putative confound z help isolating causal links between the data X and the prediction target y, when z is a common cause of X and y.
Overall, identifying confounds and removing them when doing cross validation can be useful. It is not clear to me what kinds of confounds you can find. Different machines for two classes? Motion (seems so

as it is an example)? Sequence differences for classes? Etc. A good, clear takeaway of the impacts and limits would help (yes I know you have done something on this, but it left questions).

Methods

Are the methods appropriate to the aims of the study, are they well described, and are necessary controls included? Choose an item.

Conclusions

Are the conclusions adequately supported by the data shown? Choose an item.

Reporting Standards

Does the manuscript adhere to the journal's guidelines on minimum standards of reporting? Choose an item.

Choose an item.

Statistics

Are you able to assess all statistics in the manuscript, including the appropriateness of statistical tests used? Choose an item.

Quality of Written English

Please indicate the quality of language in the manuscript: Choose an item.

Declaration of Competing Interests

Please complete a declaration of competing interests, considering the following questions:

- Have you in the past five years received reimbursements, fees, funding, or salary from an organisation that may in any way gain or lose financially from the publication of this manuscript, either now or in the future?
- Do you hold any stocks or shares in an organisation that may in any way gain or lose financially from the publication of this manuscript, either now or in the future?
- Do you hold or are you currently applying for any patents relating to the content of the manuscript?
- Have you received reimbursements, fees, funding, or salary from an organization that holds or has applied for patents relating to the content of the manuscript?
- Do you have any other financial competing interests?
- Do you have any non-financial competing interests in relation to this paper?

If you can answer no to all of the above, write 'I declare that I have no competing interests' below. If your reply is yes to any, please give details below.

I declare that I have no competing interests.

I agree to the open peer review policy of the journal. I understand that my name will be included on my report to the authors and, if the manuscript is accepted for publication, my named report including any attachments I upload will be posted on the website along with the authors' responses. I agree for my report to be made available under an Open Access Creative Commons CC-BY license (http://creativecommons.org/licenses/by/4.0/). I understand that any comments which I do not wish to be included in my named report can be included as confidential comments to the editors, which will not be published.

Choose an item.

To further support our reviewers, we have joined with Publons, where you can gain additional credit to further highlight your hard work (see: https://publons.com/journal/530/gigascience). On publication of this paper, your review will be automatically added to Publons, you can then choose whether or not to claim your Publons credit. I understand this statement.

Yes Choose an item.
