# Supplementary material for: How to remove or control confounds in predictive models, with applications to brain biomarkers
Source: Gigascience. 2022 Mar 12;11:giac014. doi: 10.1093/gigascience/giac014 (PMC8917515; doi:10.1093/gigascience/giac014)
Supplement: giac014_Reviewer_1_Report_Revision_1 — Lawrence Hall -- 9/30/2021 Reviewed [file giac014_reviewer_1_report_revision_1.pdf]

Reviewer Report

Title: How to remove or control confounds in predictive models, with applications to brain biomarkers.

Version: Revision 1 **Date:** 9/30/2021

Reviewer name: Lawrence Hall

Reviewer Comments to Author:

I have mostly minor comments on the writing. Overall, the paper is pretty dense. Confounds are an issue and the authors point out how age can be one in brain imaging and education. Figuring out what the predictive accuracy is without confounds is certainly important. This paper has two approaches to the problem.

When you say link between brain imaging and age do you mean you can tell age in introduction? I think you mean that age affects movement and you can tell from movement the age (from later). Clarity is generally needed throughout this paper.

nuisance factors have been isolated in one confound variable. -> How would you do this? One has to assume nuisance == confound. Please clarify.

Is it always going to be possible to isolate the confounding effect in a CV? The statement implies that you know the confound. If so, state it. There are a number of problems where we do not know the confound. This has happened with CXRs quite a bit.

A question that remained for me after reading this is how you would remove a confound (or would you) if site related? Obviously segregating cites tells you of confounds, but if one exists you have to solve the problem

Your k-fold approach seems unnecessarily confusing with standard CV. You are really creating a set (say s) of test sets that are not necessarily non-overlapping. They might not be unique? Later you say they could all be the same, but clarity up front would help the reader.

This work depends on knowing what the confound z is. In many imaging problems there appear to be confounds because performance is not the same for a different site, image capture settings, etc., but we do not know what they are. So, it would be helpful to note this work requires knowing or being able to estimate the confound. The confusion for me is that all of my work deals with confounds that are not like age and are unknown, so finding them really matters and then I guess we might use your approach.

w^ is an estimate of w which should be explicitly said.

In algorithm 2: f seems to have two arguments, but then 1. I think you mean that f becomes the trained model and then you use z_test for testing. So, g produces values for each feature or a model taking all features?

Maybe someone could figure it all out from code, but first they have to decide this approach is useful. It might be if you can make it clear.

All results are in figures that are complex and hard to read. Your MAE is generally low and yet you say some is worse than random which is odd. I think most readers would prefer some tabular results with a clear explanation. The overall takeaway points are not clearly made and the paper needs to be written

more clearly so non-experts can benefit from it.

Methods

Are the methods appropriate to the aims of the study, are they well described, and are necessary controls included? Choose an item.

Conclusions

Are the conclusions adequately supported by the data shown? Choose an item.

Reporting Standards

Does the manuscript adhere to the journal's guidelines on minimum standards of reporting? Choose an item.

Choose an item.

Statistics

Are you able to assess all statistics in the manuscript, including the appropriateness of statistical tests used? Choose an item.

Quality of Written English

Please indicate the quality of language in the manuscript: Choose an item.

Declaration of Competing Interests

Please complete a declaration of competing interests, considering the following questions:

- Have you in the past five years received reimbursements, fees, funding, or salary from an organisation that may in any way gain or lose financially from the publication of this manuscript, either now or in the future?
- Do you hold any stocks or shares in an organisation that may in any way gain or lose financially from the publication of this manuscript, either now or in the future?
- Do you hold or are you currently applying for any patents relating to the content of the manuscript?
- Have you received reimbursements, fees, funding, or salary from an organization that holds or has applied for patents relating to the content of the manuscript?
- Do you have any other financial competing interests?
- Do you have any non-financial competing interests in relation to this paper?

If you can answer no to all of the above, write 'I declare that I have no competing interests' below. If your reply is yes to any, please give details below.

I declare I have no competing interests.

I agree to the open peer review policy of the journal. I understand that my name will be included on my report to the authors and, if the manuscript is accepted for publication, my named report including any attachments I upload will be posted on the website along with the authors' responses. I agree for my report to be made available under an Open Access Creative Commons CC-BY license (http://creativecommons.org/licenses/by/4.0/). I understand that any comments which I do not wish to be included in my named report can be included as confidential comments to the editors, which will not be published.

Choose an item.

To further support our reviewers, we have joined with Publons, where you can gain additional credit to further highlight your hard work (see: https://publons.com/journal/530/gigascience). On publication of this paper, your review will be automatically added to Publons, you can then choose whether or not to claim your Publons credit. I understand this statement.

Yes Choose an item.
