# Supplementary material for: How to remove or control confounds in predictive models, with applications to brain biomarkers
Source: Gigascience. 2022 Mar 12;11:giac014. doi: 10.1093/gigascience/giac014 (PMC8917515; doi:10.1093/gigascience/giac014)
Supplement: giac014_Reviewer_2_Report_Original_Submission — Qingyu Zhao -- 6/22/2021 Reviewed [file giac014_reviewer_2_report_original_submission.pdf]

Reviewer Report

Title: How to remove or control confounds in predictive models, with applications to brain biomarkers.

Version: Original Submission **Date:** 6/22/2021

Reviewer name: Qingyu Zhao

Reviewer Comments to Author:

The manuscripts presents a test scheme for validating predictive models named confound-isolating cross-validation, which allows investigators to look at whether a model is fully driven by a confounder or has additional predictive power with respect to the target variable. Experiments include several large-scale datasets including both imaging and non-imaging data. The manuscript is prepared in a good manner. I believe the authors are addressing an important problem of handling confounders in predictive modeling, considering there is a surge of using machine learning to probe neuroscientific research. Here are some of my suggestions:

- The results seem to have valuable information in it, but it becomes very difficult for readers to harmonize the results with the conclusion/discussion. There seems to be a lot of messages being conveyed, but not in a coherent line. This is partly due to the results of different tasks (Figs. 5-7) being so variant. E.g., the text states "Prediction from confounds leads to good prediction of the target in all datasets.", but this is not the case in Fig 5, especially Fig 5c. The discussion states "Out-sample deconfounding give valid information", but how do we know this given that it gives worse-than-chance results in Fig. 5c. Basically the violin plots are so different across methods and tasks, which are not intuitively understandable.
- It is not clear how the proposed approach can make an impact in the clinical setting. The learned model in the context of CICV is still confounded; i.e., it can not be used to perform diagnosis, pinpoint biomarkers (e.g., find which functional connections predict age), nor hypothesis testing. Even if CICV shows that a model is fully confounded (no additional power), it does not mean there is no relationship between X and y just because the model is not learned correctly. The only benefit I see here is that it can validate whether the model has additional power beyond capturing confounding effect and therefore can compare models, but this is only useful in the machine learning context, not in neuroscience applications.
- The proposed concept of using confounder-invariant test set is closely related to concepts of demographic parity in fair machine learning and has been explored in Zhao et al. Training confounder-free models for medical applications.

Some technical concerns:

- the discussion on categorical variables seems to be flawed. Avoiding samples from the same site both in the train and the test sets is the OPPOSITE of having independent site and target. Instead, one should have equal (or proportional) number of control and diseased subjects from each site (i.e. a non-significant chi-square test). In an extremely scenario, if all diseased subjects are from one site, the proposed construction would be fully confounded.

- A confounder, by definition, impacts both X and y, i.e., a 3-way interaction, so removing confounding effects solely using z and X is theoretically questionable (Eqs 1-3).
- The generative process in the direct-link scenario in the Simulation studies seems wrong. x and y are still independent, but x_obs and y are dependent. One should instead generate dependent x and y, and generate x_obx directly from x.
- In both old-fashioned statistical methods and modern machine learning, controlling confounders in neuroscience studies still primarily relies on matching confounders, i.e. constructing confounder-free training set instead of confounder-free test set. I wonder why this option is not discussed at all.
Other confusion: in general I feel the figures contain many elements but the caption is concise, so it is hard to parse. The figures suggest CICV uses a model other than random forest, so what is it? What is the statistical test underlying the p-values?

Methods

Are the methods appropriate to the aims of the study, are they well described, and are necessary controls included? Choose an item.

Conclusions

Are the conclusions adequately supported by the data shown? Choose an item.

Reporting Standards

Does the manuscript adhere to the journal's guidelines on [minimum standards of reporting?]() Choose an item.

Choose an item.

Statistics

Are you able to assess all statistics in the manuscript, including the appropriateness of statistical tests used? Choose an item.

Quality of Written English

Please indicate the quality of language in the manuscript: Choose an item.

Declaration of Competing Interests

Please complete a declaration of competing interests, considering the following questions:

- Have you in the past five years received reimbursements, fees, funding, or salary from an organisation that may in any way gain or lose financially from the publication of this manuscript, either now or in the future?

- Do you hold any stocks or shares in an organisation that may in any way gain or lose financially from the publication of this manuscript, either now or in the future?
- Do you hold or are you currently applying for any patents relating to the content of the manuscript?
- Have you received reimbursements, fees, funding, or salary from an organization that holds or has applied for patents relating to the content of the manuscript?
- Do you have any other financial competing interests?
- Do you have any non-financial competing interests in relation to this paper?

If you can answer no to all of the above, write 'I declare that I have no competing interests' below. If your reply is yes to any, please give details below.

No competing interest.

I agree to the open peer review policy of the journal. I understand that my name will be included on my report to the authors and, if the manuscript is accepted for publication, my named report including any attachments I upload will be posted on the website along with the authors' responses. I agree for my report to be made available under an Open Access Creative Commons CC-BY license (http://creativecommons.org/licenses/by/4.0/). I understand that any comments which I do not wish to be included in my named report can be included as confidential comments to the editors, which will not be published.

Choose an item.

To further support our reviewers, we have joined with Publons, where you can gain additional credit to further highlight your hard work (see: https://publons.com/journal/530/gigascience). On publication of this paper, your review will be automatically added to Publons, you can then choose whether or not to claim your Publons credit. I understand this statement.

Yes Choose an item.
